# Origins of high catalyst loading in copper(i)-catalysed Ullmann–Goldberg C–N coupling reactions†
†Electronic supplementary information (ESI) available. See DOI: 10.1039/c7sc02859h


**DOI:** 10.1039/c7sc02859h

**Published:** 2017-08-29

**Authors:** Grant J. Sherborne, Sven Adomeit, Robert Menzel, Jabor Rabeah, Angelika Brückner, Mark R. Fielding, Charlotte E. Willans, Bao N. Nguyen

**Affiliations:** a Institute of Process Research & Development , School of Chemistry , University of Leeds , Woodhouse Lane , Leeds , LS2 9JT , UK . Email: b.nguyen@leeds.ac.uk; b Leibniz Institut für Katalyse e.V , Albert-Einstein Straβe 29a , 18059 , Rostock , Germany; c AstraZeneca , Pharmaceutical Technology and Development , Etherow T41/18, Silk Road Business Park, Charter Way , Macclesfield , SK10 2NA , UK

## Abstract

A mechanistic investigation of Ullmann–Goldberg reactions using soluble and partially soluble bases led to the identification of various pathways for catalyst deactivation through (i) product inhibition with amine products, (ii) by-product inhibition with inorganic halide salts, and (iii) ligand exchange by soluble carboxylate bases. The reactions using partially soluble inorganic bases showed variable induction periods, which are responsible for the reproducibility issues in these reactions. Surprisingly, more finely milled Cs_2_CO_3_ resulted in a longer induction period due to the higher concentration of the deprotonated amine/amide, leading to suppressed catalytic activity. These results have significant implications on future ligand development for the Ullmann–Goldberg reaction and on the solid form of the inorganic base as an important variable with mechanistic ramifications in many catalytic reactions.

## Abstract 2

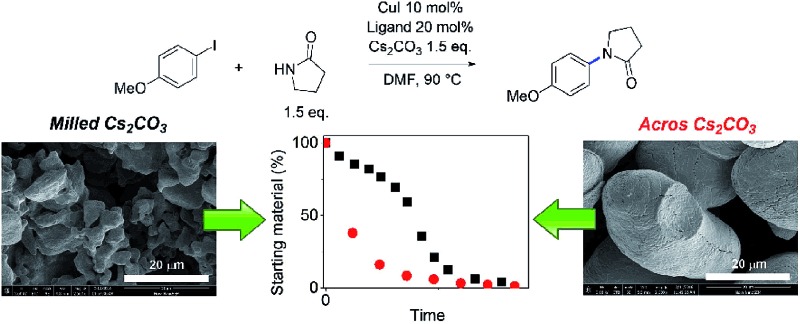
A mechanistic investigation of Ullmann–Goldberg reactions using common bases led to the identification of pathways for catalyst deactivation. The solid form of the inorganic phase was found to have critical influence on the mechanism of the reaction.

## Introduction

Metal catalysed C–N couplings are amongst the most important types of catalytic reaction in modern synthetic chemistry.^1^ These reactions are often catalysed by palladium catalysts.^2^–^10^ However, copper(i)-catalysed C–N coupling reactions, also known as Ullmann–Goldberg reactions,^11^,^12^ have gained significant attention from industry and academia alike. A contemporary drive for cheaper and more sustainable catalysts, combined with improvements of the Ullmann–Goldberg reaction to lower the required temperature and to broaden the range of substrates, have made this a viable alternative to the palladium-catalysed reactions.^13^–^15^

**Scheme 1:**
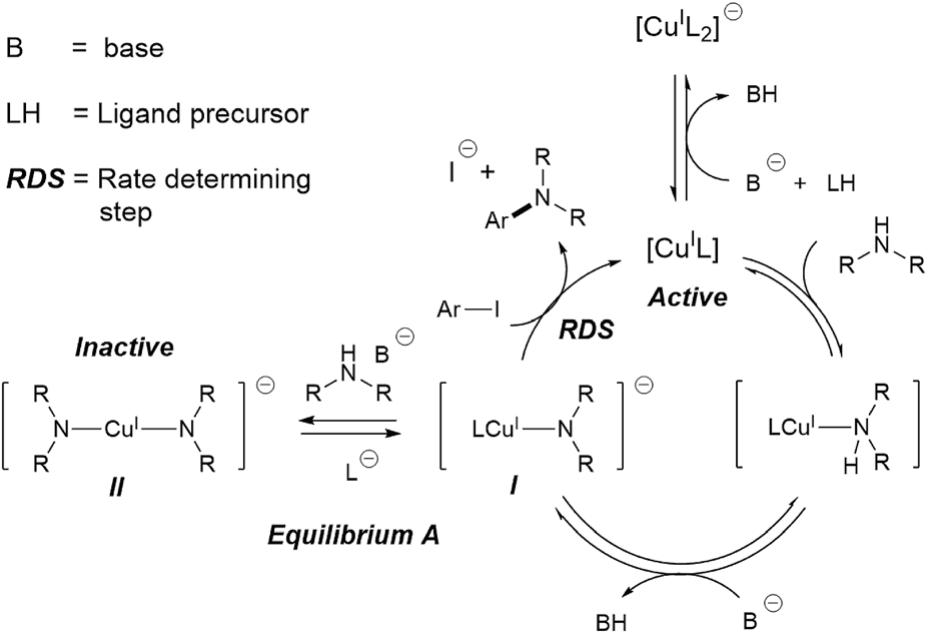
Currently accepted mechanism of the Ullmann–Goldberg reaction.

Since the pivotal work by the Buchwald group on ligand-assisted Ullmann–Goldberg coupling,^16^–^19^ a variety of effective chelating ligands such as ethylene glycol,^18^l-proline,^20^*N*,*N*-dimethyl glycine^20^ and *N*,*N*-diethylsalicylamide^21^ have been reported for *N*-arylation of both aliphatic and aryl amines. Mechanistic studies of Ullmann–Goldberg reactions have been carried out using diamines and diketones as ligands by Buchwald,^22^,^23^ Blackmond,^22^ Hartwig,^24^–^26^ Norrby^27^,^28^ and Davies,^29^,^30^ and the mechanism is summarised in Scheme 1. The reaction starts with coordination of the *N*-partner, followed by its deprotonation by the mild base. Reaction between this complex **I** and the aryl halide releases the amine product and completes the catalytic cycle.^31^,^32^ Several different types of mechanism, *e.g.* oxidative addition, SET and atom transfer,^25^,^26^,^33^,^34^ have been proposed for this rate-determining-step. The overall rate law of the reaction at high ligand loading is showed in eqn (1).

1 rate ≈ *k*_obs_[CuL][ArI][HNR_2_]

Importantly, equilibrium A^22^,^35^ results in a need for high ligand loading to protect the active catalytic species.^22^,^35^ More recent studies by Davies and co-workers identified even more nuances in the role of the *O*,*O*-chelating ligand 2-acetylcyclohexanone, which is easily displaced by the malonate anion (included as a base), and the formation of di-, tri-, and tetrameric Cu(i)-amido species.^29^,^30^ The high catalyst/ligand loading (typically 10 mol% of Cu(i) precursor and 20 mol% of ligand; reactions with a catalyst loading below 1 mol% are rare),^36^,^37^ its poor reproducibility,^38^ and the need for aryl iodide/bromide substrates remain important challenges in these reactions.

In this paper, we report the results of our experimental investigations into the origin of high catalyst loading and poor reproducibility, which are crucial in improving catalytic performance and substrate scope in Ullmann–Goldberg reactions. In particular, the role of the bases, products and halide salts as by-products on the speciation of catalytic species and reaction mechanisms were evaluated in detail. These also led to the identification of the origin of the poor reproducibility in these reactions and a counter-intuitive relationship between the particle size of the inorganic base and the reaction rate. These mechanistic insights form the basis for future development of more effective Ullmann–Goldberg catalytic systems and highlight the need to consider the solid form of the inorganic base as an important variable in many catalytic reactions.

## Results and discussion

### Homogeneous reactions

**Scheme 2:**
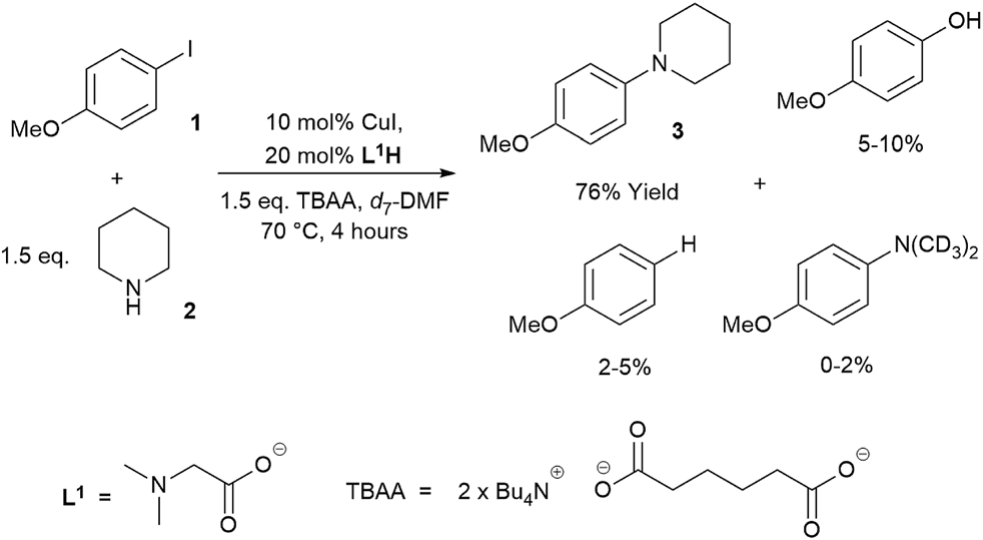
Reaction scheme and general conditions of the *in situ*^1^H NMR kinetic study.

Low turnover numbers and reproducibility issues are often associated with catalyst deactivation.^30^ However, little data can be found on catalyst deactivation in these reactions. Thus, an Ullmann–Goldberg reaction between 4-iodoanisole (**1**) and piperidine (**2**), using soluble tetrabutylammonium adipate (TBAA) as a base,^39^ was selected for our study. This system simplifies phasic behaviour and allows us to focus on any intrinsic catalyst deactivation (Scheme 2). The reaction was found to be extremely air-sensitive and unsuitable for sampling. Thus, *in situ*^1^H NMR was used to monitor the reaction kinetics. A key advantage of this over the often reported calorimetry-based kinetics of the Ullmann–Goldberg reaction is that it allows quantification of side products which may be present in small quantities and easily removed during workup.^22^,^23^,^30^ These were determined to be anisole, 4-methoxy-*N*,*N*-dimethylaniline (through hydrolysis/acyl transfer of DMF)^40^,^41^ and 4-methoxyphenol (see ESI† for characterization of the side products). While each of these was present in a small quantity, their combined yield (up to 15%) can create a significant error in the rate of formation of **3** if this was only measured by calorimetry. A small quantity (∼1%) of 4-chloroanisole was also identified. This product was attributed to chloride contamination in tetrabutylammonium hydroxide, which was used to prepare the TBAA base.^42^

The formation of 4-hydroxyanisole could be explained by the presence of a trace amount of water in the highly hygroscopic TBAA. Hydrodehalogenation products such as anisole were also observed by Hartwig and co-workers.^24^,^26^,^43^ In addition, hydrodehalogenation by Cu(i)-catalysts has been demonstrated under acidic conditions^44^ or in the presence of a proton source.^45^,^46^ Thus, the formation of anisole in our reaction could be attributed to the generation of soluble mono- or diprotonated adipate as by-products.

**Fig. 1:**
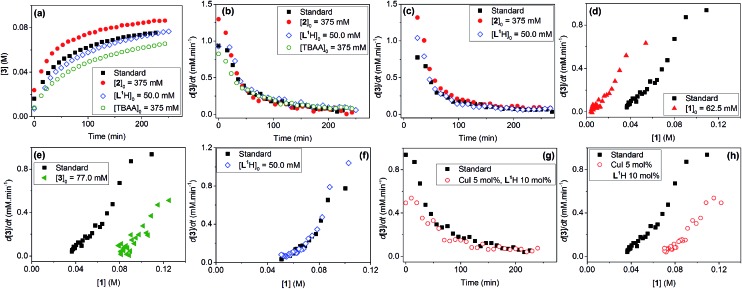
Kinetic data for the reactions from **1** and **2** to **3**. Standard conditions: [**1**]_0_ = 125.0 mM, [**2**]_0_ = 187.5 mM, [**L^1^**H]_0_ = 25.0 mM, [CuI]_0_ = 12.5 mM, [TBAA]_0_ = 187.5 mM in *d*_7_-DMF. (a) [**3**] *vs.* time; (b) rate d[**3**]/d*t vs.* time; (c) rate d[**3**]/d*t vs.* time in *d*_3_-MeCN; (d) rate d[**3**]/d*t vs.* [**1**] in the ‘same excess’ experiments showing catalyst deactivation; (e) rate d[**3**]/d*t vs.* [**1**] showing product inhibition; (f) rate d[**3**]/d*t vs.* [**1**] showing the reaction rate independence from [**L^1^**H]_0_; (g) rate d[**3**]/d*t vs.* time showing the dependence of the reaction rate on catalyst loading; and (h) rate d[**3**]/d*t vs.* [**1**] showing the dependence of the reaction rate on catalyst loading.

Evaluation of the effect of [**L^1^**] showed little change to the kinetics (within the error margin of the technique, *i.e.* ±5%, Fig. 1a, b and f). This is consistent with suggestions from Davies and co-workers that their diketone ligand is replaced by the malonate or amido anion, and that the reaction proceeds in a predominantly ‘ligandless’ manner.^29^,^30^ Doubling [**2**] led to a non-linear increase in the initial rate as expected due to its triple role as substrate, ligand and inhibitor.^22^ On the other hand, doubling [TBAA]_0_ led to a small decrease in the initial reaction rate (Fig. 1b). This can be attributed to ligand exchange between **L^1^** and TBAA, which results in a less active catalyst. The reaction performed without ligand **L^1^** gave 18% conversion after 5 hours, compared to the 80% conversion obtained in the presence of 20 mol% of **L^1^**H at the same reaction time. Importantly, the difference in initial rate quickly gave way to a very similar kinetic profile after 50 minutes and about 40–50% conversion under standard conditions (Fig. 1b), suggesting a change in the dominant reaction mechanism to a slower pathway.

Similar kinetic behaviour and reaction rates were also observed for reactions performed in *d*_3_-acetonitrile as the solvent under similar conditions (Fig. 1c). The most significant change when replacing *d*_7_-DMF with *d*_3_-MeCN is a decrease in the amount of side products to ∼7%. This is likely due to the fewer side reactions associated with the hydrolysis/acyl transfer of DMF.

The decrease in reaction rate after approximately 50 minutes led us to profile a ‘same excess’ experiment starting at [**1**] = 62.5 mM and [**2**] = 125 mM instead of 125 mM and 187.5 mM, respectively, and without added (by)-products (Fig. 1d).^47^,^48^ The d[**3**]/d*t vs.* [**1**] plot for this experiment compared to that for the reaction under standard conditions showed strong evidence of a catalyst deactivation process as the reaction progresses. The plot did not go through the origin point (0,0) under standard conditions, and the difference in reaction rates at [**1**] = 62.5 mM can only be explained by catalyst deactivation. Deactivation was similarly observed with the reaction using malonate as the base,^30^ and was attributed to a possible Cu(i) to Cu(0/ii) disproportionation process reported by Lei *et al.* with acetylacetonate as the ligand.^49^ As our ligand **L^1^** and the adipate base are quite different to those in that study,^30^ we probed a more obvious deactivation pathway through product inhibition. Product **3**, [**3**]_0_ = 77.0 mM (0.62 eq.), was included in a standard reaction at *t* = 0 and data for the reaction rate *vs.* [**1**] are plotted against that of the standard reaction in Fig. 1e. This led to an approximately 2 times decrease in the initial reaction rate, confirming the inhibitive effect of product **3** on this reaction.

The initial rate of the reaction showed a linear dependence on the catalyst loading before catalyst deactivation becomes prominent (Fig. 1g and h), in agreement with eqn (1). Unfortunately, these various kinetic behaviours and the possibility of more than one productive catalytic pathway, *e.g.* with **L^1^** or adipate as the ligand, giving product **3** and side products, precluded any statistically meaningful kinetic modelling in this system (independent variables *vs.* data points).

### Heterogeneous reaction with inorganic bases

**Scheme 3:**
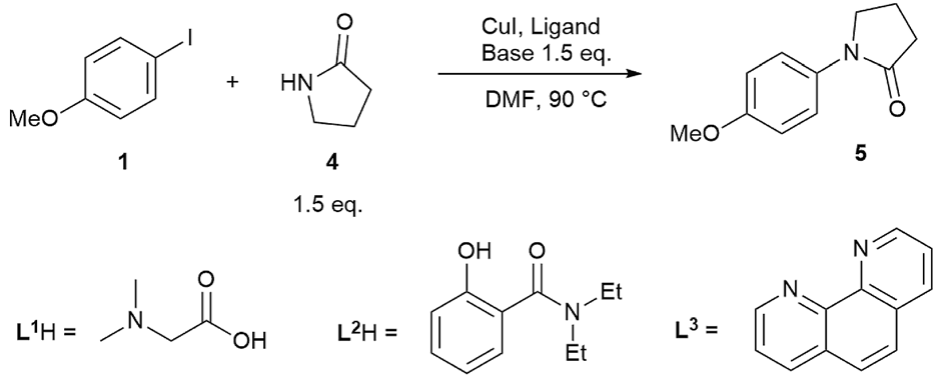
Reactions and conditions for the kinetic studies using Cs_2_CO_3_ and K_3_PO_4_ as bases.

While reactions employing homogeneous organic bases are ‘simpler’ to study, due to no complications associated with solubility and multiphasic processes, the commonly employed bases in Cu(i)-catalysed C–N cross-coupling reactions are inorganic salts such as Cs_2_CO_3_, K_2_CO_3_ and K_3_PO_4_, particularly in coupling reactions with amides instead of amines.^14^,^50^ Thus, we carried out a kinetic study of three representative reactions as outlined in Scheme 3, using ligands **L^1^** and **L^2^**, which have been under-represented in prior mechanistic investigations,^22^–^30^ in addition to the more established **L^3^**. The reactions were performed in a fully inert environment with automated sampling under nitrogen.

A noticeable difference compared to the homogeneous reaction above is the lack of side products in the reactions employing inorganic bases (shown by GC, LC and by ^1^H NMR post work-up), despite the higher reaction temperature, *i.e.* 90 °C compared to 70 °C. When dibasic or tribasic inorganic salts are employed, the generated protons are captured in a bicarbonate or hydrogen phosphate solid (*vide infra*), leading to few side products. In addition, little product inhibition was observed with the **L^1^**H/Cs_2_CO_3_ and **L^2^**H/K_3_PO_4_ combinations when product **5** was included at the beginning of the reaction (see ESI, Fig. S18†). The tertiary amide product **5** is not expected to coordinate well with the Cu(i) species in this reaction, unlike product **3** in the homogeneous reaction in Scheme 2.

**Fig. 2:**
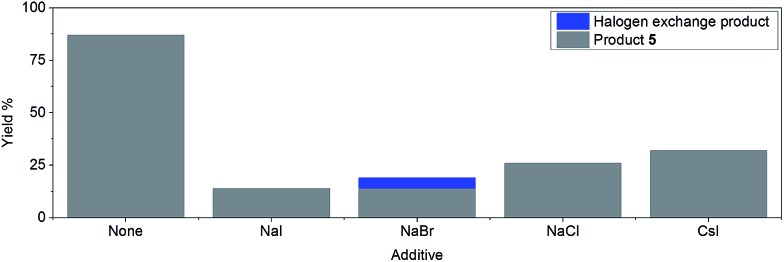
Effect of halide salts (1 eq.) on the reaction yield after 14 hours of reaction time.

However, the inclusion of 1 eq. of halide salt, *e.g.* NaCl, NaI, NaBr, and CsI, all led to significant decreases in conversion after 14 hours, with small amounts of halogen exchange products (Fig. 2).^42^ This inhibition may be explained by a reversible oxidative addition/atom transfer/SET,^42^ which leads to a slower overall reaction rate (through the reduction of the concentration of the active catalyst)/lower conversion. Alternatively, binding of the halide to the active catalyst may also result in a less reactive catalytic species. The observed conversion order is NaCl > NaBr > NaI, consistent with the order of increasing solubility from NaCl to NaI in DMF.^51^ However, inclusion of 1 eq. of CsI gave a higher conversion than both NaI and NaCl, suggesting that the solubility of the halide salt may not be the only important factor.

**Fig. 3:**
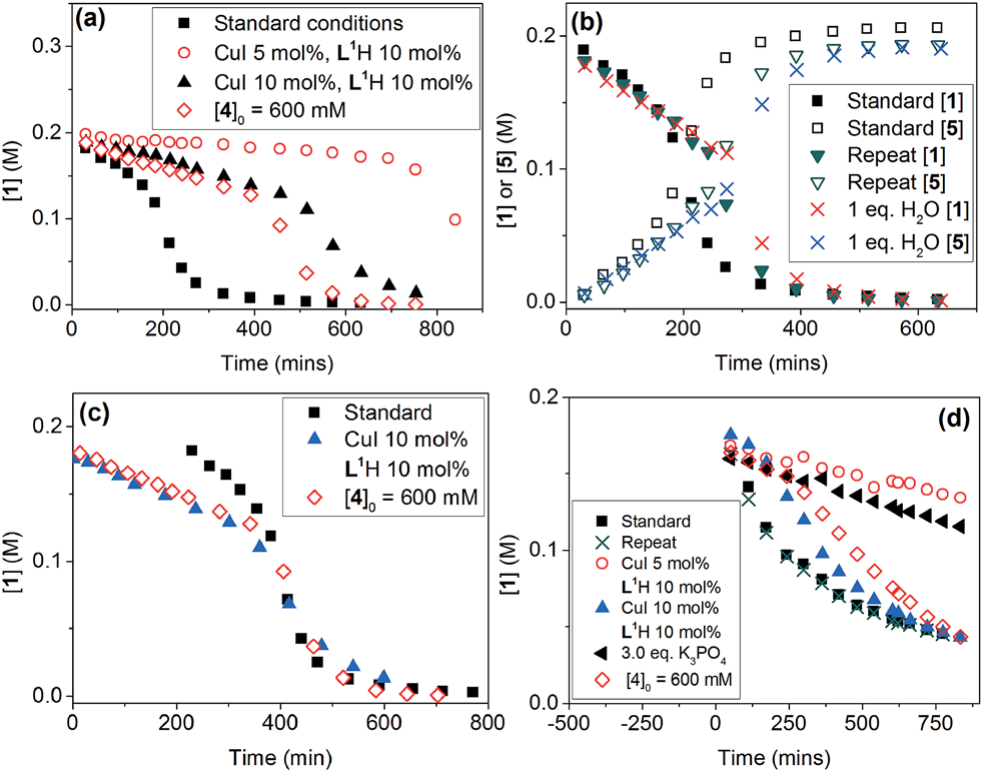
Kinetic data for the reactions from **1** and **4** to **5**. Standard conditions (■): [**1**]_0_ = 200.0 mM, [**4**]_0_ = 300.0 mM, [**L^1^**H]_0_ = 40.0 mM, [CuI]_0_ = 20.0 mM and 1.5 eq. Cs_2_CO_3_ in DMF. (a) Reaction profiles of [**1**] *vs.* time using **L^1^**H/Cs_2_CO_3_; (b) [**1**] and [**5**] *vs.* time under standard conditions, the repeated reaction under standard conditions, and the reaction in the presence of 1 eq. of H_2_O; (c) superimposed reaction profiles by matching the reaction time at 50% conversion; and (d) reaction profiles of [**1**] *vs.* time using **L^2^**H/K_3_PO_4_.

In spite of these, the most significant differences to the homogeneous reactions above are the induction periods in the reactions using **L^1^**H/Cs_2_CO_3_ and **L^2^**H/K_3_PO_4_ (Fig. 3a and d). The reaction using **L^3^**/Cs_2_CO_3_ was very slow, reaching only 57% conversion after 48 hours (see ESI, Fig. S27†) when milled Cs_2_CO_3_ (*vide infra*) was employed. Buchwald and co-workers alluded to a short ‘time-lapse’ at the beginning of a reaction using a *trans*-1,2-diaminocyclohexane/K_3_PO_4_ combination. This was attributed to a variation in trace moisture, and not further investigated.^23^ In this study, an otherwise identical reaction performed in the presence of 1.0 eq. of water showed little change to the induction period (Fig. 3b). Various anecdotal accounts from academia and industry on the poor reproducibility of the Ullmann–Goldberg reactions suggested that this induction period may be one of the sources of such an issue.^38^ Thus, further investigation was carried out to investigate the origin of this induction period.

A small variation (∼50 minutes) in the length of the induction period was detected between supposedly identical runs in the same batch of reactions (Fig. 3b). Given that the same batches of dried Cs_2_CO_3_ and other chemicals stored in a glovebox were used for all these experiments, this ruled out a link between the variations in induction time and the moisture content of the inorganic base. Lowering [CuI]_0_ and both [CuI]_0_ and [**L^1^**]_0_, and increasing [**4**], all led to much more significant increases in the length of the induction period and a change from a 6 hour reaction time (to reach >95% conversion) under standard conditions to >17, 15 and 12 hours, respectively. These variations were mainly due to increases in the induction time. Shifting the kinetic profiles in Fig. 3a to match their 50% conversion points showed little difference in the kinetic profiles after 50% conversion for the three reactions (Fig. 3c). Nevertheless, the reaction with [**L^1^**] = 20.0 mM (10 mol%) showed a slower rate after induction, indicating that the ligand has a more prominent role under these conditions, compared to in the homogeneous reaction above. The observed dependence of the induction time on [CuI], [**L^1^**] and [**4**] in Fig. 3a clearly indicates that this behaviour is catalytically relevant. The same kinetics, albeit with a less pronounced induction, were observed with the K_3_PO_4_/**L^2^** catalytic system (Fig. 3d).

Induction kinetics in catalytic systems are often explained by the slow conversion of a pre-catalyst to the active catalyst or by the requirement of pre-mixing of key components.^52^ A possible explanation in our reaction may be the rapid and significant formation of a Cu(ii) complex at the beginning of the reaction, which is slowly reduced under the reaction conditions to generate the active Cu(i) catalyst.^53^,^54^ A recent study by McGowan *et al.* also reported a Cu(ii) pre-catalyst in Ullmann–Goldberg coupling with phenols.^55^ This hypothesis is supported by our observation of a rapid colour change of the solution from colourless to deep dark blue upon the introduction of CuI into the reaction mixture. This colour gradually dissipated to give a colourless solution as the reaction progressed. To evaluate this hypothesis, an *in situ* EPR experiment was performed under turnover conditions. The time-resolved EPR data showed an immediate formation of a Cu(ii) species upon addition of CuI to the reaction mixture at room temperature (see ESI, Fig. S29†). Upon heating, this signal disappeared within 5 minutes and no EPR signal was detected for the solution phase throughout the rest of the reaction. A similar Cu(ii) EPR signal was also immediately generated by simply treating ultrapure CuI (99.999% purity) with **L^1^**H and anhydrous, degassed DMF. Thus, while some Cu(ii) species were formed at the beginning of the reaction, their short lifetimes mean that they are not responsible for the observed two-stage kinetics.

Consequently, the observed semi-reproducible induction time can only be attributed to the biphasic nature of the reaction. The solid form of the Cs_2_CO_3_ base and its link with the induction period were subjected to detailed investigation. The reaction using **L^1^**H/Cs_2_CO_3_ was chosen over the reaction using **L^2^**H/K_3_PO_4_ due to the more pronounced induction period in this reaction.

### The effect of Cs_2_CO_3_ solids on the reaction kinetics

**Fig. 4:**
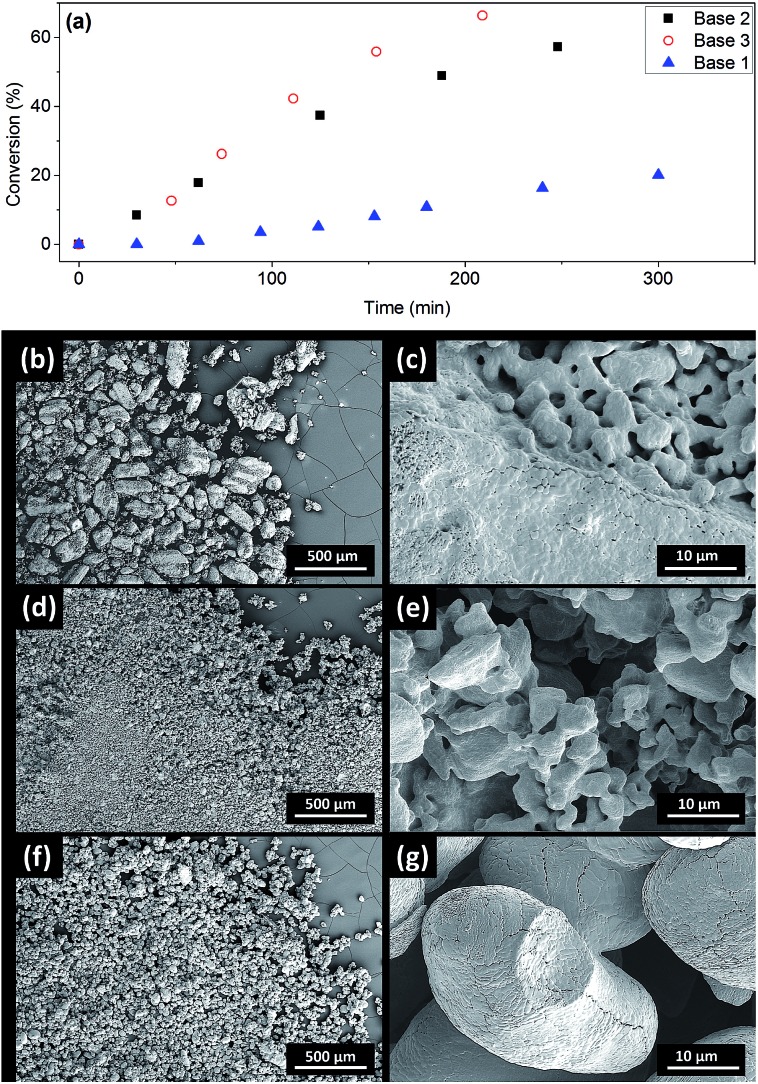
(a) Kinetic data for the reaction from **1** and **4** to **5**, showing conversion *vs.* time under standard conditions using different batches of Cs_2_CO_3_; (b and c) SEM images of Base 2 at different magnifications; (d and e) SEM images of Base 1 at different magnifications; and (f and g) SEM images of Base 3 at different magnifications.

The induction periods above were observed with milled Cs_2_CO_3_ (99%, supplied by Chemetall, Base 1, see ESI† for further specifications). However, no induction was observed for Cs_2_CO_3_ supplied by Sigma-Aldrich (99%, Base 2) or Acros Organic (99.5%, Base 3), resulting in much higher initial rates (Fig. 4a). Base 3 gave the fastest reaction. Thus, the three batches of Cs_2_CO_3_ were characterized using SEM. The milled Cs_2_CO_3_ from Chemetall has a smaller particle size and narrower distribution than Base 2 from Sigma Aldrich, but they both have a similar morphology (Fig. 4b and d). Base 3 has a larger and more evenly distributed particle size and a lower surface area due to its non-porous morphology.

Developing metal catalysed coupling reactions often includes tuning the amount of inorganic base for optimal conversion and selectivity. The amount of base usually reported for the Ullmann–Goldberg reaction is 1.5–4.0 equivalents depending on the ligand, catalyst precursor, solvent and substrate.^14^ Yet the effects of the solid form of the base and its fate have not been investigated in this context. Reliable information on the solubility of these bases in organic solvents at elevated temperatures is extremely difficult to find in the literature.

To probe the link between the morphologies and surface areas of the bases and their kinetic behaviour, solid samples were recovered from the reaction mixtures using Base 1 and Base 2 at 2.5 and 5.0 hours and characterised by SEM, Energy-Dispersive X-ray Spectroscopy and powder X-ray diffraction (Fig. S34–S37†). As the reaction progresses, CsI accumulates on the solids and the soft-edge structure of Cs_2_CO_3_ (Fig. 4d) gives way to crystalline CsHCO_3_. No diffraction signal for Cs_2_CO_3_ was detected at the end of the reaction. The similar images of Base 1 and Base 2 at 2.5 and 5 hours show that they both change toward a common final state as the reaction progresses. The difference in the initial reaction rates must therefore originate from their initial states. We hypothesised that the difference in surface area, and hence their rates of dissolution in DMF,^56^ is the main reason for the observed induction period with Base 1.

Compton *et al.* reported major influences of the particle size, shape and temperature on the rate of dissolution of carbonates in DMF, further reinforcing the importance of characterisation of the inorganic bases in catalytic reactions.^56^ Common practice in catalysis favours a finely milled inorganic base which improves the rate of the reaction. However, our results showed that the milled Base 1 resulted in a slow induction. This counter-intuitive relationship may be explained by the faster rate of base release into solution, leading to a larger extent of deprotonation when Base 1 is employed due to its high surface area. This better availability of the base can lead to a more deprotonated pyrrolidinone, pushing equilibrium A to the left (Scheme 1). Thus, the majority of the Cu(i) catalyst becomes the inactive Cu-bisamido species. As the reaction progresses, the formation of CsI and CsHCO_3_ on the solid surface slowly restricts the availability of Cs_2_CO_3_, ultimately reducing the amount of deprotonated pyrrolidinone and pushing equilibrium A to the right, releasing the active catalyst. This puts an end to the slow stage of the reaction. However, quantification of the soluble Cs_2_CO_3_ in the reaction mixture using Atomic Absorption Spectroscopy with a Cs hollow-cathode lamp was prevented by the much higher solubility of the CsI by-product in DMF at 90 °C (Fig. S42, ESI†).

### Reaction without a base

**Scheme 4:**
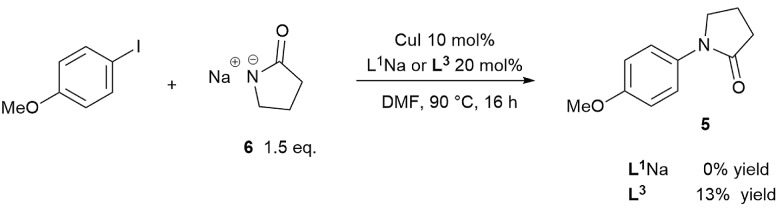
Reaction scheme for the base-free cross coupling using the Na salt of 2-pyrrolidinone.

To prove/disprove our hypothesis on the effect of over-deprotonation on Ullmann–Goldberg coupling, a reaction without base was devised. The removal of the inorganic base enables the direct evaluation of the reaction rate without the mass transfer complication from the solid phase. In order to circumvent the required deprotonation of ligand **L^1^**H, its sodium salt **L^1^**Na was employed instead. Thus, the reaction between the sodium salt **6** and 4-iodoanisole was carried out using CuI/**L^1^**Na and CuI/**L^3^** catalysts (Scheme 4). Whilst the use of a deprotonated nucleophile has been known to work in the copper-catalysed etherification of aryl halides,^57^ no prior example was found for Cu(i)-catalysed C–N coupling reactions.

The reactions using 1.5 equivalents of **6** gave no conversion to the cross-coupling product when using **L^1^**Na and just 13% conversion over 16 hours with **L^3^**. In the case of the CuI/**L^3^** catalyst, the characteristic deep-red colour, observed under turnover conditions as a result of coordination of the ligand **L^3^**, was not observed.^25^,^58^ Instead a colourless solution formed, indicating that the inactive complex **II** was the major species. ESI-MS experiments collaborated this, showing that when 1 eq. of **6** was added to a mixture of CuI, **L^3^** and Cs_2_CO_3_ in DMF, the signal of [Cu(**L^3^**)_2_]^+^ quickly dissipated and was replaced with a very strong signal of the free **L^3^** ligand as the pyrrolidinate displaced it (see ESI, Fig. S39†).

**Fig. 5:**
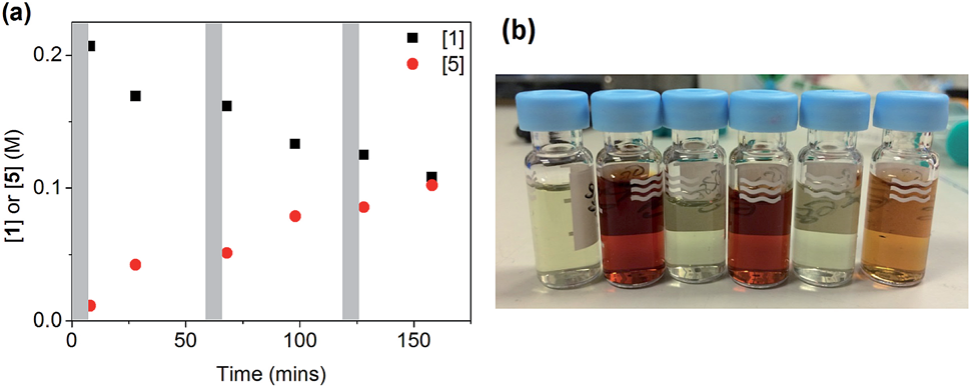
(a) Reaction profile for the reaction in Scheme 4 ([**1**]_0_ = 200 mM) with portion-wise addition of salt **6**. Each grey area represents a 5 minute addition of 0.5 eq. of **6**. (b) The colour change from deep-red to colourless after each 0.5 eq. addition of **6** in (a).

When salt **6** was added in three portions of 0.5 equivalents (as solutions in DMF) to a reaction using CuI/**L^3^**, a very different result was obtained, as shown in Fig. 5. After each addition, a fast reaction was observed concurrent with a change in the solution colour from deep-red to colourless. As the reaction slowed down, the colour returned to deep red until the next addition. A total conversion of ∼50% was obtained after 160 min, in contrast to the 13% conversion when all 1.5 equivalents of **6** were added at the beginning of the reaction. The successive smaller changes in conversion after each portion of **6** can be attributed to the inhibitive effect of the by-product NaI on the reaction, which we have observed above.

These results are consistent with the hypothesis that a high concentration/availability of base or deprotonated amide suppresses catalytic turnover. This inhibitive effect is due to equilibrium A (Scheme 1), which is sensitive to the amount and the basicity of the base, and ultimately the competitive binding between ancillary ligands and the amine/amide substrate with the Cu(i) cation.

## Conclusions

The reported studies above highlighted various pathways for catalyst deactivation/inhibition in Ullmann–Goldberg coupling reactions: (i) product inhibition with amine products, (ii) by-product inhibition with inorganic halide salts, and (iii) ligand exchange by a soluble carboxylate base, leading to significantly more side products compared to reactions using inorganic bases. These issues came from the relative binding constants between common ancillary ligands, compared to other species in the reaction, and the Cu(i) species.^59^–^63^ Competitive binding with other possible ligands in the reaction mixture, including the reaction product and coupling partner, is responsible for the observed reversible deactivation of the catalysts, *e.g.* (i) and (iii). Improvements in ligand design for these reactions must achieve better catalyst stability, although maintaining catalytic activity can be challenging due to the limited coordination number and small radius of the Cu(i) cation.

Furthermore, the physical characteristics of the inorganic bases were found to have marked effect on the mechanism of the reaction. More finely milled Cs_2_CO_3_ with a higher surface area led to a somewhat variable induction period, instead of an increase in reaction rate. This is due to equilibrium A and explains the anecdotal poor reproducibility of these reactions. The combined effect of the neutral/anionic bases and the polarity of the solvent in a palladium catalysed C–N coupling reaction has been investigated by Norrby,^64^ but this study is the first which establishes the link between the solid form of the inorganic base and the reaction kinetics and mechanism. Given the widespread use of inorganic and partially soluble salts as bases in metal catalysed reactions and recent computational studies showing direct interaction between the carbonate anion, caesium cation and catalytic species,^65^,^66^ some of which may explain the benefits of caesium bases in improving conversion and decreasing side products in catalytic reactions, these results suggest that the solid form of the inorganic bases and the rate of base release should be considered as important variables in many other reactions.

## Conflicts of interest

There are no conflicts to declare.

## Supplementary Material

Supplementary information
