# Supplementary material for: Origins of high catalyst loading in copper(i)-catalysed Ullmann–Goldberg C–N coupling reactions
Source: Chem Sci. 2017 Aug 29;8(10):7203–10. doi: 10.1039/c7sc02859h (PMC5688446; doi:10.1039/c7sc02859h)
Supplement: Supplementary file 1 [file SC-008-C7SC02859H-s001.pdf]

Electronic Supplementary Material (ESI) for Chemical Science.
This journal is © The Royal Society of Chemistry 2017

Origins of High Catalyst Loading in Copper(I)-Catalyzed Ullmann-Goldberg C-N Coupling Reactions

*Grant J. Sherborne, Sven Adomeit, Robert Menzel, Jabor Rabeah, Angelika Brückner, Mark R. Fielding, Charlotte E. Willans, Bao N. Nguyen**

1. General methods and materials 4
1.1 Reagents and materials 4
1.2 General analytical data 4
1.3 Base suppliers, purity, and drying protocols 4
2. Synthesis of organic compounds 4
2.1 Tetrabutylammonium adipate (TBAA) 4
2.2 *N,N*-Dimethylglycine 5
2.3 Synthesis of sodium *N,N*-dimethylglycinate 5
2.4 Synthesis of sodium pyrolidinide 6
2.5 Synthesis of 1-(4-methoxyphenyl)-piperidine 6
2.6 Synthesis of 1-(4-methoxyphenyl)pyrrolidin-2-one 7
3. NMR spectra of organic compounds 8
3.1 1-(4-Methoxyphenyl)pyrrolidin-2-one 8
3.2 1-(4-Methoxyphenyl)-piperidine 9
3.3 *N,N*-Dimethylglycine 10
3.4 Tetrabutylammonium adipate 11
3.5 4-Methoxyphenol 11
4. *In situ* ^{1}H NMR kinetic study 12
4.1 General procedure for NMR experiments 12
4.2 Procedure for obtaining kinetic data 12
4.3 Reaction conditions for *in situ* study 12
4.4 Characterisation of products (d_7-DMF) 13
4.5 Kinetic data in d_7-DMF 21
4.6 Kinetic data in d_3-MeCN 31
5. Kinetic experiments using inorganic bases 37
5.1 General procedure for reactions with automated sampling 37
5.2 HPLC calibration 38
5.3 Kinetic data with L^1H/Cs_2CO_3 39
5.4 Kinetic data with L^2H/K_3PO_4 46
5.5 Reaction with L^3/Cs_2CO_3 53
6. Reaction with additional halide salts 54
7. EPR experiments 54
7.1 *In situ* EPR experiment 54
7.2 $CuI/L^1H/DMF$ experiment 55
8. Characterization of commercial Cs_2CO_3 samples 56

8.1 Sample preparation 56
8.2 SEM/EDX experiments 56
8.3 Surface area measurements 60
9. Characterisation of solid during and after reaction 60
9.1 SEM/EDX experiments 60
9.2 XRD measurements 63
10. Base-less coupling reaction 63
10.1 GC Calibrations 63
10.2 Reaction Procedures 65
11. Base Solubility by ^{133}Cs NMR 66
12. References 67

1. General methods and materials

1.1 Reagents and materials

Unless otherwise stated, all reactions were performed under an atmosphere of nitrogen, using flame-dried glassware. Reactions were set up using air-sensitive techniques on a Schlenk manifold or in a nitrogen-filled glovebox. Anhydrous solvents were dried by passing the solvent over activated alumina via the Dow-Grubbs solvent system (Pure Solv^{TM}) unless otherwise specified. Anhydrous *N,N*-dimethylformamide and acetonitrile (99.8%) were purchased from Sigma Aldrich. Reaction solvents were degassed by bubbling of nitrogen through for a minimum of 30 minutes. Anhydrous NMR solvents for air sensitive products were degassed using three freeze-thaw cycles. All other reagents were obtained from commercial suppliers and used without further purification.

1.2 General analytical data

^{1}H and ^{13}C NMR data were performed using deuterated chloroform ($CDCl_3$), unless otherwise stated and recorded on either an Avance 300 (Brüker Biospin GmbH), or Avance 500 (Brüker Biospin GmbH). All kinetic data was obtained on the Avance 500 and ^{133}Cs NMR was run on an Avance 400 or 500 MHz spectrometer. Microanalysis data was obtained by Tanya Marinko-Covell of the University of Leeds. Hydrogenation of amides was performed using a 300 mL Parr Pressure Reactor. High resolution mass spectra were collected on a Brüker Daltonics (microTOF) instrument operating in the electrospray mode. GC/MS data was obtained using Agilent HP2890 series GC system, with an Agilent HP5973 mass selective detector on EI mode. Column chromatography was performed using Geduran© Si 60 silica gel with the stated solvents. FT-IR spectroscopy measurements were taken on a Brüker Alpha Platinum-ATR. HPLC data was collected from an Agilent 1200 HPLC system using a 30 x 4.6 mm Waters Acquity BEH C18 1.7 µm column and UV/Vis detector set at 285 nm. GC data was obtained from an Agilent Technologies 7890B gas chromatograph using an Agilent J&W HP-5 GC Column, 30 m, 0.32 mm, 0.25 µm.

1.3 Base suppliers, purity, and drying protocols

Cesium carbonate was obtained from three individual sources; Sigma Aldrich (99 %, Lot # BCBP3311V), Acros Organics (99.5 %, Lot # A0359643) and Chemetall (Milled, D_{50} = 20 µm, D_{90} = 50 µm, Bx # 27091B032) and oven dried at 80 °C before being used. Prior to SEM analysis, all bases were dried at 75 °C for a period of at least 24 hours, ensuring consistency between samples.

2. Synthesis of organic compounds

2.1 Tetrabutylammonium adipate (TBAA)

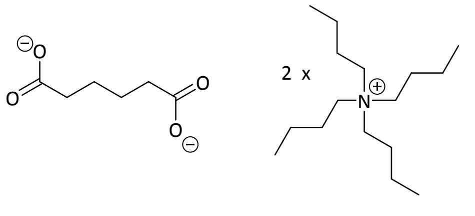

Tetrabutylammonium adipate was prepared based on literature procedures.[1] Tetrabutylammonium hydroxide (1M solution in MeOH, 3.4 mL, 3.42 mmol) was added with adipic acid (250 mg, 1.71 mmol) in a round bottomed flask with activated 4 Å molecular sieves. The reaction was stirred under an atmosphere of nitrogen at room temperature for 18 hours and upon completion, the solution was filtered over a short pad of

Celite with 50 mL MeOH. The majority of the solvent was removed using a rotary evaporator and the resulting thick slurry dried on a Schlenk line to give a white solid. The white solid was dried further under vacuum for 9 hours and stored under a nitrogen atmosphere without further purification.

^{1}H NMR (500 MHz, D_2O) δ = 3.21 (t, J = 9.0 Hz, 16H), 2.20 (m, 4H), 1.66 (m, 16H), 1.56 (m, 4H), 1.39 (dt, J = 21.2, 7.3 Hz, 16H), 0.93 (t, J = 7.3 Hz, 24H).

Literature Values:[1] 1H NMR (300 MHz, D_2O): δ 3.12 (t, J = 9.1 Hz, 16H), 2.11 (m, 4H), 1.54 (m, 16H), 1.43 (m, 4H), 1.27 (dt, J = 21.6, 7.2 Hz,16H), 0.89 (t, J = 7.2 Hz, 24H).

2.2 *N,N*-Dimethylglycine

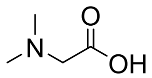

Synthesis was performed as outlined by Makriyannis *et al.*[2] A mixture of glycine (10.0 g 0.133 mmol), formaldehyde (37% w/v in H_2O, 32.0 mL, 0.361 mmol) and palladised charcoal (10% w/w Pd, 4.0 g) was stirred in ethanol (150 mL) under 3 bar H_2 using a Parr pressure reactor for 12 hours. The resulting mixture was filtered over Celite with ethanol and evaporated to dryness. The product was crystallised from ethanol/diethyl ether and dried *in vacuo*, yielding of *N,N*-dimethylglycine as a white solid (11.8 g, 86%).

^{1}H NMR (500 MHz, D_2O) δ = 3.73 (s, 2H), 2.94 (s, 6H); ^{13}C NMR (125 MHz, D_2O) δ = 60.0 (C-2), 43.7 (C-1); vmax (neat)/cm^{-1} 3386, 3025, 1607 (C=O), 1470, 1391, 1356; HRMS (ESI) *m/z* [M+Na]$^+$: Calcd for $C_4H_9NNaO_2$: 126.0531. Found: 126.0525.

Literature values:[2] ^{1}H NMR (D_2O) δ = 3.96 (s, 2 H), 2.80 (s, 6H)

2.3 Synthesis of sodium *N,N*-dimethylglycinate

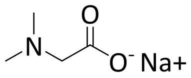

NaH (60 % dispersion in mineral oil, 77.6 mg, 1.94 mmol) was weighed out into a flame-dried round bottom flask kept under nitrogen. The NaH was washed with three 6 ml batches of degassed pentane and the pentane removed via syringe. 2.5 ml of anhydrous THF was added at the NaH stirred as a suspension before being cooled down to 0 °C. *N,N*-dimethylglycine (200 mg, 1.94 mmol) was suspended in 2.5 ml MeCN and added slowly to the NaH, the reaction was stirred at 0 °C until no further bubbles were seen to evolve. The colourless solution was allowed to heat to room temperature and stirred for 30 minutes further before the solvent was removed *in vacuo*. The off-white solid was washed three times with degassed MeCN, giving 80 mg (33 % yield) of an insoluble white solid was obtained and stored in an N_2 filled glovebox. ^{1}H NMR spectroscopy in d_3-MeCN showed no starting material peaks, indicating all **L^1**H had reacted. Further characterization of this salt was unsuccessful due to its very low solubility in organic solvents.

2.4 Synthesis of sodium pyrolidinide

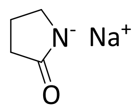

NaH (60 % dispersion in mineral oil, 1.048 g, 26.2 mmol) was weighed out into a flame-dried round bottom flask kept under nitrogen. The NaH was washed with three 10 ml batches of degassed pentane and the pentane removed via syringe. 20 ml of anhydrous THF was added at the NaH stirred as a suspension before being cooled down to 0 °C. 2-Pyrrolidinone (2.0 ml, 26.2 mmol) was added dropwise and the reaction stirred at 0 °C until no further bubbles were observed to evolve. The colourless solution was allowed to warm up to room temperature and stirred for 30 minutes further before the solvent was removed *in vacuo*. 2.35 g (84 % yield) of an insoluble white solid was obtained and stored in an N_2 filled glovebox. ^{1}H NMR spectroscopy in d_3-MeCN showed no starting material peaks, indicating all 2-pyrrolidinone had reacted.

2.5 Synthesis of 1-(4-methoxyphenyl)-piperidine

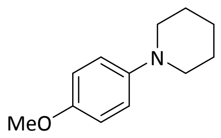

CuI (19.0 mg, 0.1 mmol), *N,N*-dimethylglycine (20.8 mg, 0.2 mmol), 4-iodoanisole (236.0 mg, 1.0 mmol) and tetrabutylammonium adipate (942.0 mg, 1.5 mmol) were added to a flame dried Schlenk flask. The vessel was evacuated and backfilled with nitrogen three times before piperidine (158.0 µL, 1.5 mmol) and 4 mL of degassed anhydrous DMF were added by syringe. The Schlenk flask was sealed and the reaction stirred at 90 ºC for 71 hours. Upon completion, the reaction mixture was filtered over a short pad of silica with ethyl acetate and evaporated to give the organic compounds in DMF. The resulting solution was dilute with 50 mL water and extracted with 4 x 20 mL of diethyl ether, with the organic layers combined and evaporated to dryness to give the crude product. The crude product was purified by column chromatography, eluting with a 2:1 mixture of petroleum ether and diethyl ether (R_f = 0.82) to yield 1-(4-methoxyphenyl)piperidine as a yellow oil (73 mg, 38 % yield).

^{1}H NMR (500 MHz, $CDCl_3$) δ = 6.92 (d, *J* = 9.0 Hz, 2H), 6.83 (d, *J* = 9.1 Hz, 2H), 3.77 (s, 3H), 3.02 (t, *J* = 5.4 Hz, 4H), 1.72 (p, 5.5 Hz, 4H), 1.54 (p, 6.0 Hz, 4H); ^{13}C NMR (125 MHz $CDCl_3$) δ = 153.5, 146.9, 118.8, 114.4, 55.6, 52.3, 26.1, 24.2; HRMS (ESI) *m/z* $[M+H]^+$: Calcd for $C_{12}H_{18}NO$: 192.1388. Found: 192.1394.

Literature values:[3] ^{1}H NMR (600 MHz, $CDCl_3$) δ 6.91 (d, *J* = 9.0 Hz, 2H), 6.82 (d, *J* = 9.0 Hz, 2H), 3.76 (s, 3H), 3.02 (t, *J* = 5.5 Hz, 4H), 1.72 (p, *J* = 5.7 Hz, 4H), 1.54 (p, *J* = 6.1 Hz, 2H); ^{13}C NMR (150 MHz, $CDCl_3$) δ 153.5, 146.9, 118.7, 114.3, 55.5, 52.3, 26.1, 24.2.

2.6 Synthesis of 1-(4-methoxyphenyl)pyrrolidin-2-one

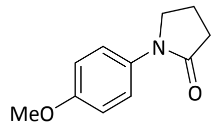

CuI (19.0 mg, 0.1 mmol), *N,N*-dimethylglycine (20.6 mg, 0.2 mmol), Cs_2CO_3 (489.0 mg, 1.5 mmol) and 4-iodoanisole (234.0 mg, 1 mmol) were added to a flame dried Schlenk flask. 3 vacuum purge and nitrogen flush cycles were performed before addition of 2-pyrrolidinone (114 µl, 1.5 mmol) and 5 mL DMF and heated to 90 ^{o}C for 16 hours. The resulting brown liquid was filtered through a short silica plug using 50 mL ethyl acetate and concentrated to give the crude product, which was purified by column chromatography, eluting with a 7 : 3 mixture of *n*-hexane and ethyl acetate (R_f = 0.74) to yield 1-(4-methoxyphenyl)pyrrolidin-2-one as a cream coloured solid (172.9 mg, 90% yield).

^{1}H NMR (500 MHz, $CDCl_3$) δ = 7.49 (d, *J* = 9.2 Hz, 2H), 6.90 (d, *J* = 9.1 Hz, 2H), 3.83 (t, *J* = 7.1 Hz, 2H), 3.80 (s, 3H), 2.58 (t, *J* = 8.0 Hz, 2H), 2.15 (qn, *J* = 7.8 Hz, 2H); ^{13}C NMR (125 MHz $CDCl_3$) δ = 173.9, 156.6, 132.7, 121.9, 114.1, 55.5, 49.2, 32.5, 18.1; νmax (neat)/cm^{-1} 2951, 1883, 1677 (C=O), 1508, 1391, 1223, 1125; Anal. Calcd for $C_{11}H_{13}NO_2$: C, 69.09; H, 6.85; N, 7.32. Found: C, 68.20; H, 6.80; N, 7.20; HRMS (ESI) *m/z* [M+H]$^+$: Calcd for $C_{11}H_{13}NNaO_2$: 214.0844. Found: 214.0845.

Literature values:[4] ^{1}H NMR (400 MHz, CDCl3): δ = 7.49 (d, *J* = 9.1 Hz, 2H), 6.90 (d, *J* = 9.1 Hz, 2H), 3.83 (t, *J* = 7.1 Hz, 2H), 3.80 (s, 3H), 2.59 (t, *J* = 8.1 Hz, 2H), 2.15 (qn, *J* = 7.6 Hz, 2H); ^{13}C NMR (100MHz, CDCl3): δ = 173.9, 156.6, 132.6, 121.9, 114.0, 55.5, 49.2, 32.5, 18.0.

3. NMR spectra of organic compounds

3.1 1-(4-Methoxyphenyl)pyrrolidin-2-one

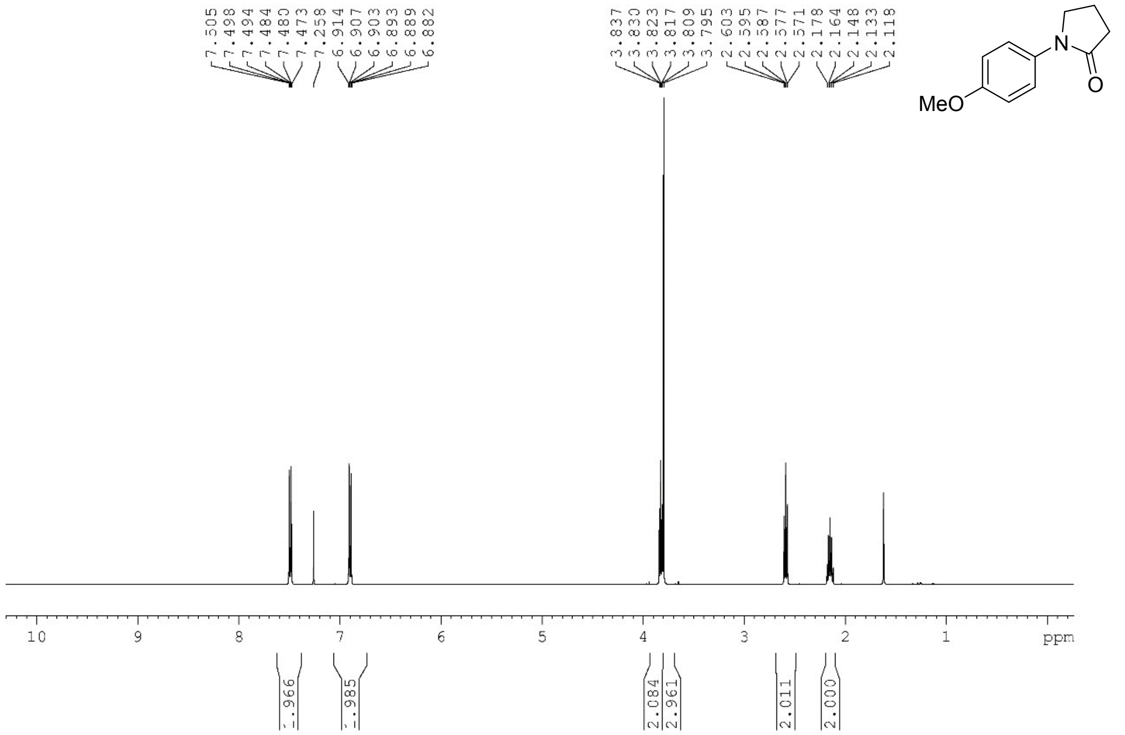

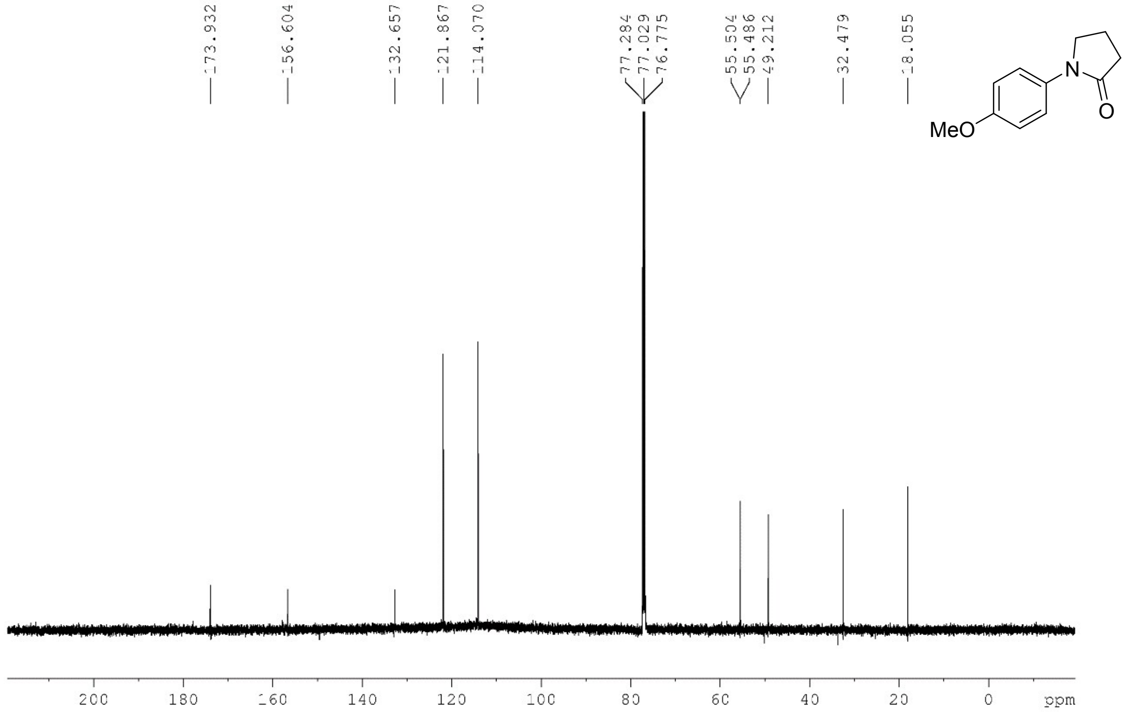

3.2 1-(4-Methoxyphenyl)-piperidine

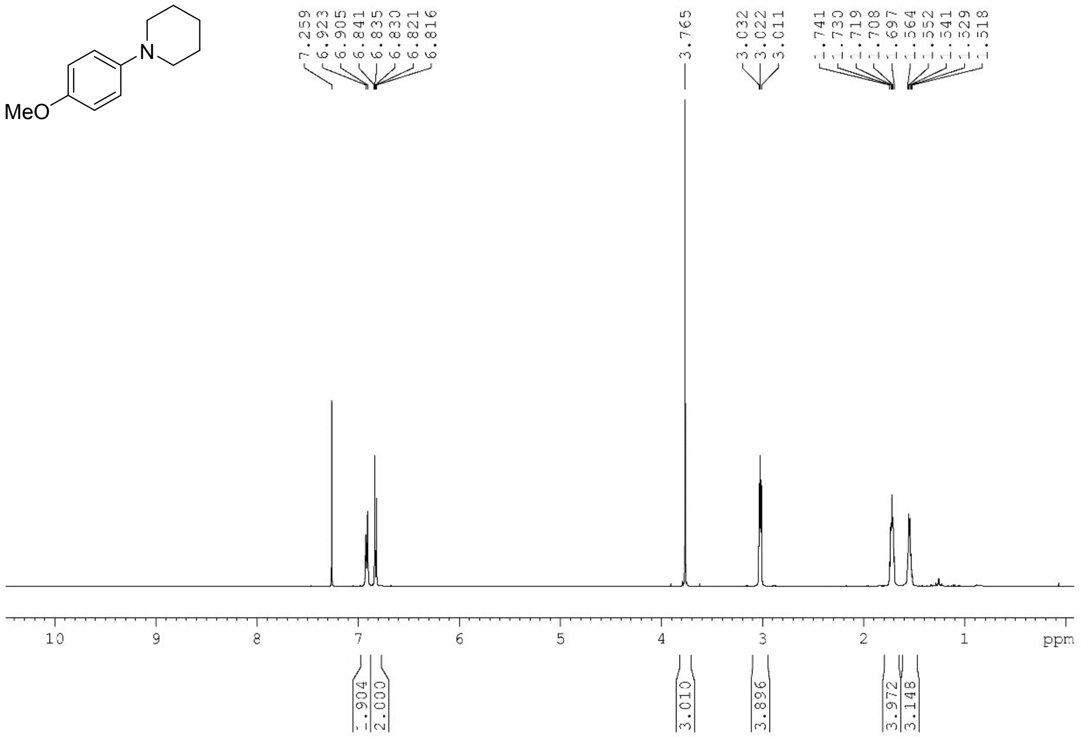

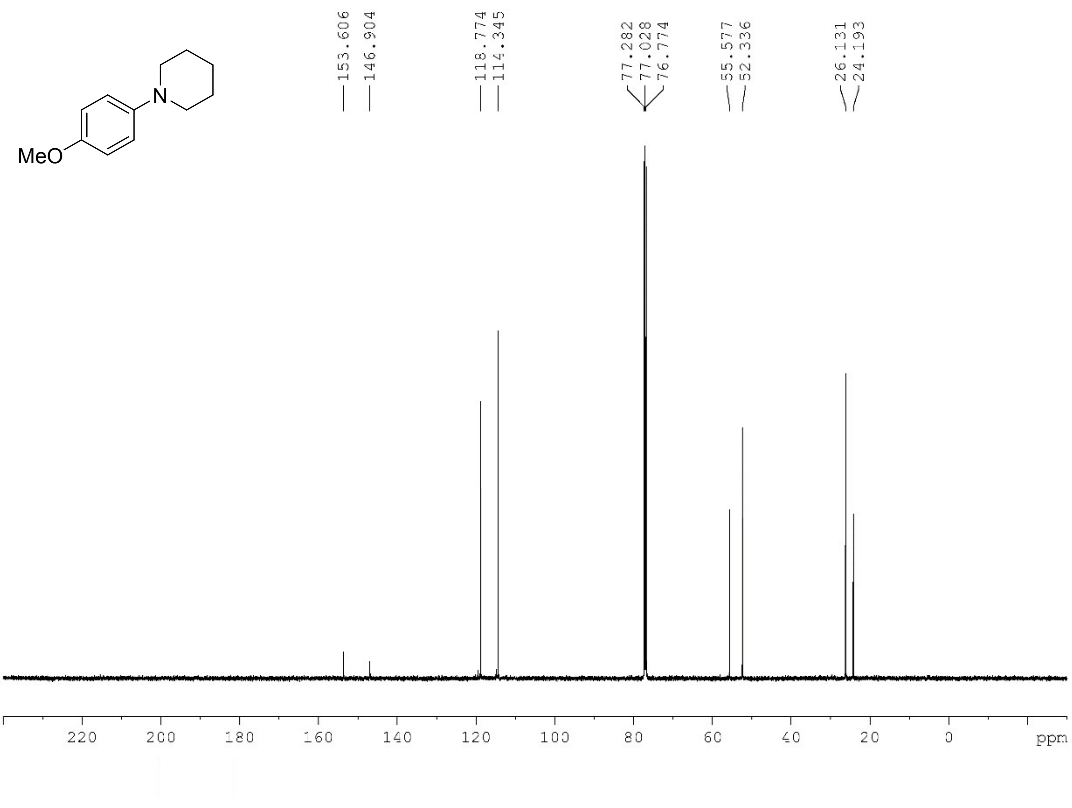

3.3 *N,N*-Dimethylglycine

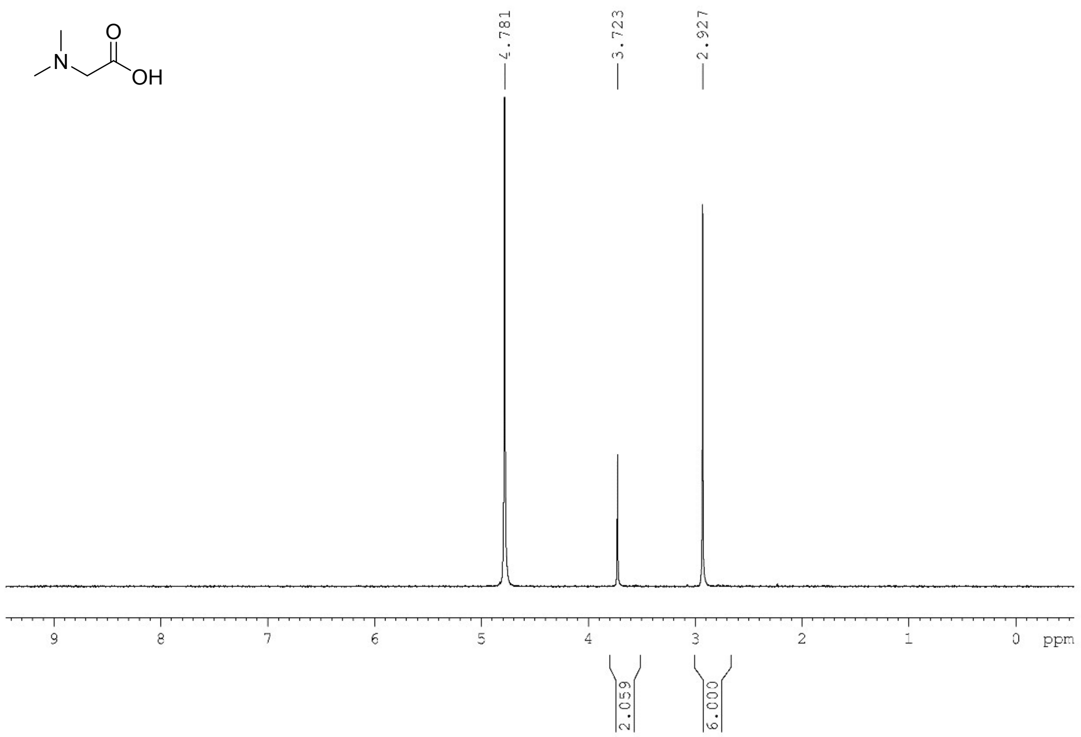

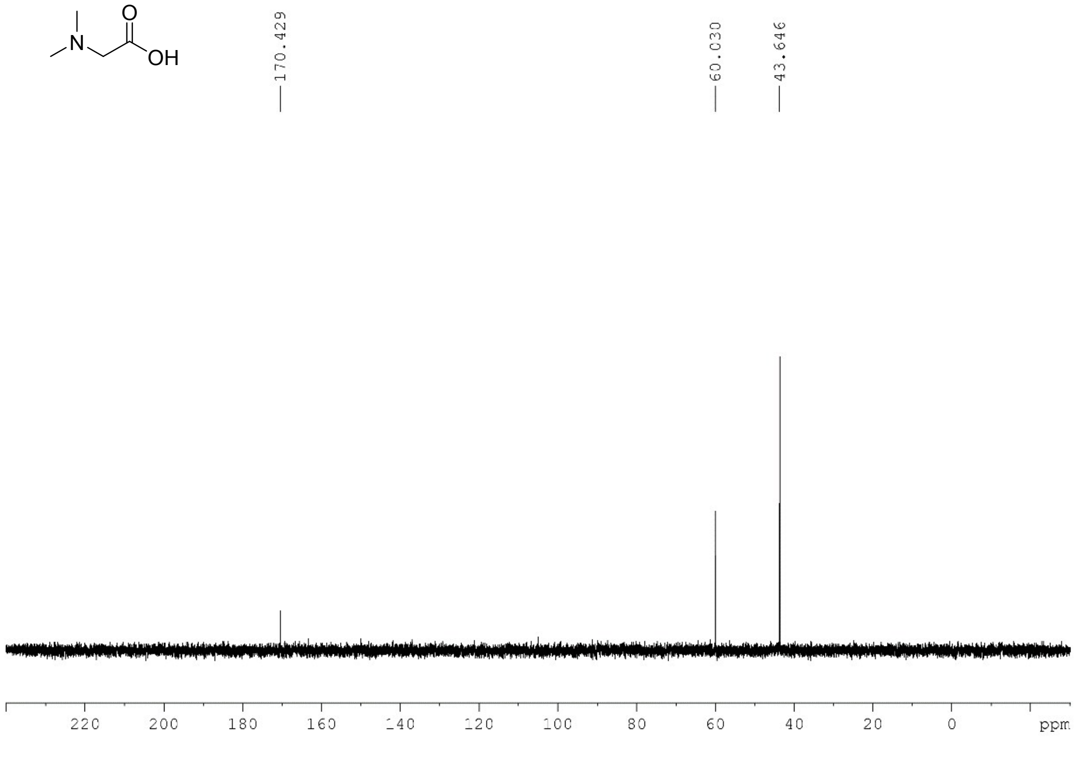

3.4 Tetrabutylammonium adipate

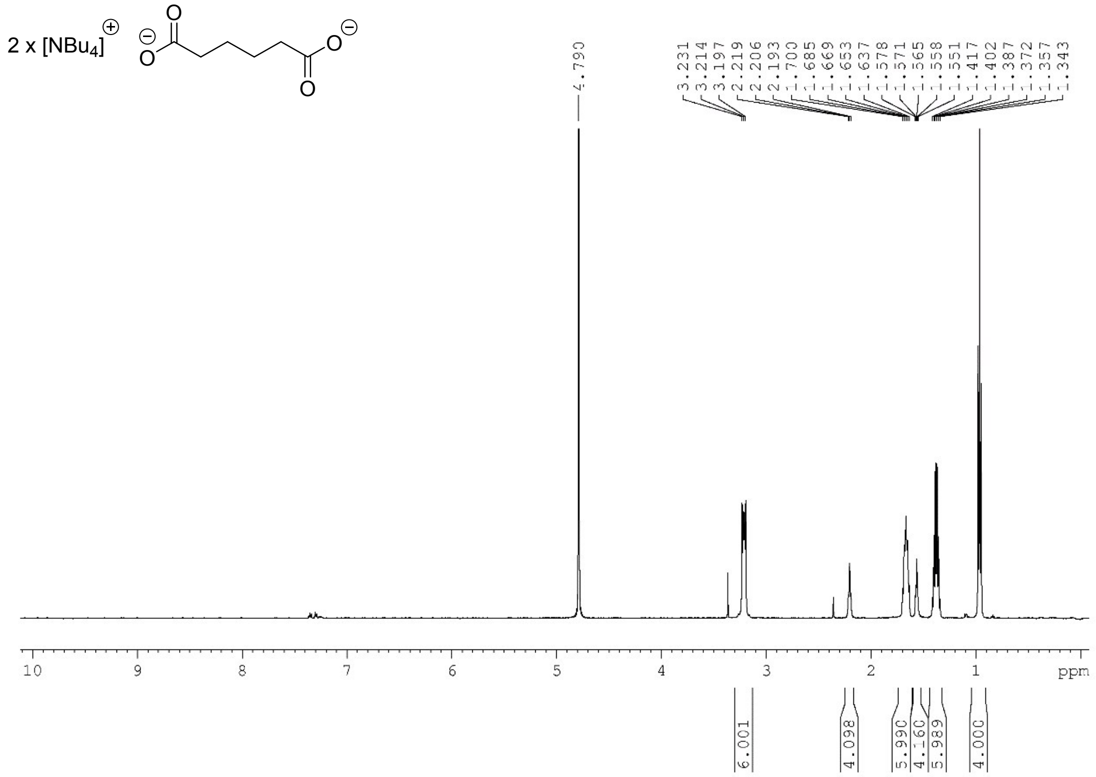

3.5 4-Methoxyphenol

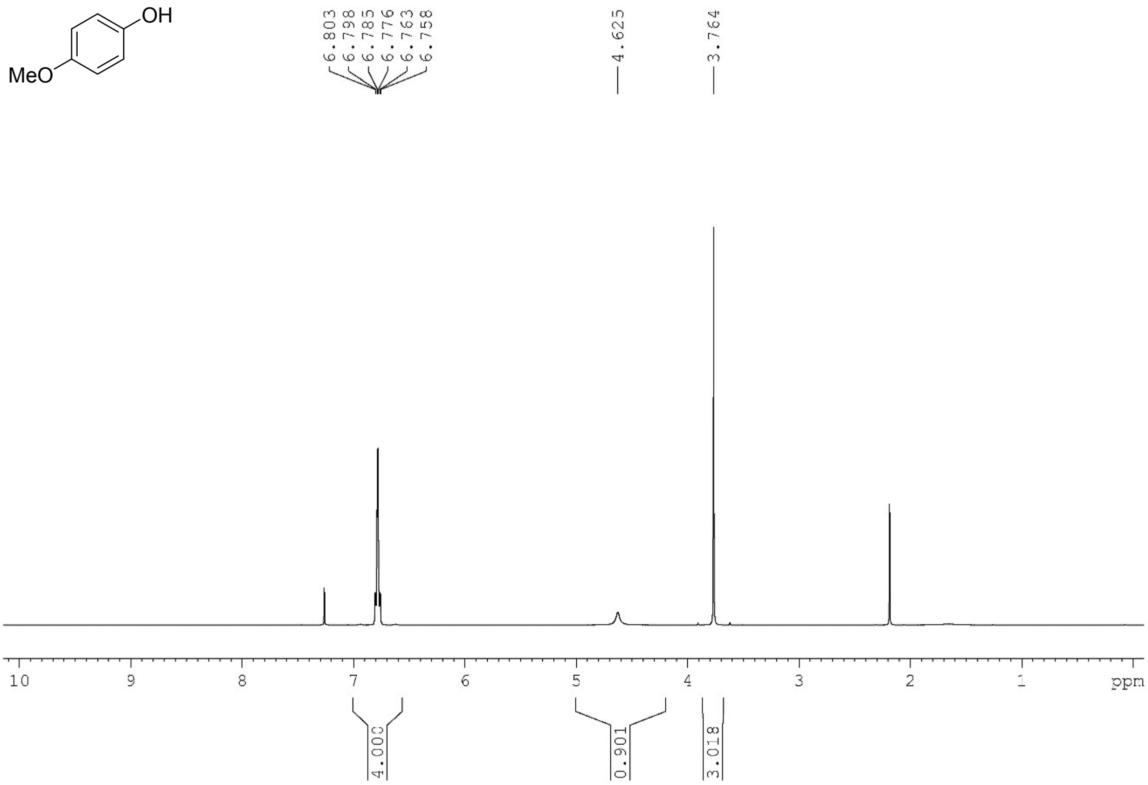

4. *In situ* ^{1}H NMR kinetic study

4.1 General procedure for NMR experiments

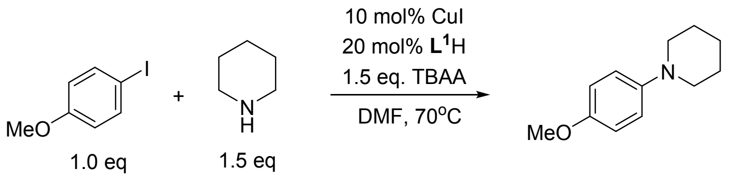

CuI (1.2 mg, 0.0063 mmol) was added with *N,N*-dimethylglycine (1.3 mg, 0.0126 mmol) to a dry NMR tube with a Young valve and taken in to a nitrogen filled glovebox. Inside the glovebox, a solution of TBAA (59.0 mg, 0.0938 mmol) in d_7-DMF (0.25 mL) followed by piperidine (9.0 µL, 0.0911 mmol) were added and the reaction initiated when a solution of 4-iodoanisole (14.6 mg, 0.0624 mmol) in d_7-DMF (0.25 mL) was added. The NMR tube was removed and shaken to ensure homogeneity before the NMR experiment began.

4.2 Procedure for obtaining kinetic data

The NMR sample is prepared as outlined above and is then placed into the NMR machine. A scan is taken at 25 ºC to measure t_0 for the reaction, before the temperature probe is heated to the desired temperature (70 ºC). After this temperature is reached, an NMR spectrum is collected every 10 minutes.

The resulting spectra are processed using the TopSpin™ package and the integrals of all aromatic species (6 – 8 ppm) are normalised to 100. The conversions are noted for each known compound and the concentration of each species calculated based on the starting concentration of aryl iodide. Rate data were generated using differential function in Origin Pro 9.0.

4.3 Reaction conditions for *in situ* study

	MW	Density	Standard	2x [2]	2x [TBAA]	2x [L^{1}H]	Product Inhibition	Same "Excess"	0.5x CuI/L^{1}H
CuI (mg)	190.44		1.2	1.2	1.2	1.2	1.2	1.2	0.6
DMG (mg)	103.12		1.3	1.3	1.3	2.6	1.3	1.3	0.7
TBAA (mg)	629.07		59.0	59.0	118.0	59.0	59.0	39.3	59.0
DMF (µl)			500	500	500	500	500	500	500
4-Iodoanisole (mg)	234.03		14.6	14.6	14.6	14.6	14.6	14.6	14.6
Piperidine (µl)	85.15	0.862	9	18	9	9	9	6	9
Product (mg)	191.27						7.4		

4.4 Characterisation of products (d_7-DMF)

4.4.1 ^{1}H NMR Assignment

An example NMR taken from the kinetic monitoring study is shown below, many of the peaks belong to the tetrabutylammonium cation due to its stoichiometry and large number of protons. The integration of the *in situ* NMR spectra was performed on the aromatic peaks shown zoomed in due to significant overlap between peaks in the methoxy region. Aromatic peaks were assigned from 'spiking' experiments, COSY experiments and reference spectra.

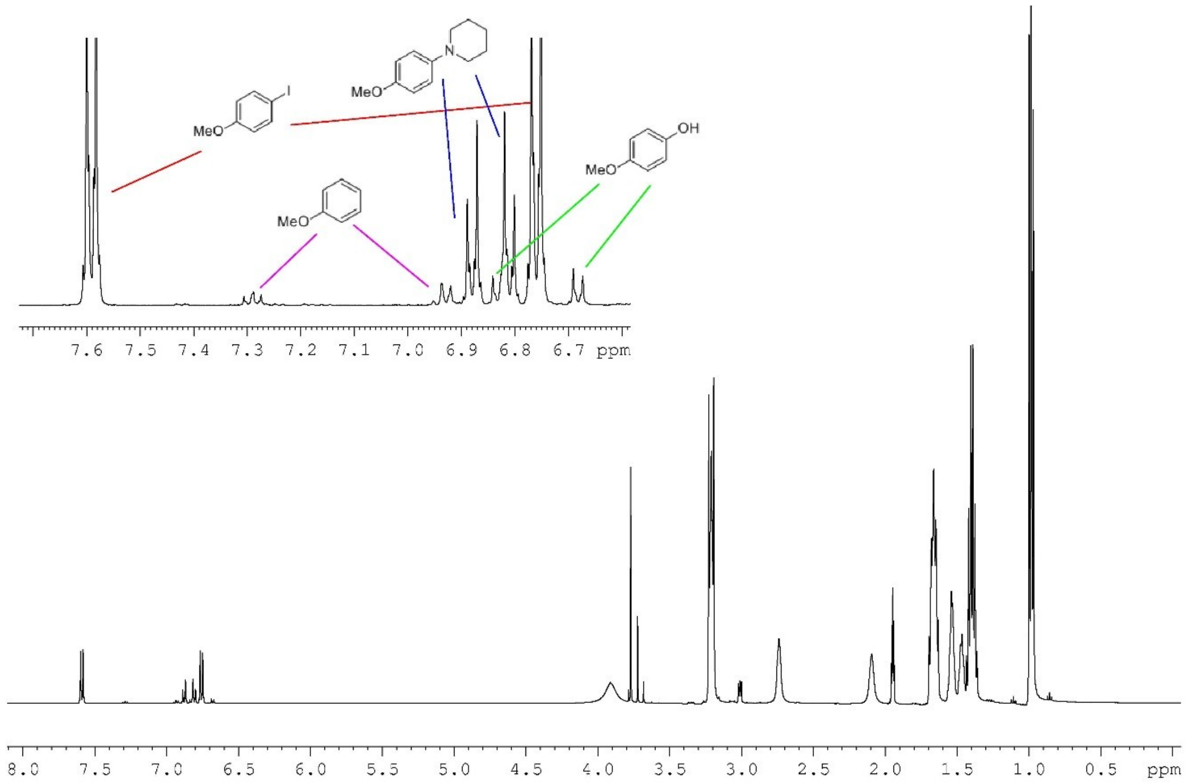

Figure S1. ^{1}H NMR spectrum of a typical reaction mixture in d_7-DMF

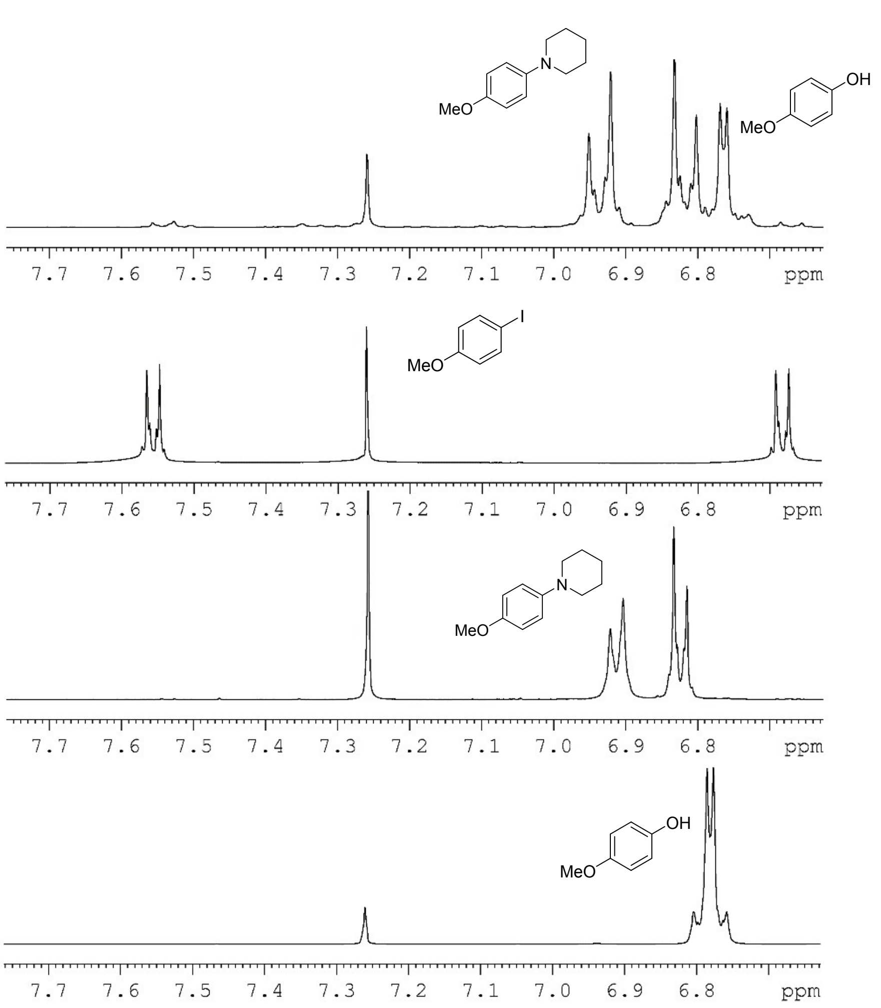

Figure S2. ^{1}H NMR spectra of a crude reaction mixture and standard samples

The NMR spectra of the main impurity matches well with literature data.[5]

4.4.2 4-Methoxyphenol speciation in reaction mixture (d_7-DMF)

4-Methoxyphenol appears to exist as 4-methoxyphenolate in the *in situ* NMR studies. When a reference spectrum of 4-methoxyphenol was taken in d_7-DMF, only one multiplet is seen in the aromatic region at 6.8 ppm, with the –OH peak seen at 9.1 ppm. When base is added, loss of the –OH signal is seen and splitting of the aromatic peaks is noted, giving the doublets seen in the *in situ* studies. Slight differences in ppm exist between reference samples and the *in situ* data as the *in situ* study is performed at 70 °C.

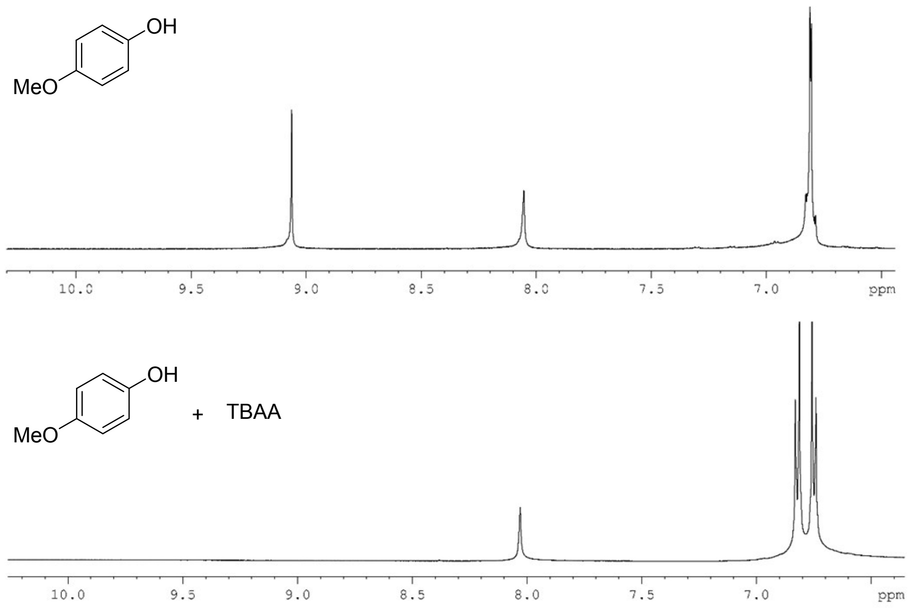

Figure S3. ^{1}H NMR spectra of 4-methoxyphenol with and without TBAA

4.4.3 Characterisation of anisole as a side product

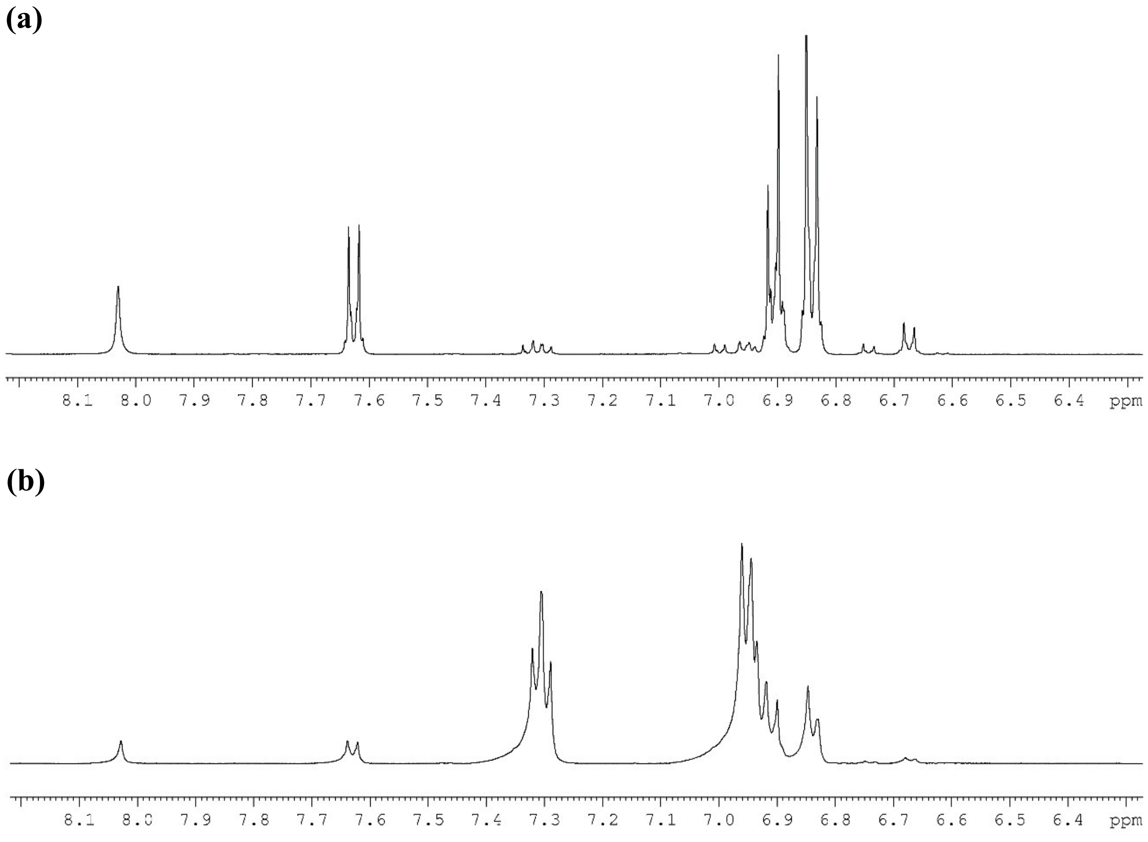

Figure S4. ^{1}H NMR spectra of (a) an *in situ* reaction mixture; and (b) the same reaction mixture spiked with anisole after exposure to air

4.4.4 4-Chloroanisole and *N,N*-dimethylanisidine reference spectra in d_7-DMF

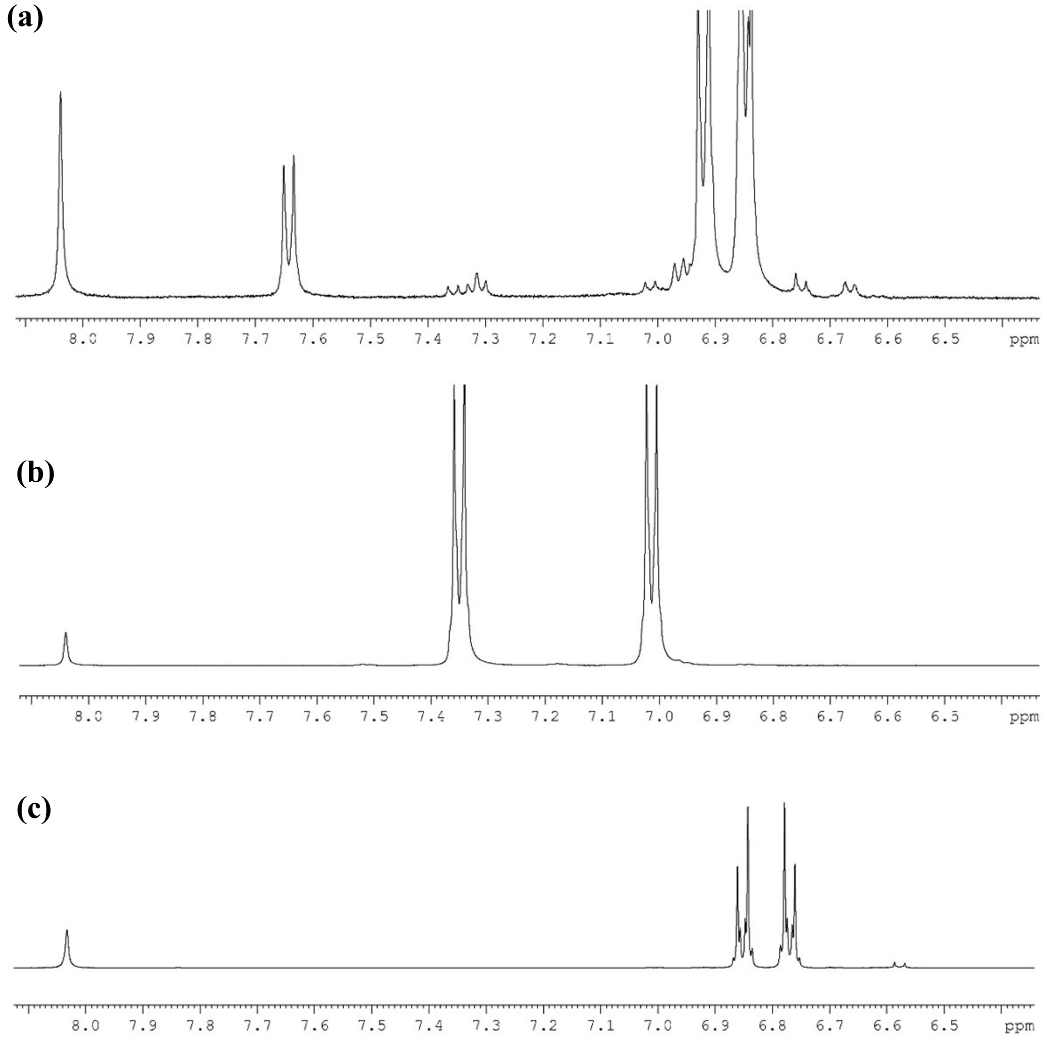

Figure S5. ^{1}H NMR spectra of (a) an *in situ* reaction mixture, (b) a sample of 4-chloroanisole from Sigma, and (c) a sample of *N,N*-dimethylanisidine from Sigma Aldrich at room temperature

4.4.5 ^{1}H COSY spectrum of reaction mixture post-reaction

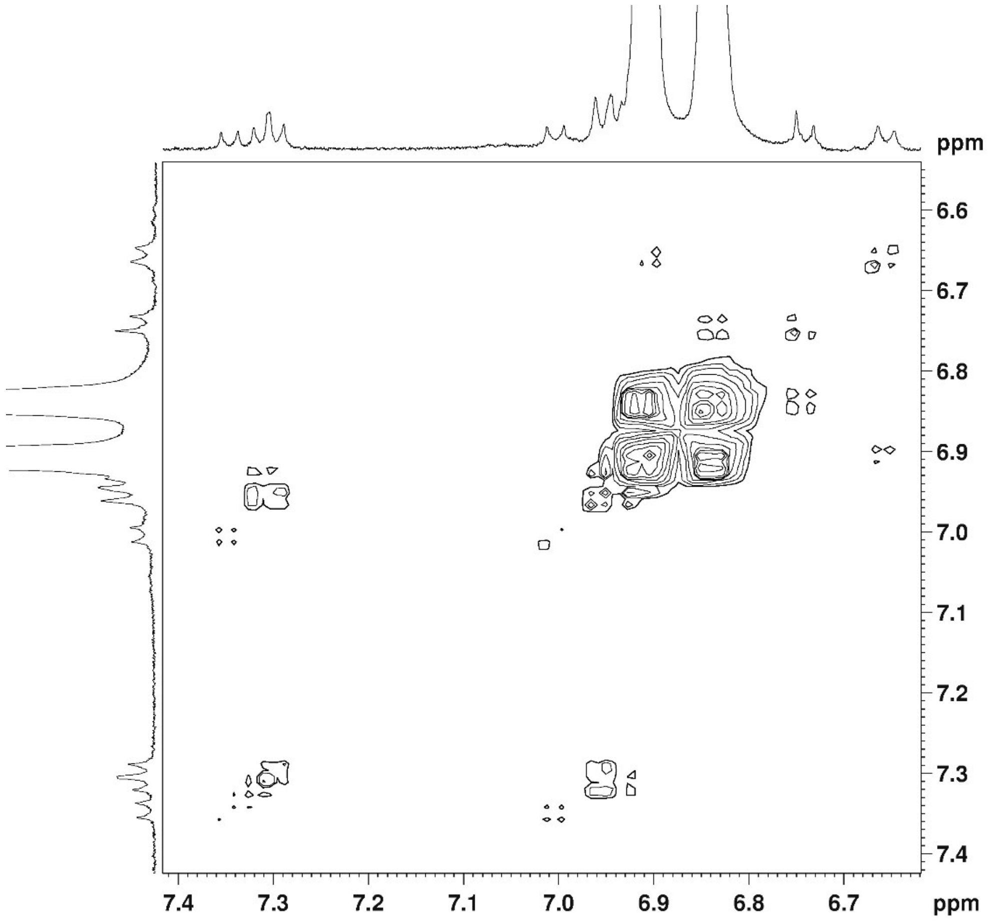

Figure S6. ^{1}H COSY spectrum of reaction mixture post-reaction

4.4.6 GC/MS characterisation of the products

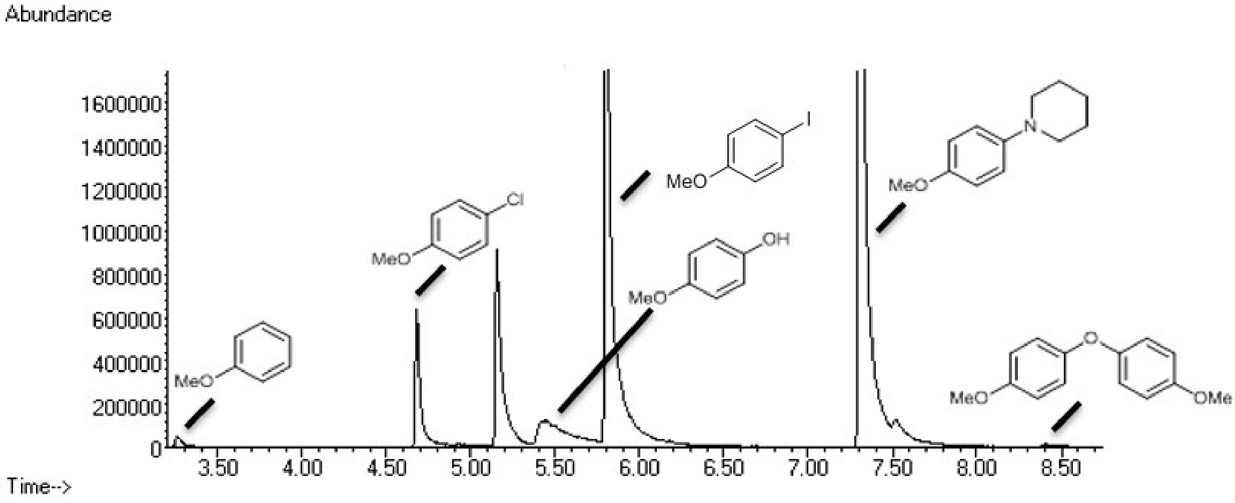

Figure S7. GC/MS chromatogram of the reaction mixture

Retention time (min)	m/z	Other	Compound
8.34	230	215 Fragment (-CH_3)	Bis-4,4'-methoxy phenylether
7.30	191	176 Fragment (-CH_3)	Coupling Product
5.80	234	219 Fragment (-CH_3)	4-Iodoanisole
5.40	124	109 Fragment (-CH_3)	4-Methoxyphenol
4.67	142	127 Fragment (-CH_3)	4-Chloroanisole
		Cl splitting pattern	
3.23	108	93 Fragment (-CH_3)	Anisole

4.4.7 Origin of *N,N*-dimethylanisidine

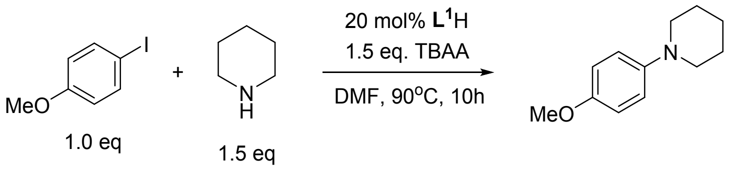

GC-MS analysis of the above reaction shows the presence of an impurity which appears on the chromatogram at 5.8 minutes with a mass of 151 (136 –CH_3), overlapping slightly with 4-methoxyphenol (m/z = 234).

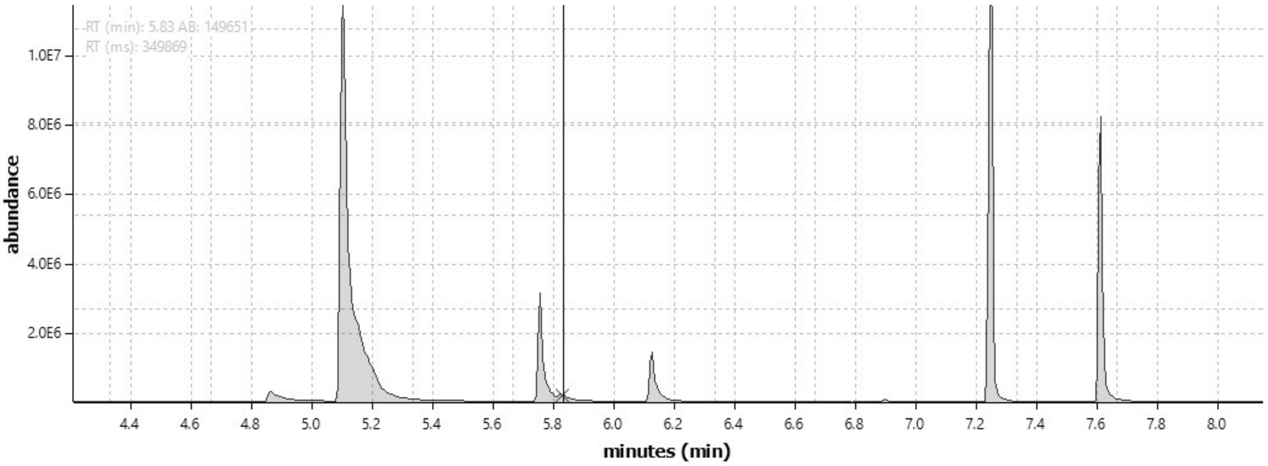

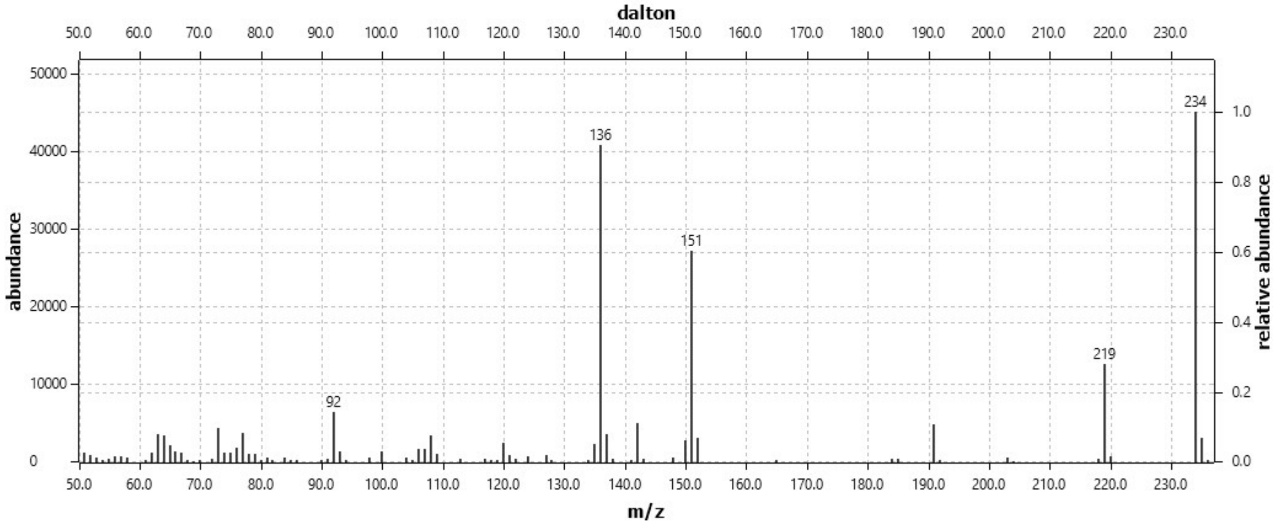

Figure S8. GC profile of the reaction mixture and MS data for the peak at 5.8 minutes in reaction using DMF

In d_7-DMF used in NMR experiments, the peak at 5.8 is seen as having a mass of 157 (142 $-CH_3$), showing that the origin of this impurity is reaction with the DMF solvent used in the reaction.

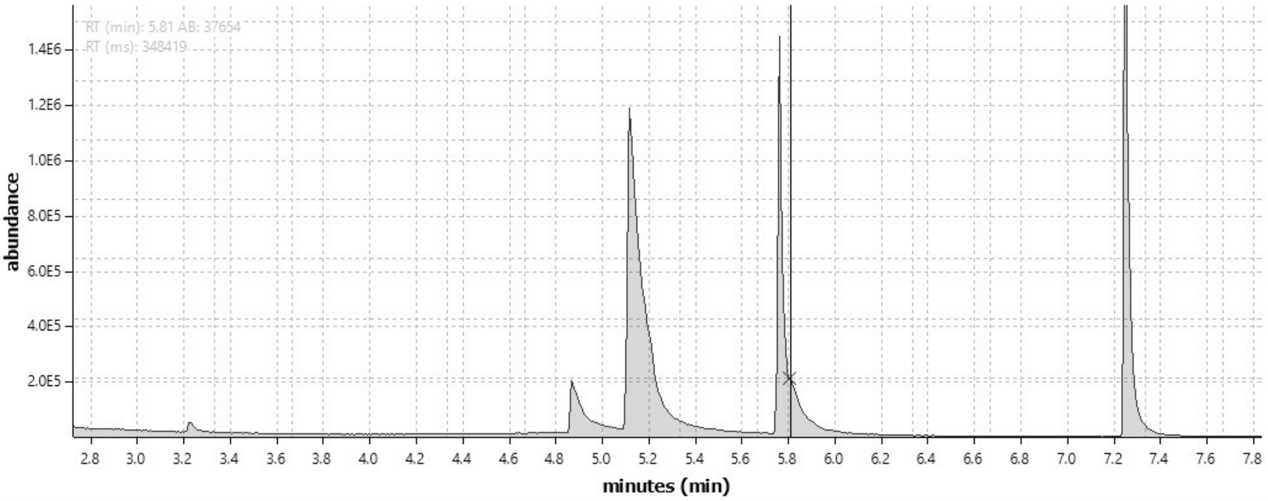

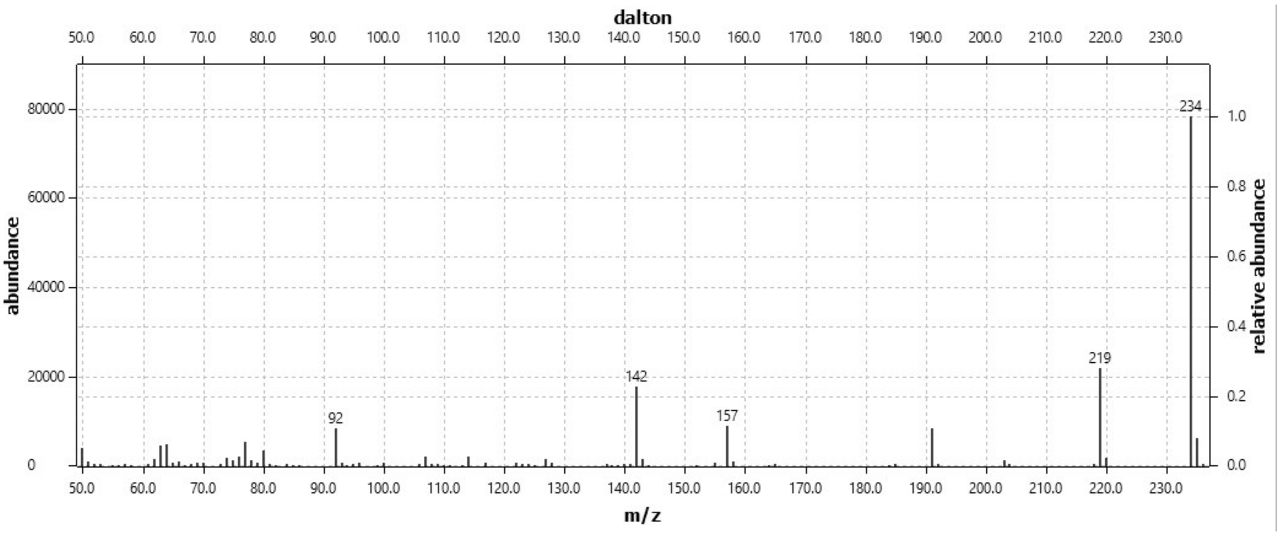

Figure S9. GC profile of the reaction mixture and MS data for the peak at 5.8 minutes in reaction using d_7-DMF

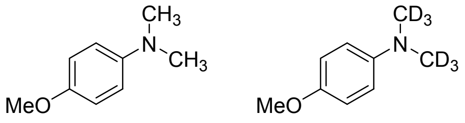

This can be confirmed with the GC/MS of pure *N,N*-dimethylanisidine, which appears on the chromatogram at 5.8 minutes with the same mass splitting of 151/136.

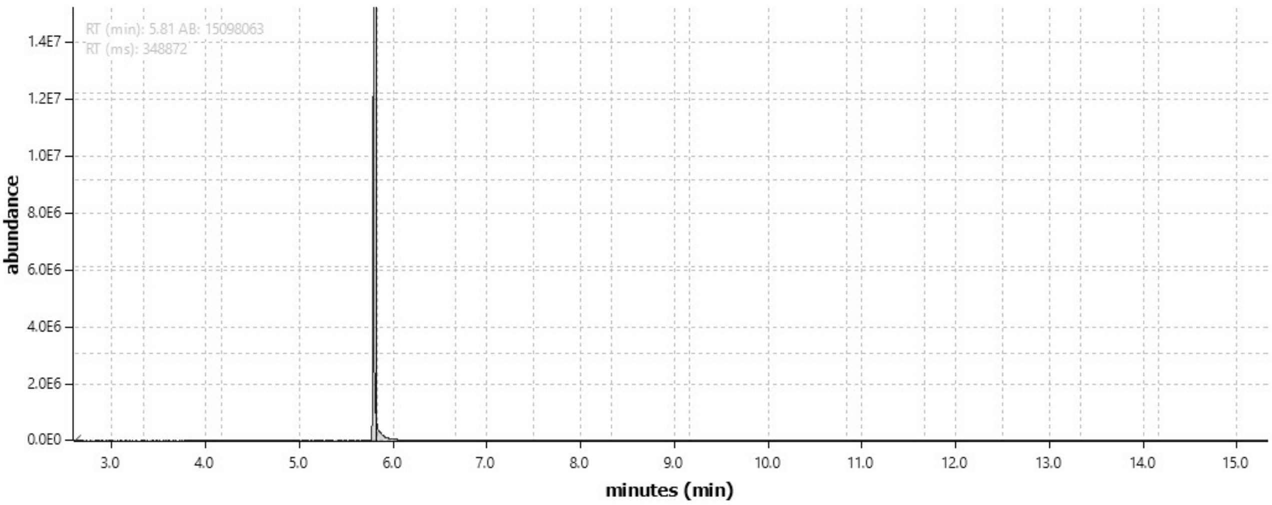

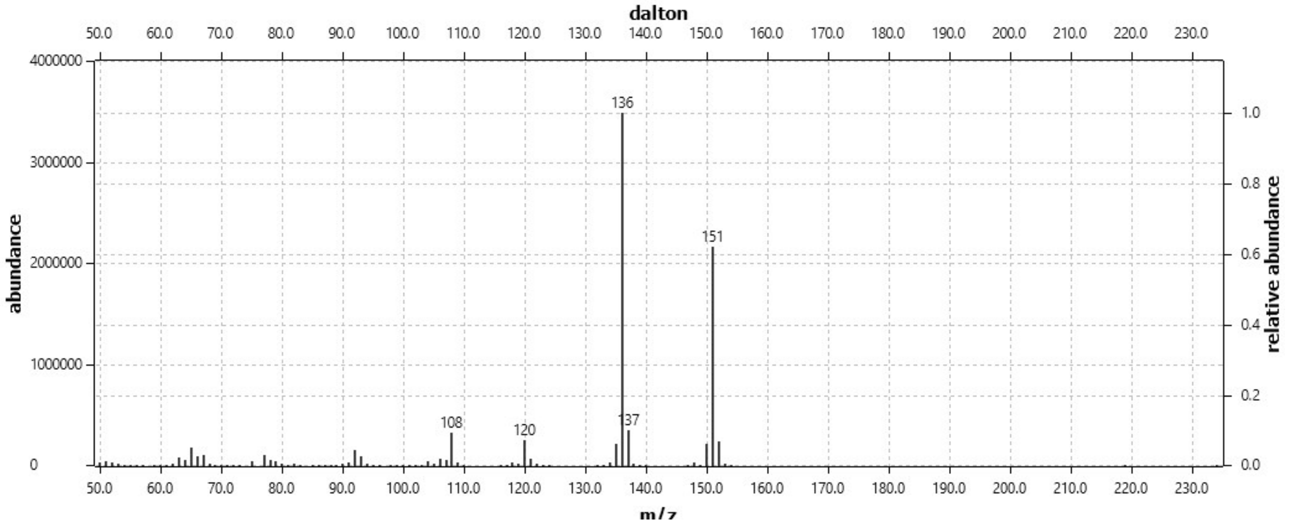

Figure S10. GC/MS data of commercial *N,N*-dimethylanisidine

4.5 Kinetic data in d_7-DMF

4.5.1 Standard conditions

[**1**]$_0$ = 0.125 M was employed unless otherwise stated (protocol in section 4.1).

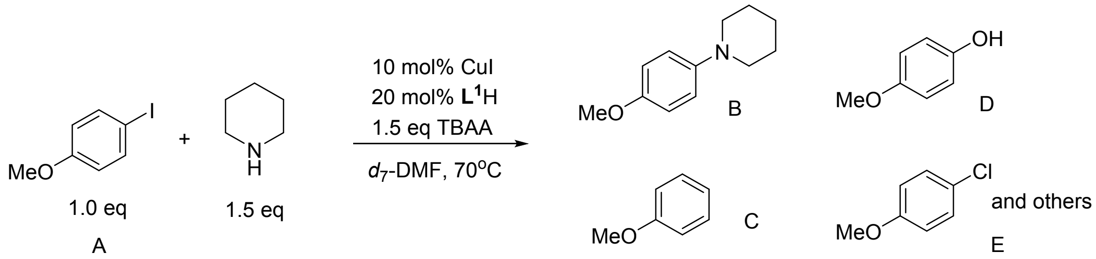

Time (minutes)	[A] (M)	[B] (M)	[C] (M)	[D] (M)	[E] (M)
0	0.10927	0.01603	0	0	0
16	0.0904	0.031	9.91964×10^{-4}	0.00135	0.00125
26	0.08011	0.03906	0.00144	0.00188	0.00276
36	0.07367	0.04441	0.00173	0.00208	0.00292
46	0.06864	0.04855	0.00229	0.00233	0.00345
56	0.06415	0.0522	0.0023	0.00263	0.00338
66	0.06222	0.05441	0.00413	0.00278	0.0017
76	0.05794	0.05749	0.00288	0.00281	0.00341
86	0.05513	0.05997	0.00391	0.00298	0.00325

96	0.05306	0.06165	0.00343	0.00318	0.00307
106	0.05053	0.06353	0.00401	0.00328	0.00391
116	0.0486	0.06492	0.00303	0.00331	0.00464
126	0.04647	0.06706	0.00441	0.00331	0.004
136	0.04541	0.06771	0.00388	0.00351	0.00376
146	0.04426	0.06891	0.00476	0.00353	0.00378
156	0.04208	0.07013	0.00371	0.00356	0.00485
166	0.04053	0.07132	0.0046	0.00358	0.00522
176	0.0396	0.07209	0.00361	0.00386	0.00519
186	0.039	0.07278	0.00501	0.00386	0.0046
196	0.0383	0.07346	0.00411	0.00386	0.0045
206	0.03757	0.07442	0.00504	0.00378	0.00443
216	0.03662	0.07466	0.00426	0.00411	0.00453
226	0.03677	0.07519	0.00523	0.00401	0.00405

4.5.2 Reaction with 2 x $[2]_0$

Time (minutes)	[A] (M)	[B] (M)	[C] (M)	[D] (M)	[E] (M)
0	0.09798	0.02382	5×10^{-4}	0.00112	0.00157
13	0.07917	0.0407	0.00171	0.00112	0.00229
23	0.0686	0.04994	0.00258	0.00135	0.00254
33	0.0611	0.05679	0.00336	0.00125	0.0025
43	0.05602	0.06112	0.00358	0.00175	0.00252
53	0.05155	0.06446	0.00399	0.0017	0.0033
63	0.04833	0.06744	0.00414	0.00175	0.00335
73	0.0456	0.06901	0.00446	0.00198	0.00395
83	0.04295	0.0711	0.00474	0.00215	0.00406

93	0.04102	0.07324	0.00471	0.00213	0.0039
103	0.03948	0.0751	0.00474	0.0021	0.00359
113	0.0377	0.07594	0.005	0.00213	0.00424
123	0.0363	0.07805	0.00499	0.00225	0.00341
133	0.03492	0.07871	0.00515	0.0024	0.00381
143	0.0337	0.07988	0.00505	0.00237	0.004
153	0.03255	0.08032	0.00525	0.0025	0.00437
163	0.03163	0.08201	0.00519	0.00245	0.00372
173	0.03053	0.08239	0.00528	0.00255	0.00426
183	0.02965	0.08284	0.00547	0.00255	0.00449
193	0.02888	0.08375	0.00539	0.0025	0.00449
203	0.02795	0.08438	0.00545	0.0026	0.00463
213	0.0273	0.08506	0.00544	0.00263	0.00458
223	0.0266	0.08614	0.00556	0.00237	0.00432
233	0.02598	0.08599	0.00559	0.00273	0.00473
243	0.0252	0.08624	0.00589	0.00255	0.00513

4.5.3 Reaction with 2 x $[TBAA]_0$

Time (minutes)	[A] (M)	[B] (M)	[C] (M)	[D] (M)	[E] (M)
0	0.11674	0.00806	0	0	0
13	0.10129	0.01878	8.6112×10^{-4}	0.00187	0.002
23	0.09358	0.02502	0.00116	0.00267	0.00237
33	0.08761	0.0299	0.00158	0.00332	0.00238
43	0.08217	0.0336	0.00196	0.00397	0.00311
53	0.0775	0.03707	0.00235	0.00477	0.00312
63	0.07371	0.04027	0.00238	0.00512	0.00332

73	0.06979	0.04281	0.00276	0.00564	0.00381
83	0.06652	0.04524	0.00295	0.00619	0.00391
93	0.06365	0.04745	0.00312	0.00651	0.00407
103	0.06133	0.04965	0.00318	0.00661	0.00403
113	0.05896	0.05118	0.00326	0.00701	0.00439
123	0.05708	0.05242	0.00337	0.00774	0.00419
133	0.05504	0.0542	0.00361	0.00764	0.00432
143	0.05302	0.0555	0.00358	0.00821	0.00449
153	0.05122	0.05666	0.00368	0.00836	0.00488
163	0.04965	0.05806	0.00381	0.00854	0.00475
173	0.0481	0.05913	0.00391	0.00876	0.0049
183	0.04665	0.06018	0.00403	0.00889	0.00505
193	0.04553	0.06111	0.00392	0.00906	0.00518
203	0.0444	0.06206	0.00404	0.00929	0.005
213	0.04336	0.06274	0.00428	0.00919	0.00524
223	0.04273	0.06367	0.00401	0.00953	0.00485
233	0.04101	0.06473	0.00409	0.00956	0.0054
243	0.04046	0.06552	0.00431	0.00956	0.00495

4.5.4 Reaction with 2 x $[L^1H]_0$

Time (minutes)	[A] (M)	[B] (M)	[C] (M)	[D] (M)	[E] (M)
0	0.11284	0.00728	0.0022	0.00185	6.24×10^{-4}
20	0.08956	0.02581	0.00387	0.00447	6.7392×10^{-4}
30	0.07867	0.03486	0.00456	0.00514	9.984×10^{-4}
40	0.07246	0.04036	0.00492	0.00547	0.00102
50	0.06744	0.04389	0.00534	0.00602	0.00112

60	0.06345	0.04722	0.00548	0.00649	0.0011
70	0.05923	0.05064	0.00574	0.00686	0.00115
80	0.05641	0.05315	0.00567	0.00724	9.984×10^{-4}
90	0.05341	0.05547	0.00609	0.00726	0.00122
100	0.05119	0.05774	0.00624	0.00731	0.00107
110	0.0492	0.05953	0.0059	0.00794	7.9872E-4
120	0.04643	0.06203	0.00636	0.00744	0.00115
130	0.0443	0.06359	0.00655	0.00774	0.0011
140	0.04271	0.06448	0.00675	0.00811	0.00112
150	0.04031	0.06638	0.00685	0.00829	0.00127
160	0.03906	0.06783	0.00685	0.00839	0.00102
170	0.03776	0.06885	0.00678	0.00854	0.00112
180	0.03642	0.06984	0.00695	0.00864	0.00112
190	0.03517	0.07099	0.00708	0.00856	0.00115
200	0.0336	0.07231	0.00721	0.00856	0.00125
210	0.03257	0.07296	0.00726	0.00881	0.00117
220	0.03135	0.07406	0.00725	0.00889	0.00117
230	0.03035	0.07502	0.00745	0.00881	0.00112
240	0.0291	0.07607	0.00738	0.00904	0.0011
250	0.02848	0.07666	0.00725	0.00926	9.2352E-4

4.5.5 Reaction with 0.5 x $[1]_0$

Time (minutes)	[A] (M)	[B] (M)	[C] (M)	[D] (M)	[E] (M)
0	0.05383	0.00749	4.4928×10^{-4}	3.3696×10^{-4}	2.9952×10^{-4}
20	0.03605	0.02013	0.0021	0.00298	0.00114
30	0.03004	0.02417	0.00286	0.00366	0.00167

40	0.02592	0.02774	0.00347	0.00408	0.00119
50	0.02291	0.02978	0.00358	0.0049	0.00122
60	0.02058	0.03191	0.00366	0.00498	0.00127
70	0.01813	0.03313	0.00407	0.0055	0.00156
80	0.01604	0.03497	0.00422	0.00575	0.00142
90	0.0143	0.03602	0.00427	0.00631	0.00149
100	0.01246	0.03691	0.0045	0.00628	0.00226
110	0.01153	0.03692	0.0048	0.00681	0.00234
120	0.01099	0.03776	0.0048	0.00676	0.00207
130	0.00955	0.03907	0.00489	0.00684	0.00206
140	0.00926	0.0389	0.00505	0.00704	0.00215
150	0.0083	0.03971	0.00495	0.00739	0.00206
160	0.00771	0.04028	0.00515	0.0071	0.00215
170	0.00725	0.04053	0.0051	0.00738	0.00215
180	0.00664	0.04092	0.00524	0.00733	0.00227
190	0.00609	0.0412	0.00544	0.00769	0.00198
200	0.00575	0.04129	0.00522	0.00766	0.00247
210	0.00512	0.04249	0.00525	0.00769	0.00186
220	0.00495	0.0422	0.00555	0.00735	0.00235
230	0.00483	0.04234	0.0054	0.00761	0.00222
240	0.00439	0.04276	0.00548	0.00761	0.00215
250	0.00394	0.04277	0.00565	0.00764	0.00240

4.5.6 Reaction with product inhibition (0.62 eq. of product at the beginning)

**Time (minutes)**	**[A] (M)**	**[B] (M)**	**All Side Products (M)**
0	0.125	0	0

15	0.1149	0.00768	0.00242
25	0.10872	0.01196	0.00432
35	0.10583	0.01505	0.00412
45	0.10444	0.01576	0.00479
55	0.10143	0.01927	0.0043
65	0.09763	0.02127	0.0061
75	0.09494	0.02319	0.00687
85	0.09347	0.02458	0.00695
95	0.09129	0.02545	0.00826
105	0.09038	0.02749	0.00713
115	0.0888	0.02931	0.00689
125	0.08979	0.02763	0.00758
135	0.08721	0.0302	0.00758
145	0.08666	0.02909	0.00925
155	0.08567	0.03052	0.00881
165	0.08579	0.03075	0.00846
175	0.08369	0.03236	0.00895
185	0.08381	0.03175	0.00945
195	0.08151	0.0338	0.00968
205	0.08159	0.03396	0.00945
215	0.08139	0.03434	0.00927
225	0.08108	0.03426	0.00966
235	0.07965	0.03519	0.01016
245	0.0789	0.03614	0.00996

4.5.7 Product distribution ratios in d_7-DMF

Experiment	[A]	[B]	[C]	[D]	[E]
4.5.1 Standard	27	63	4	3	2
4.5.2 2 x $[2]_0$	20	69	5	2	2
4.5.3 2 x $[TBAA]_0$	32	54	3	8	2
4.5.4 2 x $[L^1H]_0$	23	62	6	7	1
4.5.5 0.5 x $[1]_0$	6	69	9	12	1

4.5.8 Same [“excess”] – Standard Conditions

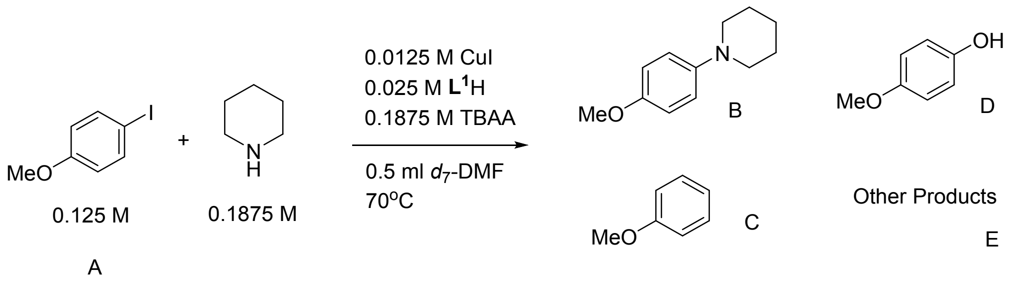

Time (minutes)	[A] (M)	[B] (M)	[C] (M)	[D] (M)	[E] (M)
0	0.114013	0.010563	0.000000	0.000425	0.000000
10	0.104187	0.019716	0.000000	0.001097	0.000000
20	0.092545	0.027440	0.001193	0.002832	0.000990
30	0.086530	0.032762	0.001334	0.003409	0.000965
40	0.081524	0.036447	0.001686	0.004013	0.001329

50	0.077197	0.040128	0.001605	0.004514	0.001555
60	0.073476	0.042675	0.002058	0.004945	0.001845
70	0.070359	0.045342	0.001847	0.005621	0.001832
80	0.067692	0.047472	0.002351	0.005551	0.001934
90	0.064867	0.049611	0.002260	0.005977	0.002285
100	0.062830	0.051961	0.002038	0.005999	0.002171
110	0.060521	0.052466	0.002664	0.006711	0.002639
120	0.058991	0.054304	0.002694	0.006585	0.002426
130	0.057338	0.055616	0.002745	0.006636	0.002665
140	0.055762	0.056491	0.002816	0.006989	0.002941
150	0.054183	0.057651	0.002624	0.007414	0.003129
160	0.053325	0.058743	0.002826	0.007140	0.002967
170	0.052579	0.059469	0.002907	0.007217	0.002829
180	0.051479	0.059954	0.002968	0.007419	0.003181
190	0.050342	0.061042	0.002917	0.007443	0.003256
200	0.049219	0.061304	0.003008	0.007973	0.003496
210	0.048753	0.062469	0.002866	0.007593	0.003319
220	0.047877	0.063153	0.002907	0.007795	0.003269
230	0.047076	0.063761	0.002947	0.007821	0.003395

240	0.046081	0.064104	0.003069	0.008125	0.003622

4.5.9 Same [“excess”] conditions – [“Excess”] = 0.0625 M

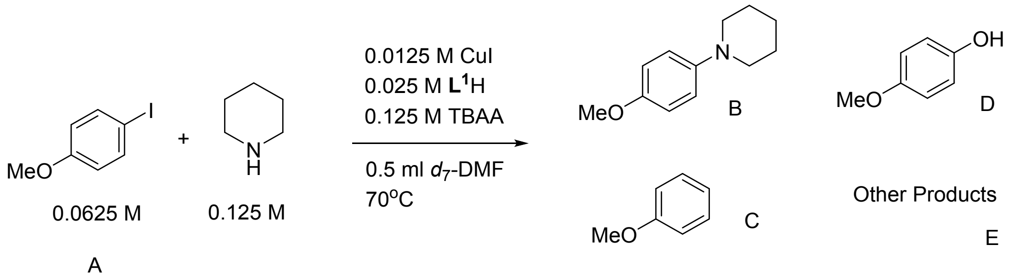

Time (minutes)	[A] (M)	[B] (M)	[C] (M)	[D] (M)	[E] (M)
0	0.051556	0.009377	0.000564	0.000251	0.000752
10	0.041384	0.016654	0.000815	0.001981	0.001665
20	0.036901	0.020040	0.001282	0.002638	0.001640
30	0.033359	0.021878	0.001762	0.003298	0.002203
40	0.031074	0.023671	0.002016	0.003478	0.002262
50	0.029178	0.025176	0.002080	0.003479	0.002587
60	0.027574	0.026521	0.002398	0.003735	0.002272
70	0.026081	0.027654	0.002602	0.003877	0.002286
80	0.024529	0.028045	0.002487	0.004292	0.003147
90	0.023775	0.029134	0.002793	0.004158	0.002639
100	0.022700	0.029996	0.002921	0.004173	0.002709
110	0.021720	0.030809	0.002998	0.004326	0.002647
120	0.020791	0.031402	0.003088	0.004391	0.002828

130	0.019959	0.031881	0.003126	0.004417	0.003117
140	0.019114	0.032496	0.003331	0.004687	0.002872
150	0.018449	0.032978	0.003241	0.004584	0.003248
160	0.017634	0.033545	0.003550	0.004944	0.002827
170	0.017108	0.033335	0.003640	0.005047	0.003370
180	0.016358	0.034427	0.003421	0.004790	0.003504
190	0.015889	0.034654	0.003408	0.004777	0.003773
200	0.015238	0.035180	0.003550	0.004868	0.003664
210	0.014693	0.035440	0.003550	0.004906	0.003911
220	0.014138	0.035840	0.003601	0.004983	0.003937
230	0.013587	0.036226	0.003730	0.005214	0.003743
240	0.013156	0.036531	0.003730	0.005125	0.003958

4.6 Kinetic data in d_3-MeCN

4.6.1 Standard conditions

[**1**]$_0$ = 0.125 M was employed unless otherwise stated (protocol in section 4.1).

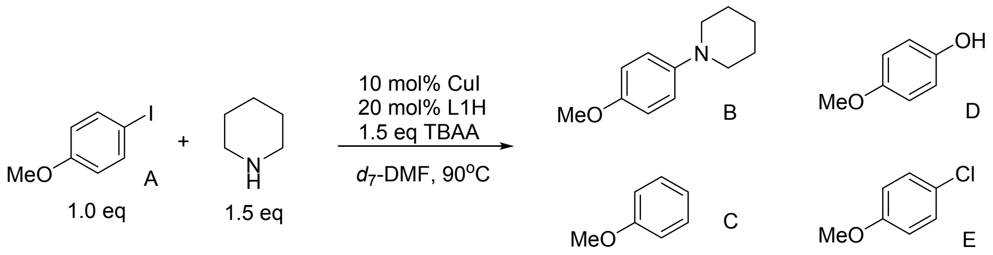

Time (minutes)	[A] (M)	[B] (M)	[C] (M)	[D] (M)	[E] (M)

0	0.100435	0.020705	0.002553	0.000000	0.001307
15	0.086803	0.032309	0.003059	0.000755	0.002075
25	0.081440	0.037610	0.003646	0.001121	0.001184
35	0.077679	0.041018	0.003758	0.001259	0.001285
45	0.074534	0.043491	0.003951	0.001713	0.001310
55	0.072345	0.046428	0.003971	0.001588	0.000668
65	0.069157	0.048042	0.004246	0.001929	0.001626
75	0.067149	0.049798	0.004409	0.001917	0.001728
85	0.065332	0.051481	0.004592	0.001905	0.001690
95	0.064082	0.053122	0.004480	0.001867	0.001450
105	0.062769	0.054279	0.004571	0.002119	0.001261
115	0.061686	0.055909	0.004592	0.001829	0.000984
125	0.060666	0.056945	0.004500	0.001841	0.001047
135	0.059486	0.057770	0.004653	0.001930	0.001161
145	0.058780	0.058994	0.004765	0.001956	0.000505
155	0.057565	0.059597	0.004734	0.001931	0.001174
165	0.056799	0.060345	0.004765	0.001842	0.001249
175	0.055205	0.061165	0.005081	0.002235	0.001313
185	0.054089	0.061704	0.005153	0.002399	0.001654
195	0.053453	0.062562	0.005347	0.002400	0.001238
205	0.053223	0.062898	0.005204	0.002299	0.001377
215	0.052984	0.063740	0.004969	0.002070	0.001237
225	0.051776	0.064104	0.005255	0.002223	0.001642

4.6.2 Reaction with 2 x $[2]_0$

Time (minutes)	[A] (M)	[B] (M)	[C] (M)	[D] (M)	[E] (M)

0	0.096364	0.023350	0.002422	0.001017	0.001846
12	0.081656	0.039156	0.002806	0.000578	0.000804
22	0.073698	0.046087	0.003403	0.000856	0.000956
32	0.068380	0.051490	0.003444	0.000831	0.000856
42	0.064085	0.055221	0.003616	0.001045	0.001032
52	0.060564	0.058511	0.003758	0.001121	0.001045
62	0.057708	0.061311	0.003890	0.001146	0.000945
72	0.055384	0.063662	0.003951	0.001096	0.000907
82	0.052833	0.065541	0.004256	0.001336	0.001034
92	0.051070	0.067584	0.004266	0.001286	0.000794
102	0.049430	0.069192	0.004134	0.001248	0.000996
112	0.047850	0.070830	0.004215	0.001160	0.000945
122	0.046194	0.071661	0.004297	0.001652	0.001198
132	0.044836	0.073495	0.004337	0.001261	0.001072
142	0.043347	0.074554	0.004765	0.001451	0.000883
152	0.042040	0.075690	0.004683	0.001476	0.001110
162	0.041172	0.076567	0.004561	0.001640	0.001060
172	0.040348	0.077569	0.004347	0.001803	0.000933
182	0.038910	0.078767	0.004836	0.001464	0.001022
192	0.037767	0.079811	0.004734	0.001489	0.001199
202	0.036807	0.080582	0.004898	0.001553	0.001161
212	0.036109	0.081240	0.004673	0.001653	0.001325
222	0.035027	0.082462	0.004898	0.001464	0.001149
232	0.034087	0.083128	0.005183	0.001528	0.001074
242	0.033220	0.083986	0.005255	0.001693	0.000846

4.6.3 Reaction with 2 x $[TBAA]_0$

Time (minutes)	[A] (M)	[B] (M)	[C] (M)	[D] (M)	[E] (M)
0	0.102070	0.019031	0.002806	0.000955	0.000138
14	0.090846	0.028164	0.003373	0.002064	0.000554
24	0.084940	0.033027	0.003646	0.002820	0.000567
34	0.079735	0.036477	0.004175	0.003252	0.001361
44	0.077495	0.038615	0.004114	0.003403	0.001374
54	0.074457	0.041218	0.004195	0.003807	0.001324
64	0.072439	0.042750	0.004276	0.004185	0.001349
74	0.070339	0.045021	0.004582	0.003860	0.001198
84	0.067183	0.047508	0.005132	0.004092	0.001086
94	0.064864	0.049873	0.005173	0.004041	0.001048
104	0.063569	0.051595	0.005214	0.004042	0.000581
114	0.062831	0.051896	0.005071	0.004192	0.001010
124	0.061077	0.054965	0.005081	0.003611	0.000265
134	0.060188	0.055705	0.005142	0.003612	0.000354
144	0.058695	0.057294	0.004796	0.004064	0.000151
154	0.056933	0.059343	0.004724	0.003659	0.000341
164	0.055610	0.060646	0.004857	0.003685	0.000202
174	0.054901	0.060296	0.005418	0.003892	0.000493
184	0.053042	0.061253	0.005285	0.004649	0.000771
194	0.053014	0.062181	0.005020	0.004368	0.000417
204	0.051275	0.063272	0.005674	0.004399	0.000379
214	0.051290	0.062946	0.005694	0.004804	0.000265
224	0.050498	0.064228	0.005735	0.004349	0.000190

234	0.049717	0.064705	0.005510	0.004347	0.000720
244	0.049166	0.065432	0.005950	0.004427	0.000025

4.6.4 Reaction with 2 x $[L^1H]_0$

Time (minutes)	[A] (M)	[B] (M)	[C] (M)	[D] (M)	[E] (M)
0	0.102871	0.016937	0.004083	0.000681	0.000428
12	0.088218	0.029419	0.005040	0.001187	0.001136
22	0.082437	0.034789	0.005285	0.001213	0.001276
32	0.078544	0.038867	0.005428	0.001087	0.001074
42	0.074541	0.041689	0.005623	0.001492	0.001656
52	0.071593	0.044449	0.005960	0.001606	0.001391
62	0.069564	0.046637	0.006104	0.001544	0.001151
72	0.067982	0.048333	0.006104	0.001506	0.001075
82	0.066193	0.049461	0.006257	0.001987	0.001101
92	0.064251	0.050958	0.006411	0.002165	0.001215
102	0.063029	0.052265	0.006565	0.002026	0.001114
112	0.062361	0.052980	0.006380	0.001937	0.001342
122	0.060898	0.054235	0.006637	0.001963	0.001267
132	0.059711	0.055214	0.006668	0.002153	0.001254
142	0.057968	0.056397	0.006935	0.002446	0.001255
152	0.057349	0.056969	0.006842	0.002420	0.001419
162	0.056515	0.057897	0.006976	0.002243	0.001369
172	0.055814	0.058754	0.006935	0.002231	0.001267
182	0.054924	0.059436	0.006914	0.002433	0.001293
192	0.054686	0.060020	0.006822	0.002217	0.001254
202	0.053667	0.060663	0.006894	0.002218	0.001559

212	0.052655	0.061342	0.007274	0.002346	0.001382
222	0.052352	0.062000	0.007161	0.002257	0.001230
232	0.051467	0.062724	0.007069	0.002586	0.001154
242	0.050653	0.063516	0.007418	0.002626	0.000786

4.6.5 Reaction with 0.5 x $[1]_0$

Time (minutes)	[A] (M)	[B] (M)	[C] (M)	[D] (M)	[E] (M)
0	0.049732	0.009162	0.002225	0.000744	0.000637
11	0.036993	0.021243	0.003118	0.000911	0.000234
21	0.032365	0.025245	0.003395	0.001204	0.000291
31	0.029161	0.027988	0.003632	0.001332	0.000387
41	0.026528	0.030609	0.003915	0.001270	0.000178
51	0.024486	0.032069	0.004065	0.001537	0.000343
61	0.022608	0.033706	0.004241	0.001551	0.000394
71	0.020980	0.034886	0.004458	0.001679	0.000496
81	0.019519	0.036487	0.004572	0.001629	0.000293
91	0.018010	0.037449	0.004925	0.001784	0.000331
101	0.017101	0.038482	0.004764	0.001834	0.000318
111	0.016219	0.039262	0.004821	0.001873	0.000325
121	0.014984	0.040082	0.005055	0.001938	0.000440
131	0.013489	0.041265	0.005472	0.001737	0.000536
141	0.013122	0.041925	0.005175	0.001927	0.000351
151	0.012193	0.042499	0.005477	0.001942	0.000390
161	0.011725	0.043240	0.005441	0.001890	0.000204
171	0.010886	0.043641	0.005697	0.001892	0.000384
181	0.010361	0.044146	0.005660	0.001956	0.000377

191	0.009862	0.044777	0.005504	0.002108	0.000249
201	0.009325	0.045403	0.005650	0.001943	0.000179
211	0.008943	0.045592	0.005524	0.002198	0.000243
221	0.008517	0.046002	0.005781	0.001918	0.000281
231	0.008296	0.046236	0.005660	0.002045	0.000262
241	0.007896	0.046366	0.005744	0.002174	0.000320

5. Kinetic experiments using inorganic bases

5.1 General procedure for reactions with automated sampling

All experiments were performed as outlined in general procedure above. Reactions were performed in a 4 mL vial with a stirrer disk in a 24 well plate within a glovebox filled with an N_2 atmosphere. Heating, solution additions and sampling were executed by a Freeslate automation robot.

Inside a nitrogen-filled glovebox reaction vials containing magnetic stirrer disks were loaded with the corresponding amounts of CuI (0.1 eq., 0.05 mmol), ligand (**L**^{1}H or **L**^{2}H, 0.1 mmol) and base (Cs_2CO_3 or K_3PO_4, 1.5 eq., see table below for quantities). Into separate vials, a solution of 4-iodoanisole **1** (0.625 M) in and a solution of 2-pyrrolidinone **4**/1,1'-biphenyl (0.441 M and 0.0294 M, respectively) were made up in degassed and anhydrous DMF. The reaction vials were loaded into a 24 well stainless steel heating plate and loosely fitted with septum seal caps to prevent pressure fluctuations during charging and sampling. The reaction vials were each charged with the 2-pyrrolidinone/1,1'-biphenyl solution (1.7 mL), heated to 90 °C before being left to equilibrate for 30 minutes. The 4-iodoanisole solution was pre-heated to 90 °C and the reaction initiated through the addition of this solution (0.8 mL) to each vial.

For analysis, HPLC vials were loosely fitted with caps and injected with 500 µl MeCN by the Freeslate automation robot. Sampling of reactions was also performed by the robot at the desired time intervals, whereby 30 µl of reaction mixture was added to the MeCN. The samples were removed from the glovebox and filtered using Whatman Mini-UniPrep syringeless filters (0.2 µm pore size) prior to analysis.

For experiments varying the quantities of 4-iodoanisole **1** and 2-pyrrolidinone **4**, separate solutions were made to adjust the reaction concentrations without changing the volume of the reaction mixtures.

The kinetic data above were collected using Cs_2CO_3 (99%) supplied by Chemetall (**Base1**) with particle size distribution D50 = 20 µm (the value of the particle diameter at 50% in the cumulative distribution), D90 = 50 µm (the value of the particle diameter at 90% in the cumulative distribution). Two other batches of Cs_2CO_3 supplied by Sigma-Aldrich (ReagentPlus® 99%, lot# BCBP3311V, **Base2**) and Acros Organic (99.5%, lot# A0359643, **Base3**) were included in the study for comparison.

5.2 HPLC calibration

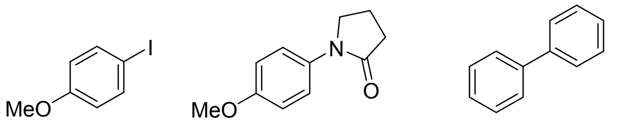

UV lamp set at 285 nm and concentration of internal standard (IS) held at 0.000113 M.

**[3] (M)**	**0.00113**	**0.00283**	**0.00566**	**0.00849**	**0.01018**	**0.01132**
**Area 3**	129665.5	323057.9	646656.8	929430.4	1136975.0	1257801.0
**Area IS**	53605.9	54349.68	55026.88	54723.1	55208.0	55779.7
**Area 3/IS**	2.418865574	5.944062	11.75165	16.98424	20.59438	22.54942

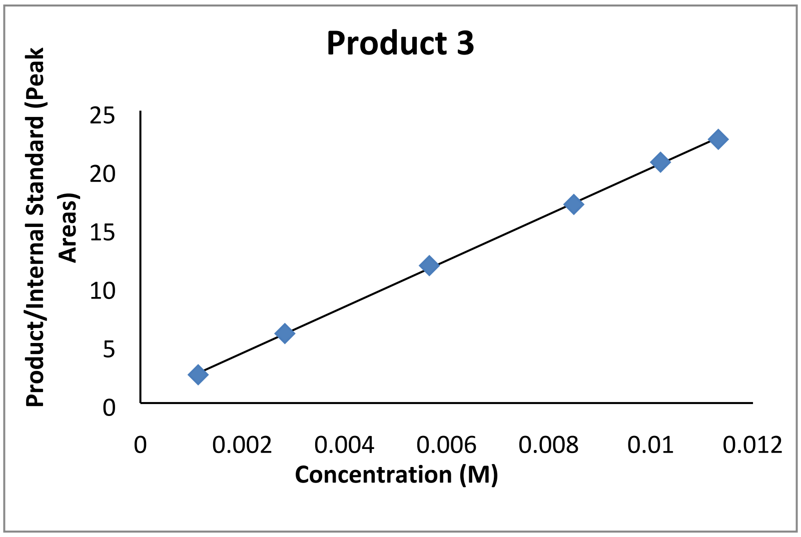

Figure S11. HPLC calibration curve for product **5**

**[1] (M)**	**0.00113**	**0.00283**	**0.00566**	**0.00849**	**0.01018**	**0.01132**
**Area 1**	82035.7	207296.1	419618.7	605277.8	745358.8	842654.1
**Area IS**	53605.9	54349.68	55026.88	54723.1	55208.0	55779.7
**Area 1/IS**	1.53035	3.81411	7.62570	11.06074	13.50091	15.10681

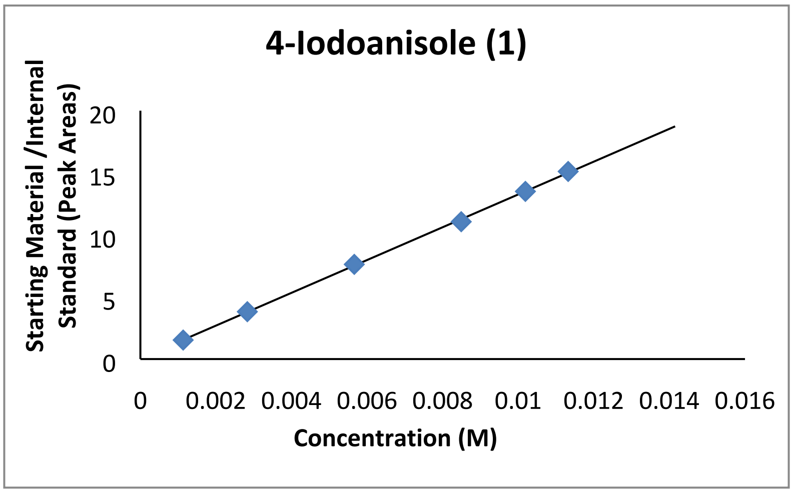

Figure S12. HPLC calibration curve for starting material **1**

5.3 Kinetic data with L^1H/Cs_2CO_3

5.3.1 Reaction conditions – experiment 1 (L^1H/Cs_2CO_3)

	MW (g.mol^{-1})	Density (g.mL^{-1})	Standard	0.5x [CuI/L^1]	2x [4]	Standard Duplicate	0.5x CuI	Product Inhibition	1eq H_2O
CuI (mg)	190.44		9.5	4.8	9.5	9.5	4.7	9.5	9.5
**L^1**H (mg)	103.12		10.3	5.2	10.3	10.3	10.3	10.3	10.3
1,1’-Biphenyl (mg)	154.21		7.7	7.7	7.7	7.7	7.7	7.7	7.7
Cs_2CO_3 (mg)	325.82		244	244	244	244	244	244	244
DMF (µl)			2500	2500	2500	2500	2500	2500	2500
**1** (mg)	234.03		117	117	117	117	117	117	117
**5** (mg)	191.23							47.8	
**4** (µl)	85.15	1.116	57	57	114	57	57	57	57
H_2O (µl)	18.02	1.000							9

5.3.2 Reaction under standard conditions

	1 - Cs_2CO_3 Std						
**Time (mins)**	PA ArI	PA Prod	PA Std	[SM]	[Prod]	ArI %	Prod Form %
30	498951.3	21127.02	29759.44	0.012638	0.000353	90.91419	2.536422944
64	879169.5	151273.9	55923.59	0.011851	0.001344	85.24635	9.664451386
96	855307.5	223396.9	56583.15	0.011394	0.001961	81.96593	14.10581671
123	797383.1	324889.6	56540.12	0.010631	0.002854	76.47307	20.52991678
155	708246.2	438896.4	55260.62	0.009661	0.003945	69.49711	28.37620957
182	599410.8	600918.2	54782.09	0.008248	0.005448	59.33135	39.19087543
214	412324.1	1081729	62591.18	0.004966	0.008584	35.72102	61.74666773
241	213691.6	1208572	54767.2	0.002941	0.01096	21.15753	78.84246559
272	138214.1	1486008	60347.64	0.001726	0.01223	12.4191	87.97694524
332	69222.41	1563819	59706.41	0.000874	0.013009	6.286718	93.57796119
391	44125.58	1570611	58555.47	0.000568	0.013322	4.086214	95.8317181

455	27178.8	1494011	54692.43	0.000375	0.013567	2.694643	97.59659443
513	19865.17	1556073	56459.71	0.000265	0.013689	1.907884	98.46899764
571	13814.27	1498591	54135.6	0.000192	0.013749	1.383704	98.90271685
633	9645.366	1496108	54040	0.000135	0.01375	0.967836	98.91355907

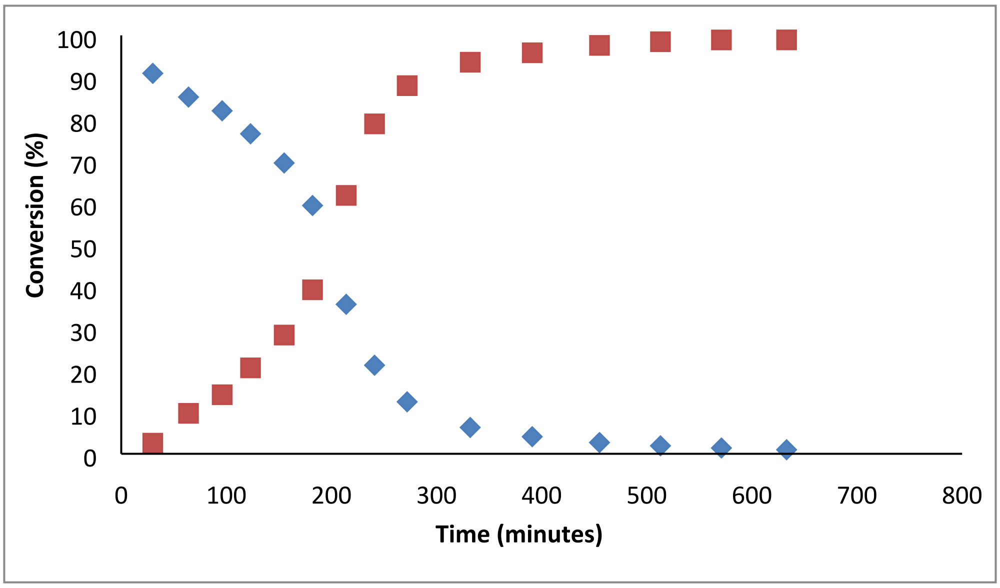

Figure S13. Conversion *vs* time under standard conditions

5.3.3 Reaction with 0.5 x $[CuI]_0$ and 0.5 x $[L^1H]_0$

	2 - Cs_2CO_3 0.5x Cu/L						
Time (mins)	PA ArI	PA Prod	PA Std	[SM]	[Prod]	ArI %	Prod Form %
30	1124885	12793.02	67790.99	0.012508	9.37E-05	99.25624	0.743759693
64	1030062	26688.04	63274.18	0.012271	0.000209	97.377416	1.662346771
96	916351.9	34014.44	57164.32	0.012084	0.000296	95.886802	2.345145784
124	897989.6	42435.16	56426.82	0.011996	0.000374	95.193511	2.963956656
155	869190.2	50866.77	54923.35	0.011929	0.00046	94.662821	3.650132841
183	943638.2	65705.97	59037.24	0.012049	0.000553	95.609496	4.386420785
214	865712.2	69317.35	54782.31	0.011912	0.000628	94.526759	4.986928162
241	836998.2	75867.35	53332.25	0.01183	0.000707	93.876351	5.606560672
273	920431.5	94964.97	58435.32	0.011873	0.000807	94.218808	6.405005821
332	880682.1	110840.6	56492.69	0.011751	0.000974	93.249936	7.732821284
391	817307.8	122708.5	53472.34	0.011522	0.00114	91.42776	9.044345825
456	862200.5	151564.5	56879.81	0.011426	0.001323	90.671698	10.50196976
514	870999.4	174273.9	58074.3	0.011306	0.00149	89.713018	11.82714605
571	817662	183848.3	55291.25	0.011147	0.001651	88.458386	13.10492681
633	770339.2	198405.3	53557	0.010842	0.00184	86.037413	14.60053034
693	849009	251720.5	59601.61	0.010738	0.002098	85.207122	16.64531658
753	761930.4	345405.9	58012.98	0.0099	0.002957	78.561858	23.46584348
840	652614.4	1074174	79048.29	0.006223	0.006749	49.383944	53.55667792

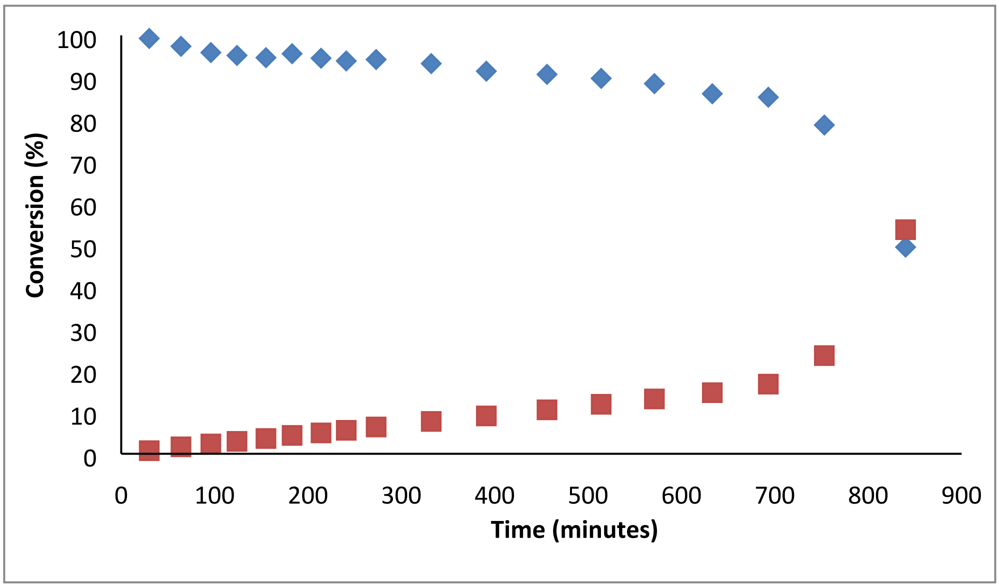

Figure S14. Conversion *vs* time with ½ x [CuI] and [**L**[1]H]

5.3.4 Reaction using 2 x [4]$_0$

	3 - Cs_2CO_3 2 x Amide						
**Time (mins)**	PA ArI	PA Prod	PA Std	[SM]	[Prod]	ArI %	Prod Form %
31	955348.5	27109.04	58907.93	0.012225	0.000229	94.17294	1.760711962
64	881045.8	73045.49	56791.54	0.011694	0.000639	90.08507	4.921048799
96	817376.4	105436.1	54066.25	0.011396	0.000969	87.78775	7.461238572
124	779018.6	143096.9	53189.74	0.01104	0.001336	85.0468	10.29319615
156	796517.9	197913.1	55944.04	0.010733	0.001757	82.67605	13.53532701
183	722931	221849.5	51968.16	0.010486	0.00212	80.77882	16.33311474
214	757748.1	284702.2	56019.99	0.010196	0.002524	78.54524	19.44445465
242	683490.1	301731.4	52224.71	0.009865	0.00287	75.99661	22.10509921
273	692725	356079.9	54515.76	0.009579	0.003244	73.78649	24.99041162
333	649292.7	441806.4	54987.78	0.008901	0.003991	68.56656	30.7407017
392	613512.5	524995.5	55732.05	0.008298	0.004679	63.92289	36.04113695
456	444620.9	824844.3	55914.8	0.005994	0.007327	46.17438	56.44079566
514	188432	1275712	59750.99	0.002377	0.010604	18.31249	81.6875059
571	63652.2	1329487	54794.99	0.000876	0.012051	6.745445	92.83063389
634	22013.35	1469802	57902.72	0.000287	0.012608	2.207624	97.11982023
694	9095.169	1518308	59349.71	0.000116	0.012706	0.889878	97.87894854
753	3629.191	1482465	57273.65	4.78E-05	0.012856	0.367954	99.03245728

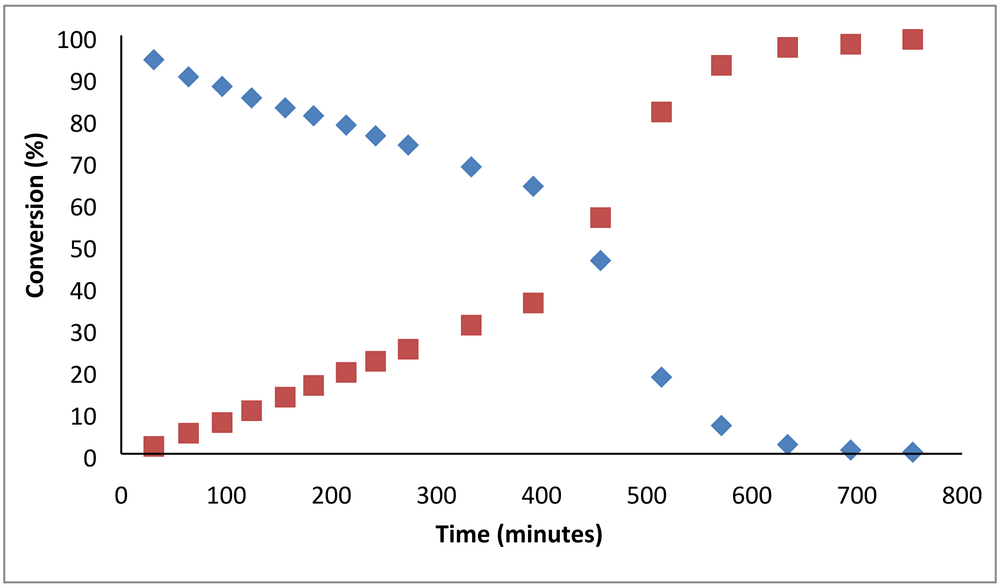

Figure S15. Conversion *vs* time with 2 x [**4**]

5.3.5 Repeat of reaction under standard conditions

	**4 - Cs_2CO_3 Standard Repeat**						
**Time (mins)**	PA ArI	PA Prod	PA Std	[SM]	[Prod]	ArI %	Prod Form %
31	1037740	45770.55	64791.31	0.012073	0.000351	92.827792	2.697649544
65	861996.8	92424.58	56403.08	0.01152	0.000814	88.574569	6.257498857
97	818517.6	163376.4	56530.99	0.010914	0.001435	83.916555	11.0361813
125	729028.3	229189	53313.49	0.010308	0.002135	79.252585	16.4162027
156	675564	320437	53437.91	0.00953	0.002978	73.269501	22.89862465
184	602074.8	374588.1	50076.58	0.009063	0.003715	69.68222	28.56507822
215	618281.2	558230.9	58077.5	0.008025	0.004774	61.699877	36.70473269
242	498490.2	554229.5	49820.29	0.007542	0.005525	57.99046	42.48146771
273	348323	847009.5	53567.33	0.004902	0.007853	37.686722	60.38157435
333	109582.7	1227578	53128.4	0.001555	0.011476	11.954228	88.23455635
392	47245.4	1341448	53916.27	0.000661	0.012357	5.0786219	95.01015088
456	23267.45	1410894	55226.41	0.000318	0.012689	2.4417894	97.55821056
515	14185.83	1516637	58691.41	0.000182	0.012834	1.4008328	98.6786388
572	8489.127	1461659	56269.36	0.000114	0.012902	0.874374	99.19509911
634	4979.362	1453973	56033.48	6.7E-05	0.012888	0.5150297	99.08883533

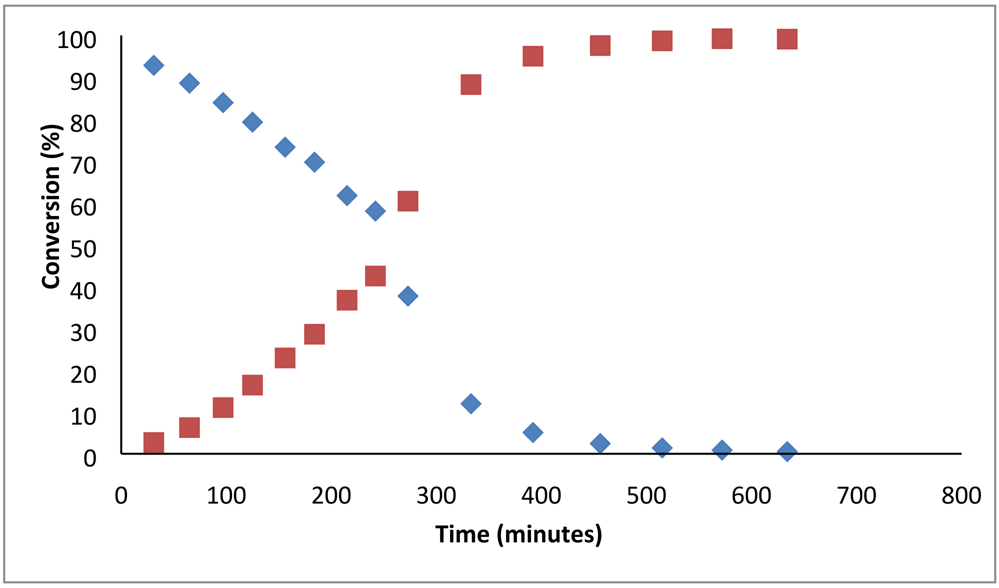

Figure S16. Conversion *vs* time under standard conditions (repeat)

5.3.6 Reaction with 0.5 x [CuI]

	5 - Cs_2CO_3 0.5 x CuI						
**Time (mins)**	PA ArI	PA Prod	PA Std	[SM]	[Prod]	ArI %	Prod Form %
31	1001805	17102.66	60057.87	0.012574	0.000141	94.30725	1.060805203
65	941578.2	42322.84	57940.68	0.01225	0.000363	91.87655	2.721027982
97	902367.2	58514.66	56302.38	0.012081	0.000516	90.61257	3.871504907
125	800768.1	70924.81	50903.31	0.011858	0.000692	88.93908	5.190318391
156	844400.3	102991.1	54298.01	0.011723	0.000942	87.92175	7.065741363
184	800178.2	132372.1	52194.76	0.011556	0.00126	86.67457	9.447380594
215	847612.4	197985.2	56940.01	0.011221	0.001727	84.16114	12.95259863
243	708995.1	201942	49186.32	0.010866	0.002039	81.49497	15.29410865
274	758364.7	289973	54586.47	0.010473	0.002638	78.54617	19.78856484
333	711714.1	352845.5	54048.74	0.009926	0.003242	74.4478	24.31872591
392	685271.4	451094.9	55768.48	0.009263	0.004017	69.47135	30.13151368
457	654148	558412.9	57289.22	0.008607	0.004841	64.55576	36.3098399
515	579752.6	696658	59396.41	0.007358	0.005825	55.18416	43.69193222
572	359376.7	1047975	59753.46	0.004534	0.008711	34.00312	65.33257194
634	203751.8	1325913	62249.21	0.002467	0.010579	18.50544	79.34562146
695	112256.8	1411357	59046.26	0.001433	0.011872	10.74861	89.04022383
754	78355	1653047	66008.15	0.000895	0.012438	6.711214	93.28878617

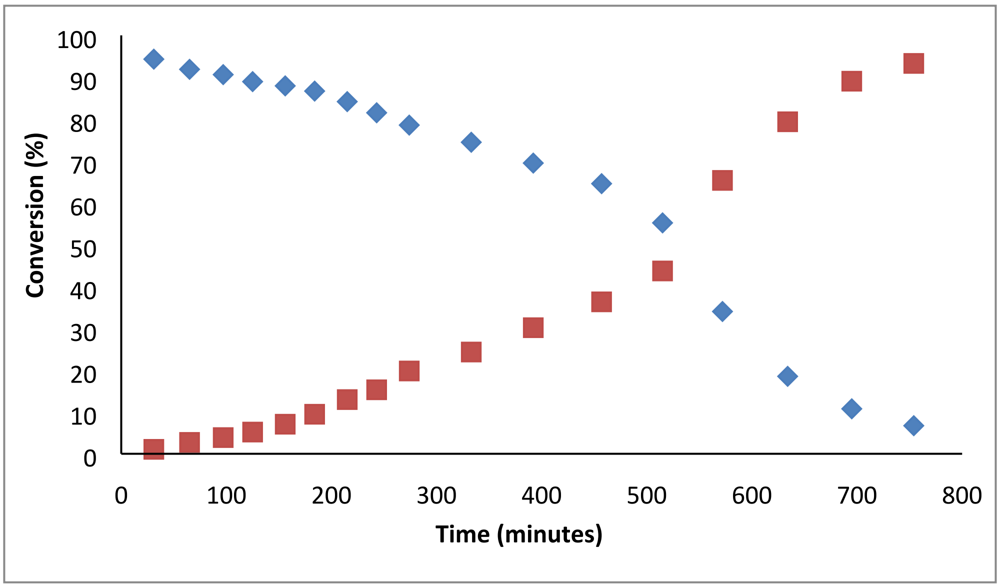

Figure S17. Conversion *vs* time with ½ [CuI]

5.3.7 Reaction with 0.5 eq. of product 5 at the beginning

	7 - Cs_2CO_3 Product Inhibition						
**Time (mins)**	PA ArI	PA Prod	PA Std	[SM]	[Prod]	ArI %	Prod Form %
33	910903.7	459431.7	58322.67	0.011773	0.003912	100	0
67	843571.5	555496.6	58216.72	0.010923	0.004739	92.77673	5.27035422
99	772858.2	791353	60802.72	0.009582	0.006464	81.38449	16.26794758
127	477088.9	760115.8	46156.43	0.007792	0.008179	49.6733	52.14509135
158	364852.8	1355119	61552.88	0.004468	0.010935	28.48559	69.70996456
186	327912.3	1809954	72043.29	0.003431	0.012478	21.87359	79.54994252
217	219230.2	1650212	61862.14	0.002671	0.013249	17.03065	84.46576869
244	149247.4	1398011	50820.53	0.002214	0.013663	14.11313	87.10382062
275	136168.4	1540463	54341.93	0.001889	0.014079	12.04196	89.75985034
335	117974.1	1644142	57321.14	0.001551	0.014246	9.890714	90.82184218
394	91071.04	1556874	52941.99	0.001297	0.014606	8.26677	93.11488061
458	75378.04	1608370	53814.68	0.001056	0.014844	6.731316	94.63486322
517	60251.01	1591394	52825.14	0.00086	0.014963	5.481249	95.39002559
574	51067.94	1663114	54634.89	0.000705	0.015119	4.491942	96.38681218
636	41346.34	1706435	55868.5	0.000558	0.01517	3.556526	96.71383295
696	33512.85	1740238	56603.03	0.000446	0.01527	2.845297	97.34974328

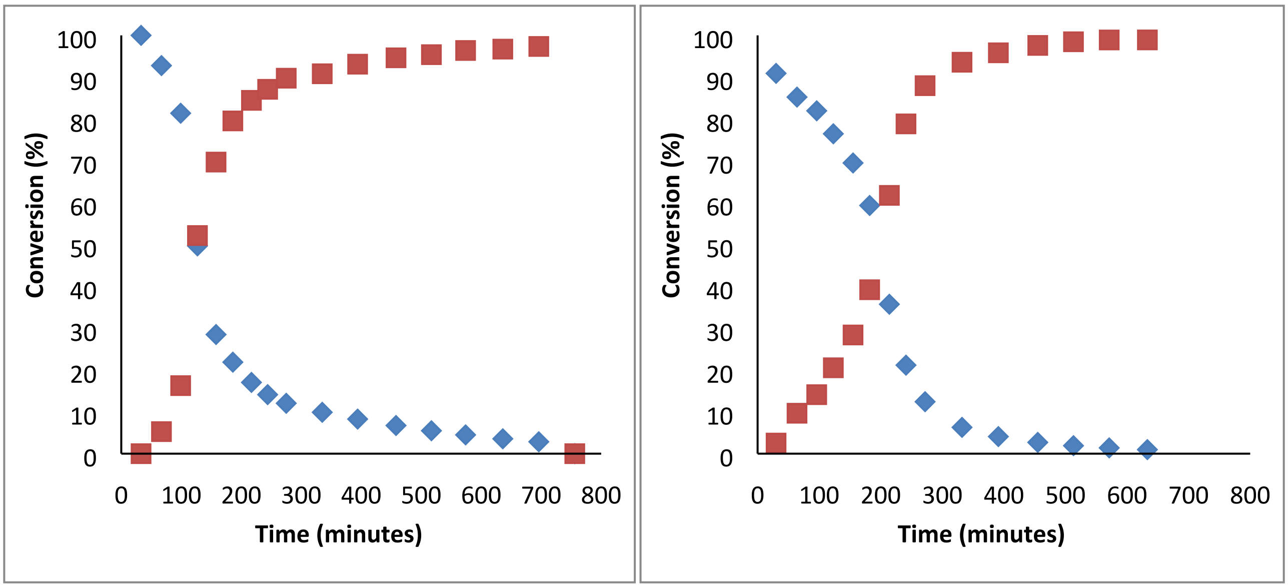

Figure S18. Conversion *vs* time (a) with 0.5 eq. of **5** and (b) under standard conditions

5.3.8 Reaction with 1.0 eq of H_2O at the beginning

	11 - Cs_2CO_3 1.0 eq H_2O						
**Time (mins)**	PA ArI	PA Prod	PA Std	[SM]	[Prod]	ArI %	Prod Form %
32	980636.7	54478.95	62448.46	0.011837	0.000433	91.930558	3.365041774
69	816739.5	126456.7	55584.89	0.011076	0.00113	86.020176	8.775434455
98	749461.4	181962.4	53393.05	0.010581	0.001693	82.17468	13.14560172
129	684268.9	239412.8	51481.43	0.010019	0.00231	77.812563	17.9382562
157	633410.4	290698.7	49833.81	0.009581	0.002897	74.410568	22.50103244
188	641972.5	384752.3	54180.11	0.008932	0.003527	69.36654	27.39205982
216	461594.2	351018.1	40877.43	0.008512	0.004265	66.107436	33.12298044
247	439286.1	399724.5	42749.02	0.007746	0.004644	60.158184	36.06766261
274	376747.4	432595	37988.81	0.007476	0.005656	58.058808	43.92474669
334	158115.5	809519.2	40555.78	0.002939	0.009914	22.824176	76.99416116
393	61986.28	968976.3	41357.68	0.00113	0.011637	8.7743086	90.37335456
457	25221.56	915894.7	36805.34	0.000517	0.01236	4.0117559	95.98824405
515	16041.85	1116561	44066.72	0.000274	0.012585	2.1311648	97.73612517
573	7410.274	947561.5	36864.27	0.000152	0.012766	1.1767985	99.14828248
639	4603.591	1097073	42911.34	8.09E-05	0.012698	0.6280556	98.61591688

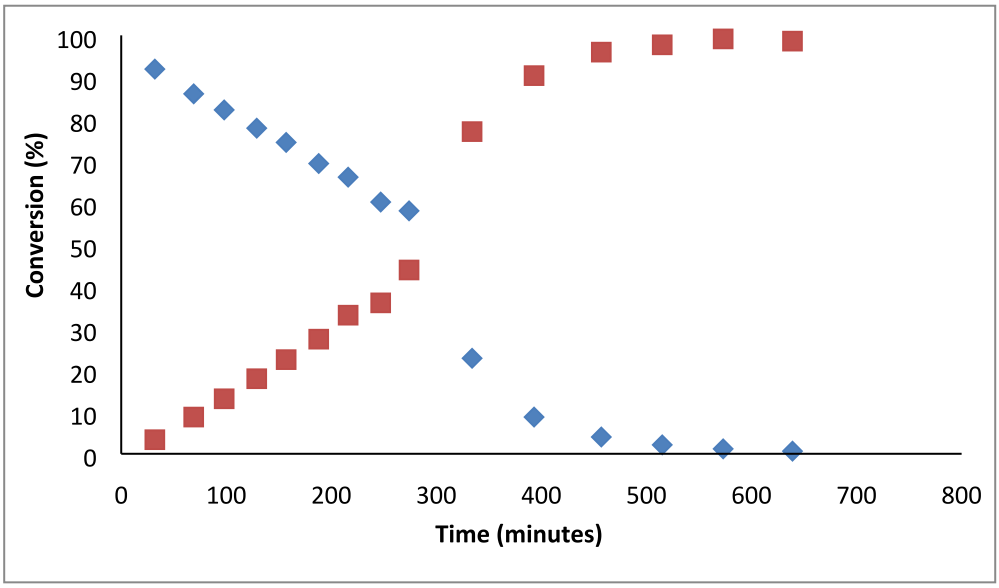

Figure S19. Conversion *vs* time in the presence of 1 eq. of H_2O

5.4 Kinetic data with L^2H/K_3PO_4

Protocol described in section 5.1 was followed. The detailed quantities of reagents are summarised below.

	**MW ($g.mol^{-1}$)**	**Density ($g.mL^{-1}$)**	**Standard**	**0.5x [CuI/L^1]**	**2x [4]**	**Std Duplicate**	**0.5x CuI**	**2x [L^1H]**	**2x K_3PO_4**
CuI (mg)	190.44		9.5	4.8	9.5	9.5	4.7	9.5	9.5
**L^2**H (mg)	193.24		19.3	9.7	19.3	19.3	19.3	38.6	19.3
1,1'-Biphenyl (mg)	154.21		7.7	7.7	7.7	7.7	7.7	7.7	7.7
K_3PO_4 (mg)	325.82		244	244	244	244	244	244	488
DMF (µl)			2500	2500	2500	2500	2500	2500	2500
**1** (mg)	234.03		117	117	117	117	117	117	117
**4** (µl)	85.15	1.116	57	57	114	57	57	57	57

5.4.1 Reaction under standard conditions

	**10 - K_3PO_4 Std**						
**Time (mins)**	PA ArI	PA Prod	PA Std	[SM]	[Prod]	ArI %	Prod Form %
50	832326.2	93074.82	56224.53	0.011159	0.000822	98.6	7.3
112	1083567	506569.4	86675.3	0.009424	0.002903	83.2	25.6
173	536204.2	468594.1	52788.2	0.007657	0.004409	67.6	38.9
242	437313.6	575471.9	51004.6	0.006463	0.005604	57.1	49.5
300	844376.4	1372924	105182.8	0.006051	0.006483	53.5	57.3
363	702414.7	1404938	98290.64	0.005387	0.007099	47.6	62.7
420	359939.1	868231.1	57427.54	0.004725	0.007509	41.7	66.3
482	316525.6	891033.7	55912.07	0.004267	0.007915	37.7	69.9
539	297969	924630.4	56243.88	0.003994	0.008165	35.3	72.1
602	295750.2	1038246	60669.82	0.003675	0.0085	32.5	75.1
622	258655.1	930763	54605.9	0.003571	0.008466	31.5	74.8

662	259560.7	988933.8	56732.67	0.003449	0.008658	30.5	76.5
720	219565.9	927262.1	52152.67	0.003174	0.008831	28.0	78.0
774	229118.1	1030883	57504.52	0.003003	0.008904	26.5	78.7

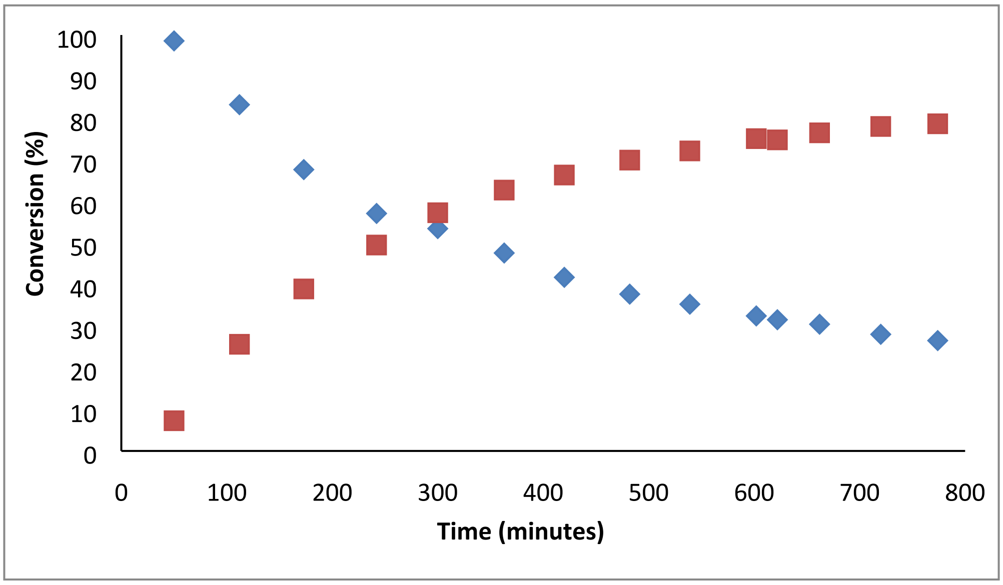

Figure S20. Conversion *vs* time under standard conditions

5.4.2 Repeat of reaction under standard conditions

	11 - K_3PO_4 Std repeat						
**Time (mins)**	PA ArI	PA Prod	PA Std	[SM]	[Prod]	ArI %	Prod Form %
51	828865.7	107468.4	57556.79	0.010855	0.000927	95.9	8.2
112	622736.6	319247.6	52802.93	0.00889	0.003003	78.5	26.5
173	538756.7	496317.3	54597.42	0.007438	0.004515	65.7	39.9
243	546897.3	736080.7	64485.01	0.006393	0.005669	56.5	50.1
300	618573.5	1042943	80361.22	0.005802	0.006446	51.3	56.9
363	567948.2	1163954	81985.56	0.005222	0.007051	46.1	62.3
420	310109.4	745863.7	50170.19	0.004659	0.007384	41.2	65.2
482	259074.8	734641.5	46808.73	0.004172	0.007795	36.9	68.9
539	295858.7	925900.9	56942.64	0.003917	0.008076	34.6	71.3
602	228828.7	819406.2	48387.98	0.003565	0.008411	31.5	74.3
622	242222.2	893004.2	52468.78	0.00348	0.008453	30.7	74.7
662	282122.6	1086148	62527.93	0.003401	0.008628	30.0	76.2
720	215202.7	898119.6	51440.98	0.003154	0.008672	27.9	76.6
774	224170.4	1007039	56403.85	0.002996	0.008868	26.5	78.3

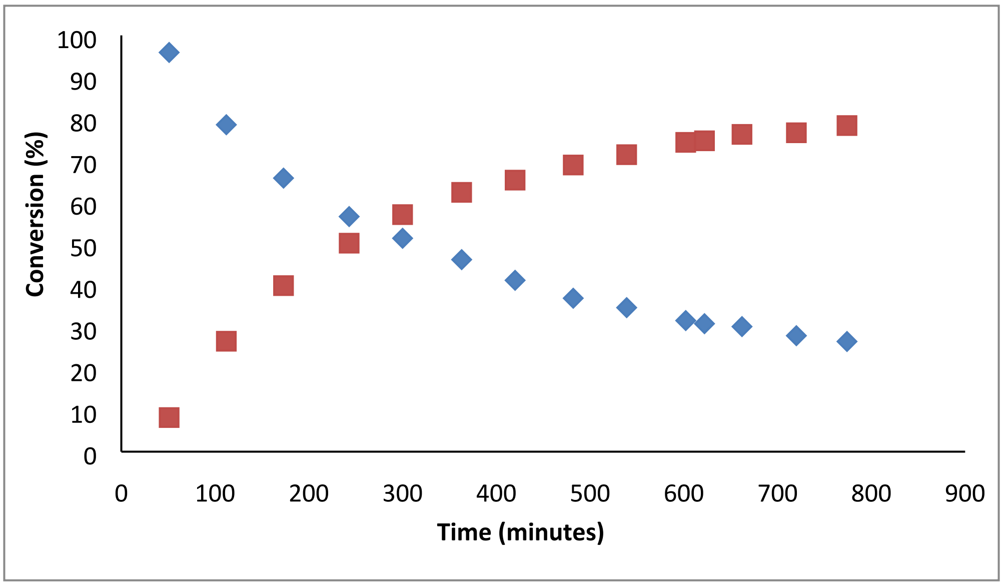

Figure S21. Conversion *vs* time under standard conditions (repeat)

5.4.3 Reaction with 0.5 x $[CuI]_0$ and 0.5 x $[L^2H]_0$

	12 - 0.5x [CuI/L]						
**Time (mins)**	PA ArI	PA Prod	PA Std	[SM]	[Prod]	ArI %	Prod Form %
51	831365.4	15285.59	55791.87	0.011233	0.000136	99.2	1.2
112	1250241	52067.97	84983.67	0.01109	0.000304	98.0	2.7
173	727560.7	47407.76	51426	0.010665	0.000458	94.2	4.0
243	716555.1	63413.94	51474.53	0.010493	0.000612	92.7	5.4
300	1439793	162612.3	101130.2	0.010732	0.000799	94.8	7.1
363	713530.2	96291.08	52546.56	0.010236	0.00091	90.4	8.0
420	689202.1	110539.3	51613.7	0.010066	0.001064	88.9	9.4
482	695442	128090.6	52846.46	0.00992	0.001204	87.6	10.6
540	743596.3	154324.4	59572.53	0.009409	0.001287	83.1	11.4
602	755952.3	179783.9	58894.47	0.009676	0.001516	85.5	13.4
622	748864.8	185480.9	58731.95	0.009611	0.001569	84.9	13.9
662	894931.2	239562.5	70360.11	0.009588	0.001691	84.7	14.9
720	723825	212114.3	58586.88	0.009313	0.001798	82.3	15.9
774	651044.6	209610.1	53851.51	0.009113	0.001933	80.5	17.1
834	666658	237356.9	56035.19	0.008968	0.002104	79.2	18.6

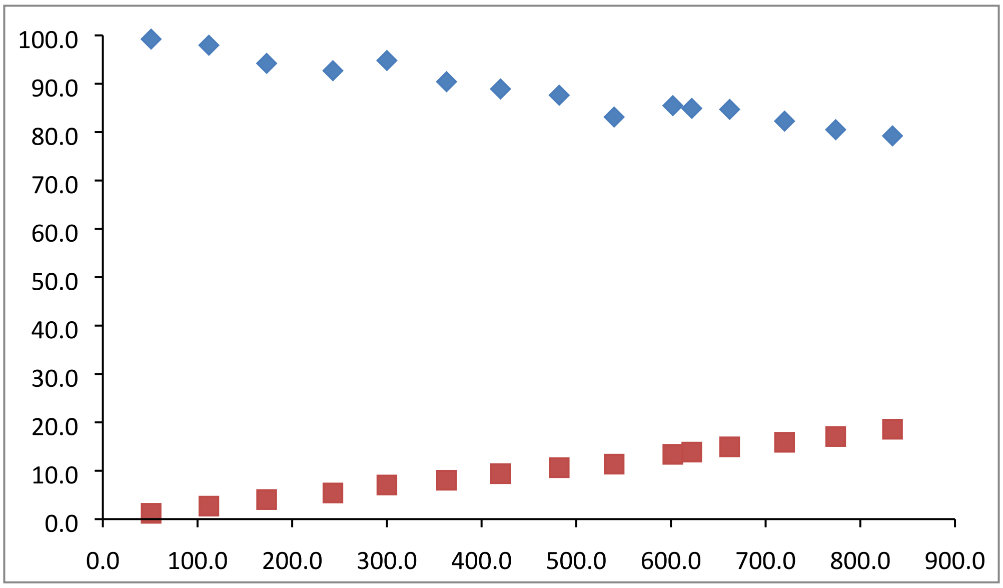

Figure S22. Conversion *vs* time at ½ x [CuI] and [**L**[1]H]

5.4.4 Reaction with 2 x $[L^2H]_0$

	13 - 2x [Ligand]						
**Time (mins)**	PA ArI	PA Prod	PA Std	[SM]	[Prod]	ArI %	Prod Form %
51	743267.8	215545.5	61703.72	0.00908	0.001735	80.2	15.3
112	574745.2	419893.5	58501.2	0.007406	0.003565	65.4	31.5
173	493400.1	544480.5	57890.17	0.006425	0.004671	56.8	41.3
243	438624	659176.8	58974.67	0.005606	0.005551	49.5	49.0
301	717778.3	1272141	102517.4	0.005278	0.006163	46.6	54.4
363	681146.7	1426335	106561.6	0.004818	0.006648	42.6	58.7
420	353960.8	881610.6	62577.32	0.004264	0.006997	37.7	61.8
482	447450.9	1237535	83782.04	0.004026	0.007336	35.6	64.8
540	285598.9	895244.3	58747.74	0.003665	0.007569	32.4	66.9
603	276763.6	952953.9	60805.26	0.003431	0.007784	30.3	68.8
622	267237.1	944561.5	59967.42	0.003359	0.007823	29.7	69.1
662	214400.7	812531.1	50740.39	0.003185	0.007953	28.1	70.3
721	207805.9	853448	52273.61	0.002997	0.008109	26.5	71.6
774	235108.3	1001362	60555.71	0.002927	0.008213	25.9	72.6

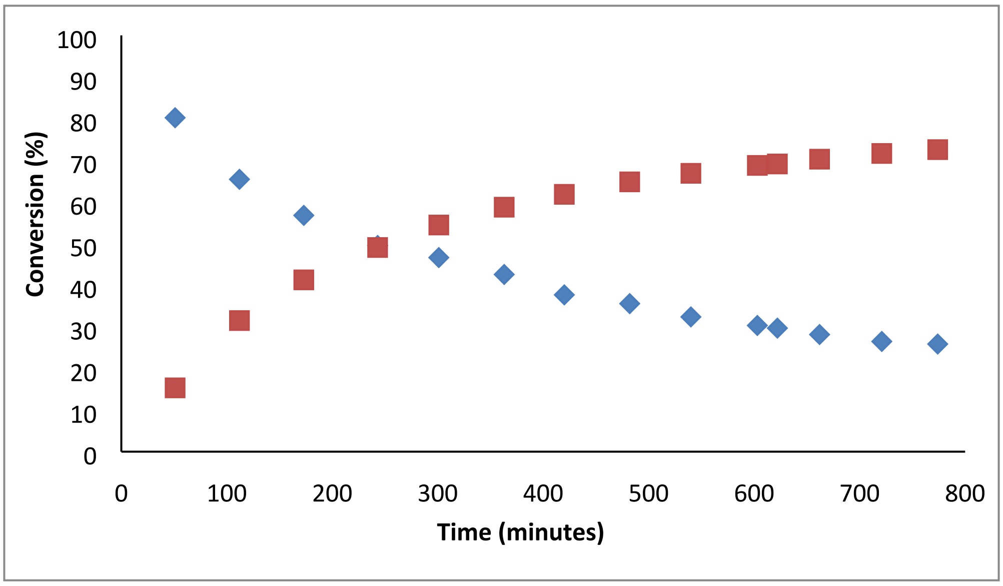

Figure S23. Conversion *vs* time at 2 x [CuI] and [**L**[1]H]

5.4.5 Reaction with 0.5 x $[L^2H]_0$

	**14 - 0.5x [Ligand]**						
**Time (mins)**	PA ArI	PA Prod	PA Std	[SM]	[Prod]	ArI %	Prod Form %
51	1381208	47337.94	89008.24	0.011697	0.000264	103.3	2.3
112	1140044	102487.1	76352.37	0.011255	0.000667	99.4	5.9
173	858005.1	163705	61671.29	0.010487	0.001318	92.6	11.6
243	659901.2	308394.9	55229.58	0.009007	0.002773	79.6	24.5
301	1347611	1114004	127165.1	0.007988	0.004351	70.6	38.4
364	432781.4	526612.5	50091.76	0.006513	0.005221	57.5	46.1
420	420064	679791.7	55493.56	0.005706	0.006084	50.4	53.7
483	371328.5	759560.6	55784.94	0.005018	0.006763	44.3	59.7
540	331265.4	817441	55675.72	0.004485	0.007292	39.6	64.4
603	299267	871479.8	56134.4	0.004019	0.007711	35.5	68.1
623	318385.3	968847.5	61307.83	0.003915	0.007849	34.6	69.3
663	250623.4	853109.6	52291.96	0.003613	0.008103	31.9	71.6
721	259571.8	995108.5	59203.3	0.003305	0.008348	29.2	73.7
775	223251.3	943141.5	54818.82	0.00307	0.008545	27.1	75.5
834	221073.3	1031762	58603.17	0.002844	0.008744	25.1	77.2

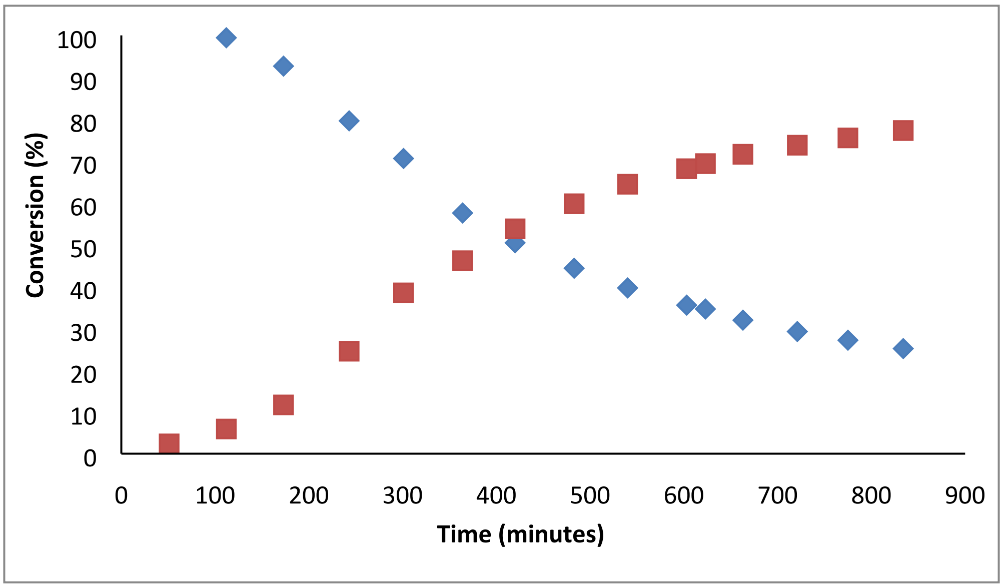

Figure S 24. Conversion *vs* time at ½ x [L[1]H]

5.4.6 Reaction with 2 x K_3PO_4

	2 x [K_3PO_4]						
**Time (mins)**	PA ArI	PA Prod	PA Std	[SM]	[Prod]	ArI %	Prod Form %
51	793376	24602.04	56105.54	0.010659	0.000218	94.2	1.9
113	841502.3	57108.17	60483.55	0.010488	0.000469	92.6	4.1
174	784065.9	82299.16	58057.55	0.01018	0.000704	89.9	6.2
244	676057.1	100953.6	51462.76	0.009903	0.000974	87.5	8.6
301	649321.5	120757.2	50605.91	0.009672	0.001185	85.4	10.5
364	1317189	303659.1	101453.8	0.009787	0.001487	86.5	13.1
421	617657.1	165451.3	50472.34	0.009225	0.001628	81.5	14.4
483	673499.1	213116.2	56107.71	0.009048	0.001887	79.9	16.7
540	605689	215824.1	51904.72	0.008796	0.002065	77.7	18.2
603	591231.2	240354.2	51981.47	0.008574	0.002297	75.7	20.3
623	464994.9	196600.1	41746.9	0.008396	0.002339	74.2	20.7
663	612322.4	279472.7	55248.23	0.008355	0.002512	73.8	22.2
721	659479.3	329015.8	60808.72	0.008175	0.002687	72.2	23.7
775	598169.1	327601.4	56850.52	0.007931	0.002862	70.1	25.3
834	569422.7	344306.7	55684.46	0.007708	0.003071	68.1	27.1

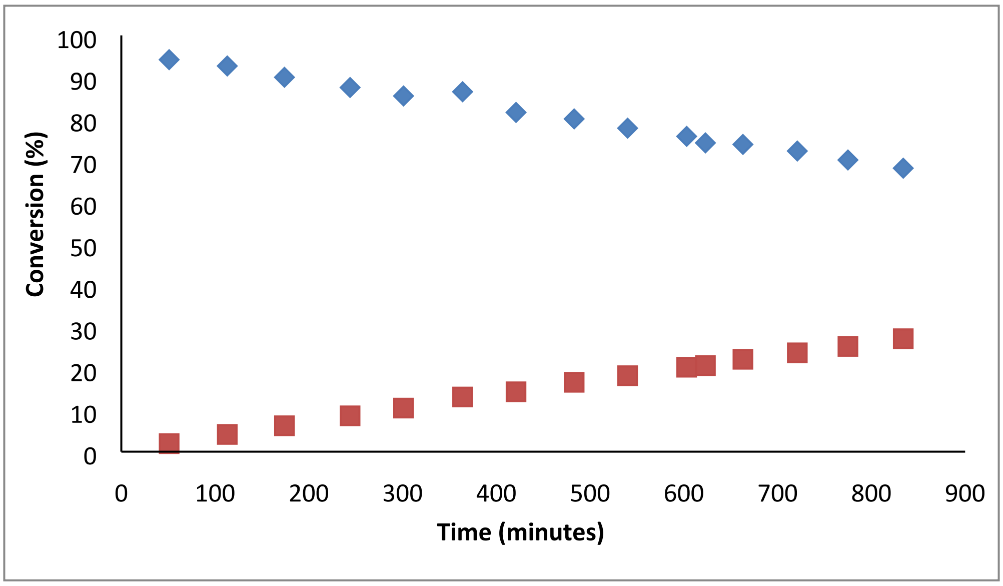

Figure S25. Conversion *vs* time at 2 x K_3PO_4

5.4.7 Reaction with 2 x $[4]_0$

	2x [Amide]						
**Time (mins)**	PA ArI	PA Prod	PA Std	[SM]	[Prod]	ArI %	Prod Form %
52	764835.4	28043.44	52765.33	0.010926	0.000264	96.5	2.3
113	725752.6	63779.2	51730.08	0.010576	0.000612	93.4	5.4
174	659249.9	95482.65	48751.91	0.010193	0.000973	90.0	8.6
244	949262	226673	72461.42	0.009875	0.001554	87.2	13.7
302	770540.2	274661.6	63126.34	0.009201	0.002161	81.3	19.1
364	605539.9	334609.9	55197.42	0.00827	0.003011	73.1	26.6
421	553979.6	437704.8	56278.44	0.00742	0.003863	65.5	34.1
483	450503.4	499282.5	52249.56	0.006499	0.004746	57.4	41.9
541	433399.1	630050.6	56835.14	0.005748	0.005506	50.8	48.6
604	405054.6	762469	60781.93	0.005023	0.00623	44.4	55.0
624	359090.5	731685.3	56718.33	0.004772	0.006407	42.2	56.6
663	373673.3	882732.8	64182.55	0.004389	0.006831	38.8	60.3
722	228225.1	666694.5	45897.11	0.003748	0.007215	33.1	63.7
775	250217.6	881425	56840.74	0.003318	0.007702	29.3	68.0
835	198948.2	844650.3	52086.95	0.002879	0.008054	25.4	71.1

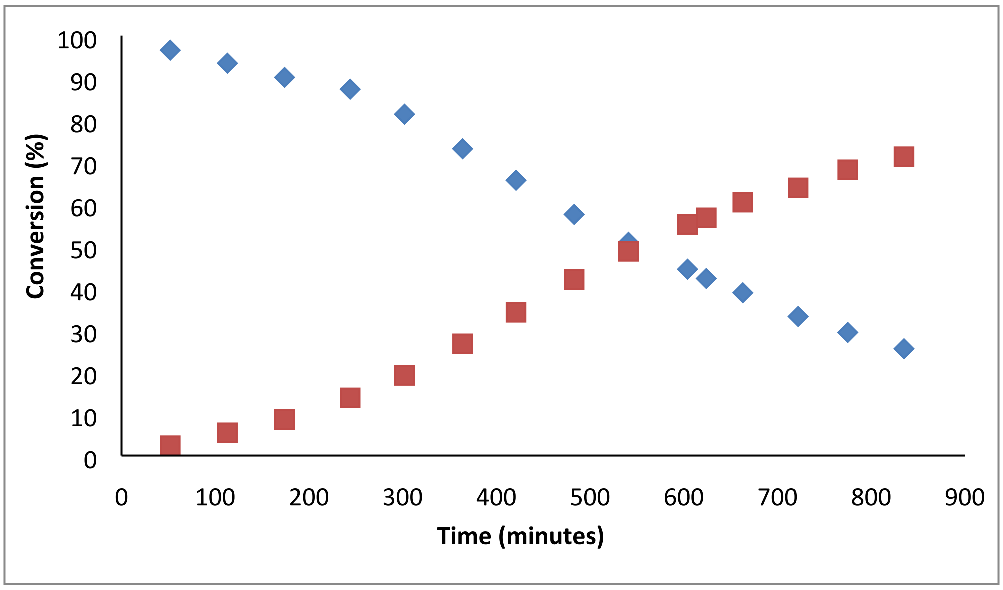

Figure S26. Conversion *vs* time at 2 x [**4**]

5.5 Reaction with L^3/Cs_2CO_3

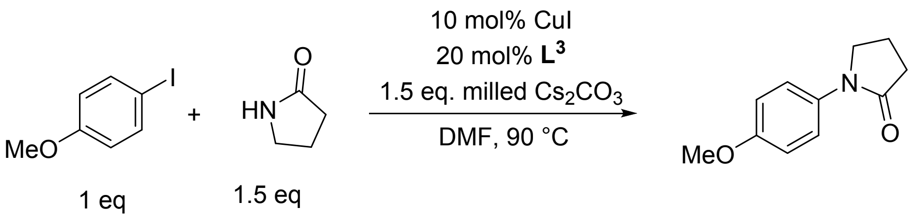

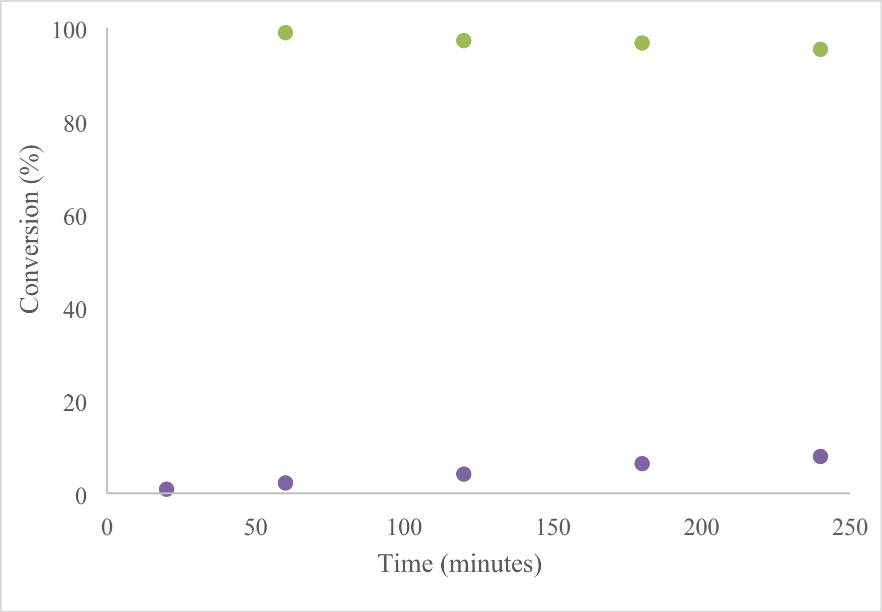

Figure S27. Reaction profile using $\mathbf{L}^3/Cs_2CO_3$ combination

A reaction protocol similar to one described in section 5.1 was employed, with $\mathbf{L}^3$ replacing $\mathbf{L}^1$H. When using oven dried, milled Cs_2CO_3, the above reaction was extremely slow, with kinetic monitoring over the first 4 hours showing up to 8 % product. After 48 hours, GC analysis showed 45% conversion to product. In a repeat of the experiment without sampling, conversion to product reached 57 %.

6. Reaction with additional halide salts

CuI (19 mg, 0.1 mmol, 10 mol%), 1,10-phenanthroline **L**[3] (36 mg, 0.2 mmol), Cs_2CO_3 (489 mg, 1.5 mmol), 4-iodoansiole **1** (234 mg, 1 mmol) and the halide salt (1 mmol, 1 eq.) were added to a flame dried Schlenk flask. The vessel was evacuated and backfilled with argon gas three times before the 2-pyrrolidinone **4** (114 µl, 1.5 mmol) and degassed, anhydrous DMF (5 mL) were added. The reaction was stirred at 90 °C under a gentle flow of argon for 14 hours. Upon completion, 100 µl of the reaction mixture was diluted to 1.5 ml with MeCN and analysed by GC (calibration data in section 10.1).

Additive	Conversion to product 5 (%)	Side products
None	87	
NaI	14	
NaBr	14	4-Bromoanisole (5%)
NaCl	26	4-Chloroanisole (trace)
CsI	32	

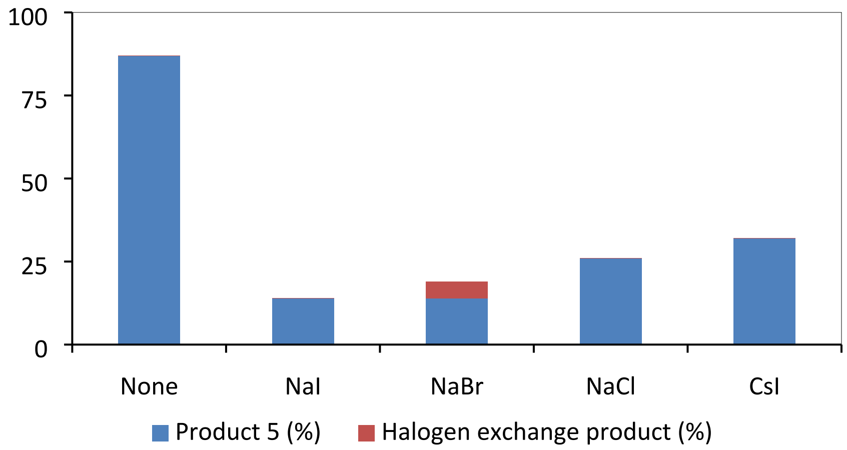

Figure S28. Conversion at 14 hours in the presence of halide salts

7. EPR experiments

7.1 *In situ* EPR experiment

Reaction protocol from section 5.1 was scaled for 2.5 g of 4-iodoanisole **1** (10.7 mmol), with one difference: CuI was added last to the pre-equilibrated reaction mixture containing all other components to enable EPR background measurements. The reaction was performed under argon using in a jacketed glass reactor at 80 °C and the solution (with *in situ* filtration) was circulated through the EPR cavity using an FEP tube (ID 0.8 mm) using a Milligat pump at a flow rate of 3.0 mL/min. EPR spectra were recorded on a Brücker ELEXYS spectrometer at room temperature in X-band with a microwave power of 6.9 mW, a modulation frequency of 100 kHz and modulation amplitude of up to 5 G.

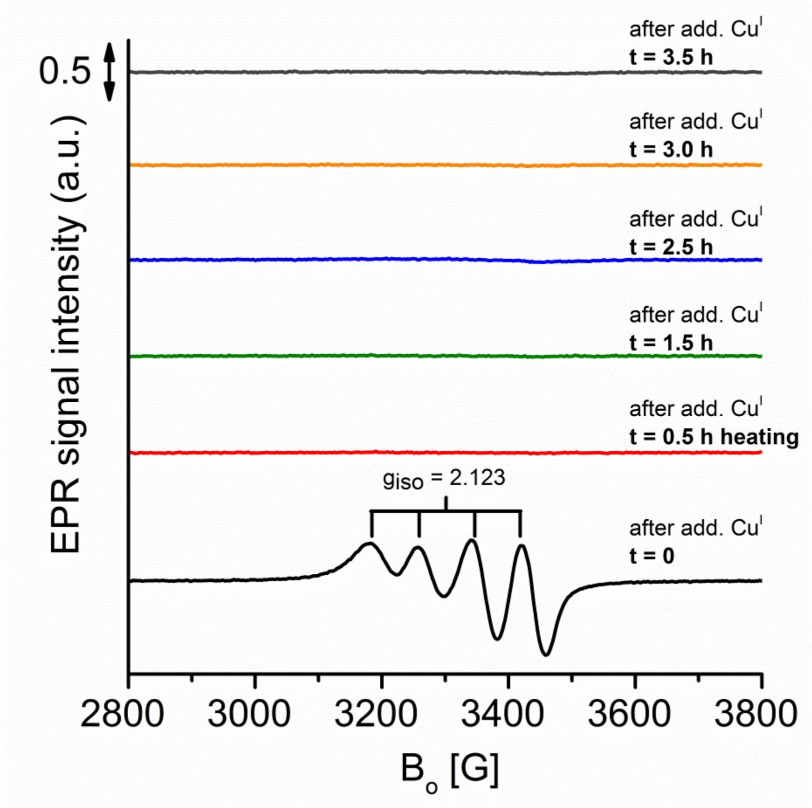

Figure S29. *In situ* EPR measurements over the course of the experiment. Immediately after addition of CuI an isotropic Cu(II) signal emerged, which vanished after about 5-10 min of heating. The Cu(II) signal was not observed afterwards

7.2 CuI/L[1]H/DMF experiment

Sample was prepared using a standard EPR tube in a glovebox with degassed DMF and measured using the same equipment as described above. The sample was cooled with liquid nitrogen during this measurement.

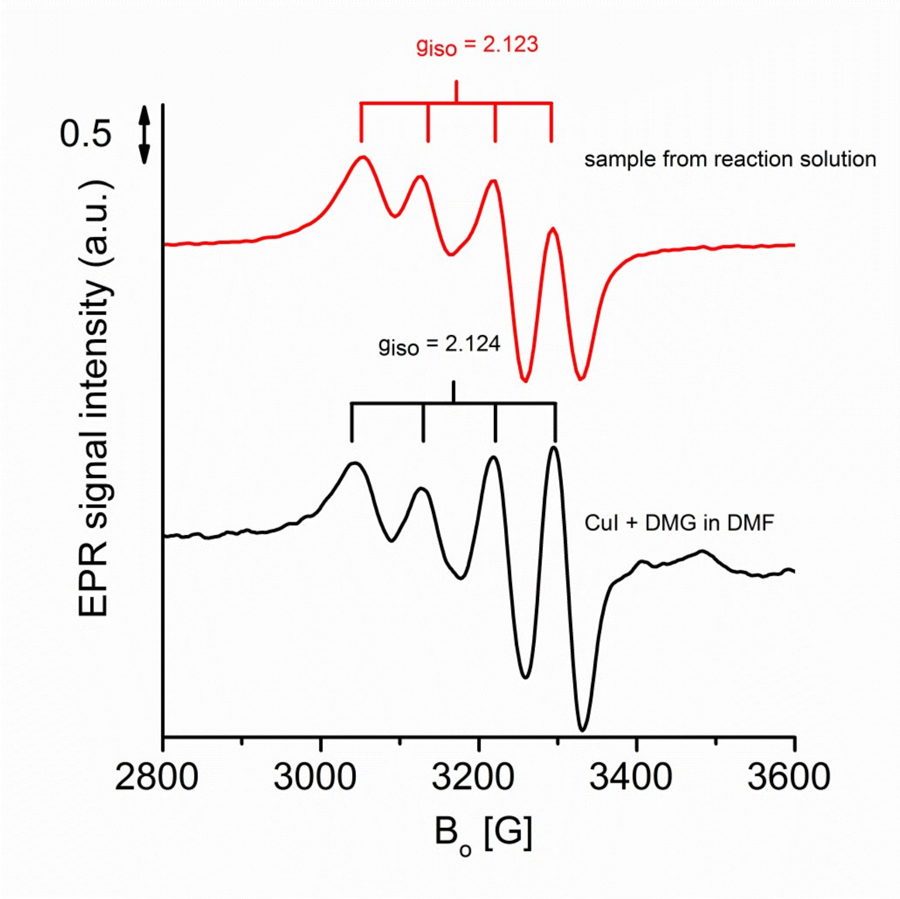

Figure S30. Normalized EPR spectra of Cu(II) signal observed during reaction (red) and CuII signal observed when CuI is added to a solution of 2.0 equivs of DMG in DMF in Glovebox (black).

Slight line broadening is observed in CuII signal of reaction solution (red graph) which lead to loss in intensity at high field peaks. These effects could be caused by a slower tumbling rate due to interaction with other reaction components (substrate, base etc.) which were absent in the CuI+DMG experiment (black).

8. Characterization of commercial Cs_2CO_3 samples

8.1 Sample preparation

Bases were analysed by SEM either fresh from the bottle or following drying in a 75 ºC oven when specified. Samples were stored under argon prior to analysis.

8.2 SEM/EDX experiments

All samples were mounted in powder form, under exclusion of ambient moisture, on SEM sample stubs using adhesive carbon film. The samples were then coated with a thin layer of iridium using a Cressington 208HR sputter coater. Samples were imaged using an FEI Nova NanoSEM 450 operating typically at 3 kV. The gun voltage was increased to 10 keV to collect EDX point spectra.

8.2.1 Sigma Aldrich, 99 %. Lot # BCBP3311V. PCode 101550748

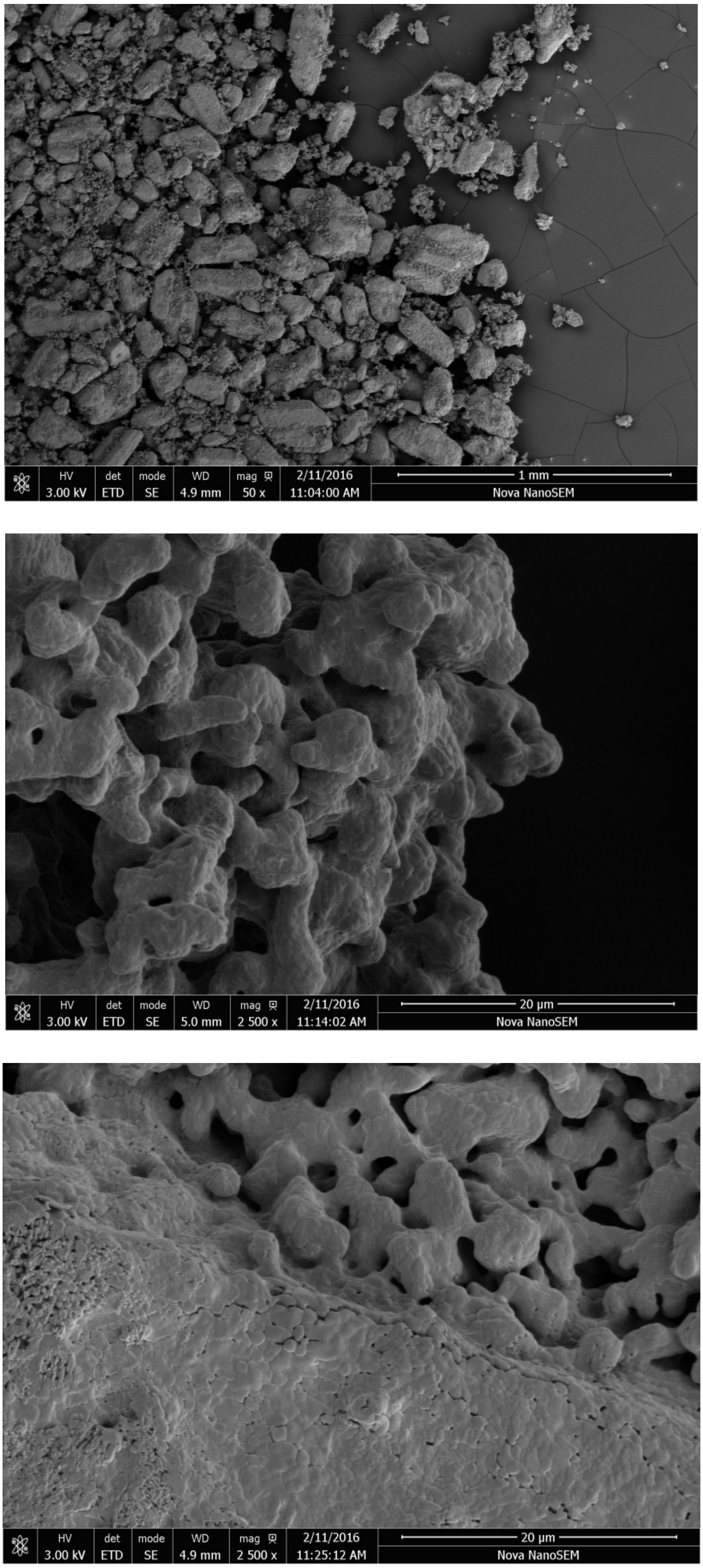

Figure S31. SEM pictures of **Base 2**

8.2.2 Acros Organics, 99.5 %. Lot # A0359643. Code 192041000

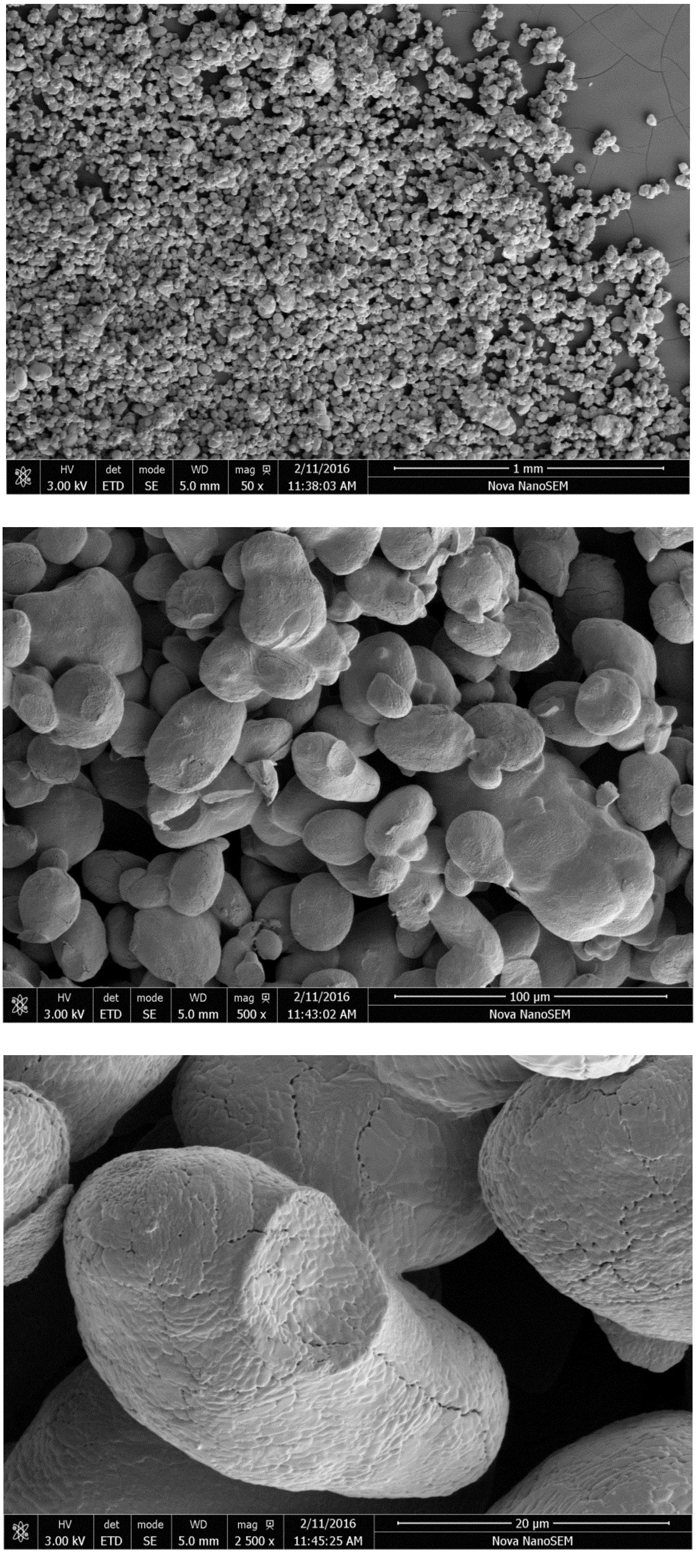

Figure S32. SEM pictures of **Base 3**

8.2.3 Chemetall, Batch No. 27091B032

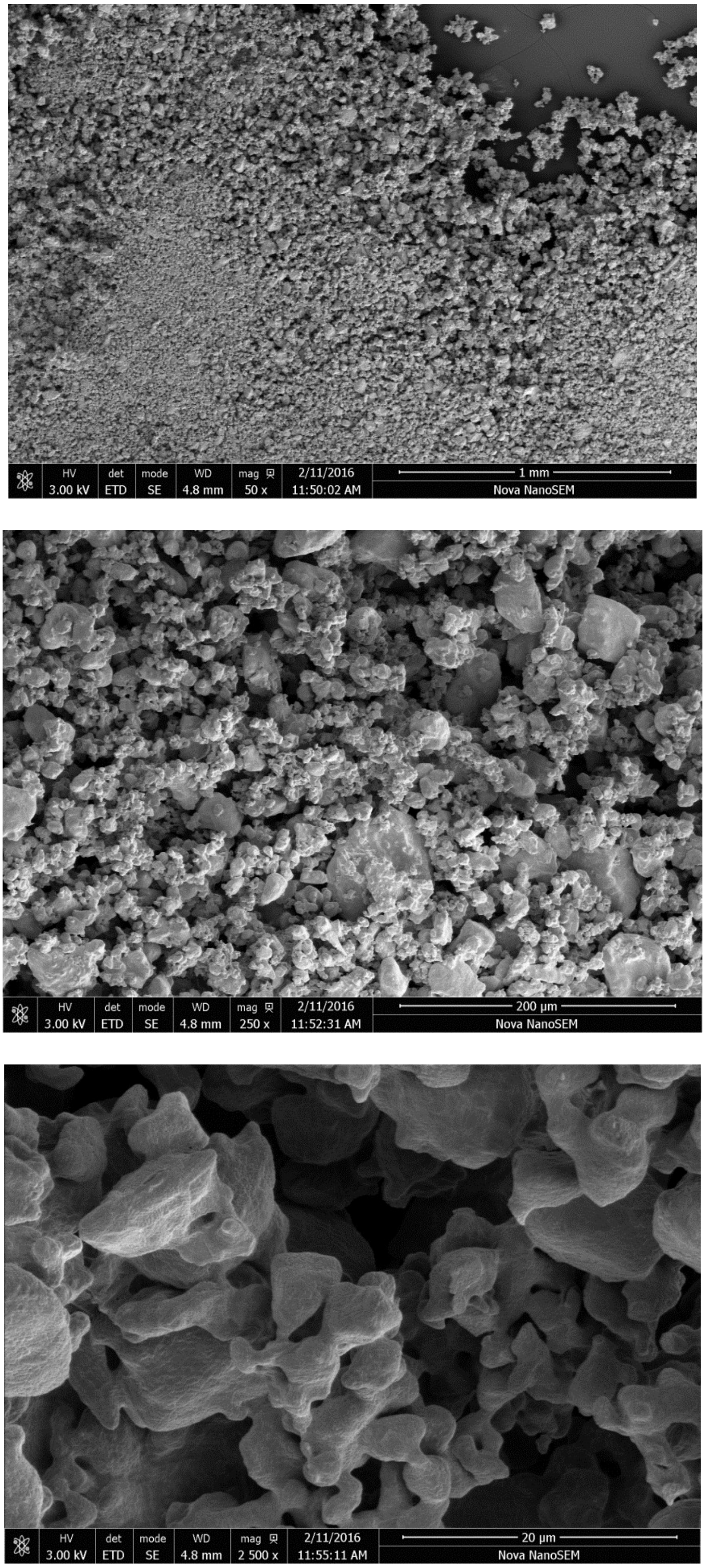

Figure S33. SEM pictures of **Base 1**

8.3 Surface area measurements

Nitrogen adsorption measurements indicate that all three base powders have very small specific surface areas (<1 m^2/g). Nitrogen adsorption measurements are not suited to accurately quantify such low specific surface areas, so a comparison between the three bases using this method is not feasible.

9. Characterisation of solid during and after reaction

Reactions were performed as outlined in section 5.1. Following completion of the reaction, the solution stopped stirring and was removed *via* syringe at 90 °C, the solid was then washed three times with hot, anhydrous Et_2O and evaporated to dryness. The dried solid was stored under argon until analysis was performed.

9.1 SEM/EDX experiments

9.1.1 Base Isolated Post-reaction – Chemmetal Cs_2CO_3

In order to probe the link the morphology and surface area of the bases with their kinetic behavior, solid samples were recovered from the reaction mixtures using **Base1** and **Base2** at 2.5 and 5.0 hours. SEM pictures of these are shown in Figure S34. Chemical speciation was achieved by a combination of EDX data and power X-ray diffraction (Figure S37). Comparing Figure S34a-d showed that, as the reaction progresses, bright cubic crystals of CsI (in SEM pictures, Figure S34e), assigned based on EDX characterization (Figure S35-S37), accumulates on the solids. More importantly, the soft-edge structure of Cs_2CO_3 gave way to a crystalline material which was identified by EDX to contain Cs, C, and O. Powder X-ray diffraction data of the sample recovered from reaction using **Base2** at 5 hours indicated that these crystals are $CsHCO_3$ (Figure S34e). No diffraction signal for Cs_2CO_3 was detected for this sample. Thus, during the reaction, the morphology all Cs_2CO_3 samples changes toward a common final state. The difference in the initial rates of reaction using **Base1**, **Base2** and **Base3** must therefore originated from their initial states. We hypothesized that the difference in surface area, and hence their rates of dissolution in DMF,[6] are the main reason for the observed induction period with **Base1**.

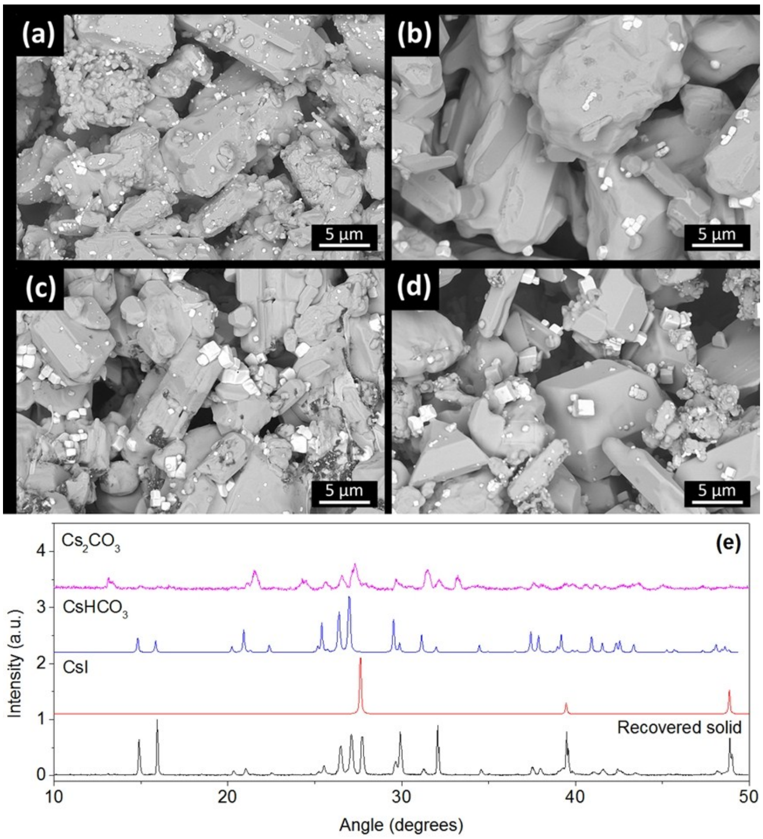

Figure S34. SEM images of inorganic solids from the reaction mixture using (a) Base1 at 2.5 hour; (b) Base2 at 2.5 hours; (c) Base1 at 5 hours; (d) Base2 at 5 hours of reaction time; (e) Power diffraction patterns of the recovered solid (Base2) after 5 hours.

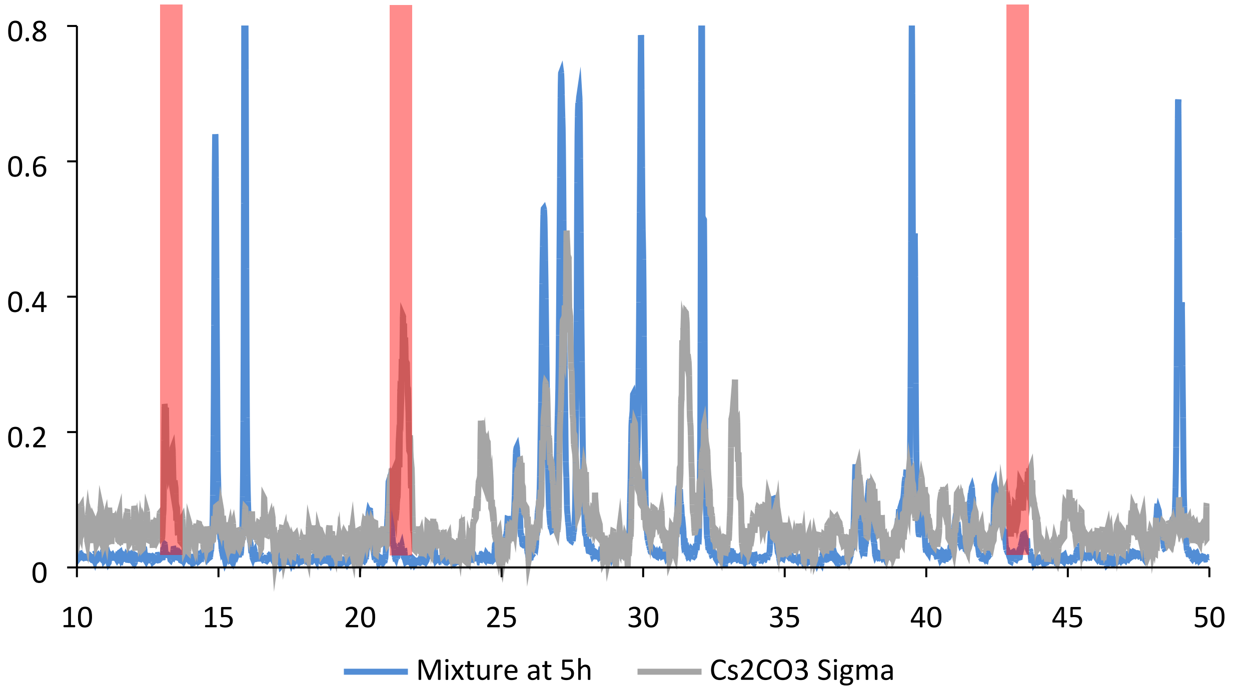

Figure S35. Comparison between the powder diffraction patterns of Base1 at 5 hours and a fresh sample of Base1. Some observable common peaks are highlighted in red.

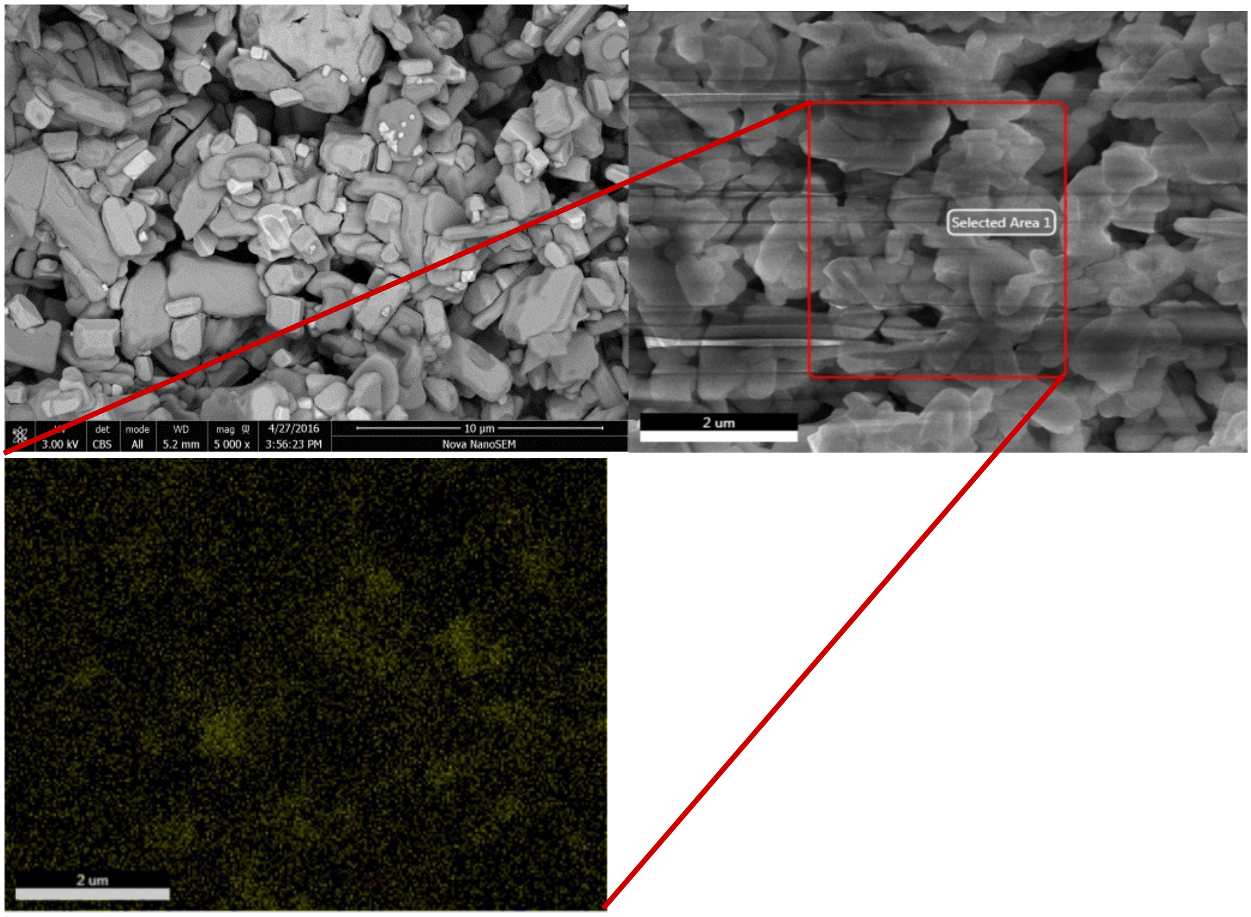

Figure S36. SEM/EDX results of isolated base post reaction: (a) SEM imaging of bulk material post-reaction, low magnification; (b) zoomed in picture of the region of which EDX spectra relates to; and (c) Iodine distribution.

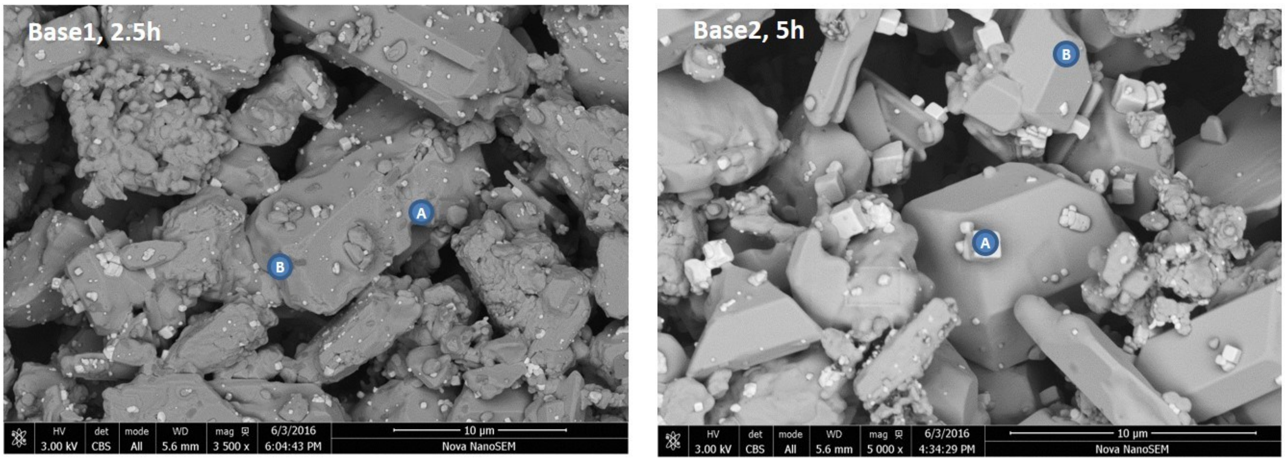

Spot	Cs	O	I	Cu
A	22at%	72at%	5at%	1at%
B	21at%	78at%	0.5at%	0.5at%

Spot	Cs	O	I	Cu
A	46at%	22at%	31.5at%	0.5at%
B	69at%	27at%	3at%	1at%

Figure S37. Elemental compositions of recovered **Base1** and **Base2**, as probed locally by EDX point spectra in two different areas of each sample (indicated by Spot A and Spot B in the SEM images).

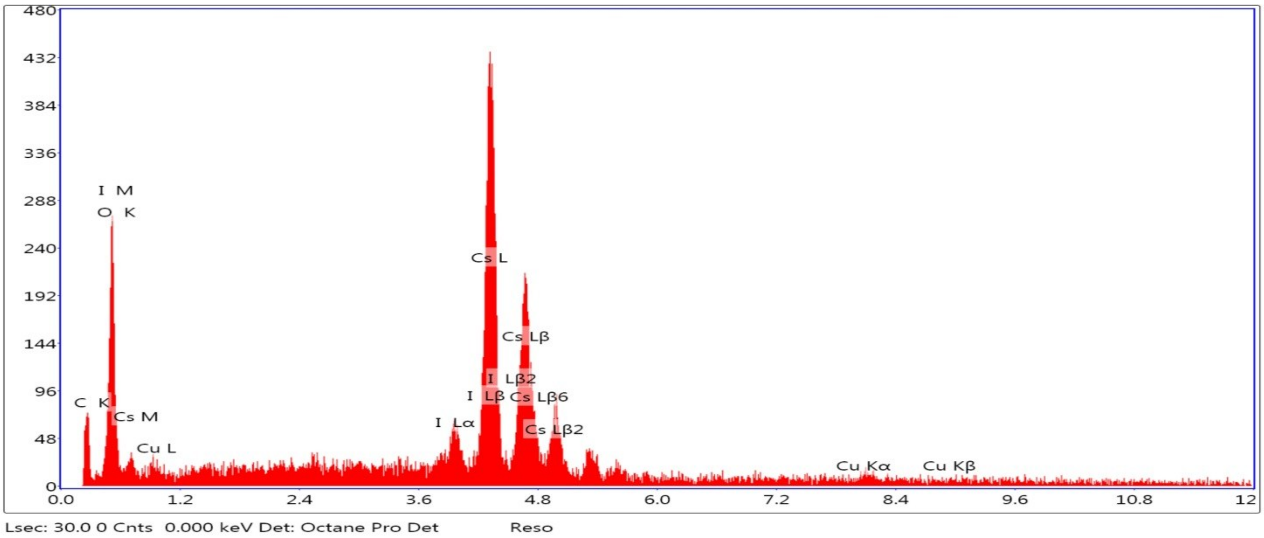

Figure S38. EDX spectrum of the bulk material

9.2 XRD measurements

X-Ray powder diffraction data was collected on a Bruker D2Phaser Diffractometer. Powdered samples were mounted on a silicon wafer by evenly distributing the powder over the wafer. All samples were rotated during data collection to ensure more homogeneous (i.e. isotropic) diffraction patterns. Data collection was carried out at room temperature, using Cu Kα radiation (λ = 1.54184 Å). Diffraction patterns were recorded in step-scan mode with a step size of (2θ) 0.7°, from (2θ) 5° to 55° (30 secs per step) using a 0.1 or 0.6mm divergent slit. Samples showed no significant evidence of degradation within the X-ray beam. The software package Diffrac.Suite Eva14 was used to process the experimental powder X-ray diffraction data collected, and Mercury 3.3 was used to simulate the powder diffraction pattern of the single crystal structures taken from literature.

10. Base-less coupling reaction

10.1 GC Calibrations

1,1'-Biphenyl used as an internal standard (IS).

**[1] (M)**	**0.0167**	**0.0133**	**0.0067**	**0.0033**	**0.0013**
**Area 1**	1003.5	788.1	400	201.8	76.3
**Area IS – 0.000133 M**	159.6	157.6	159	158.1	157.2
**Area 1/IS**	6.29	5.00	2.52	1.28	0.49

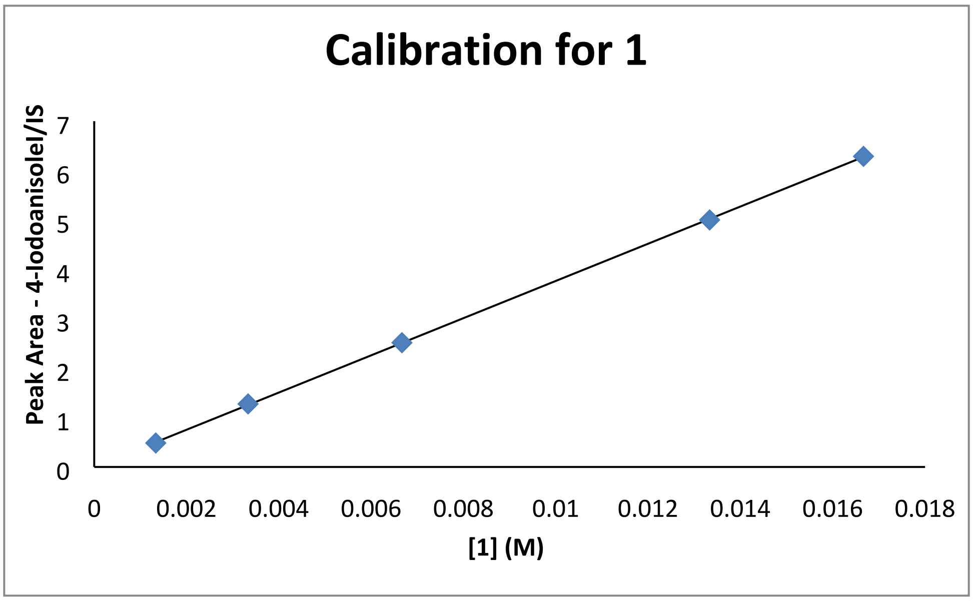

Product **5** = 1-(4-methoxyphenyl)pyrrolidin-2-one

[5]	0.0167	0.0133	0.0067	0.0033	0.0013
**Area 5**	1272,4	1002.7	491.8	231.7	83.8
**Area IS – 0.000133 M**	127	124.3	122	119.5	117.2
**Area 5/IS**	10.02	8.07	4.03	1.94	0.72

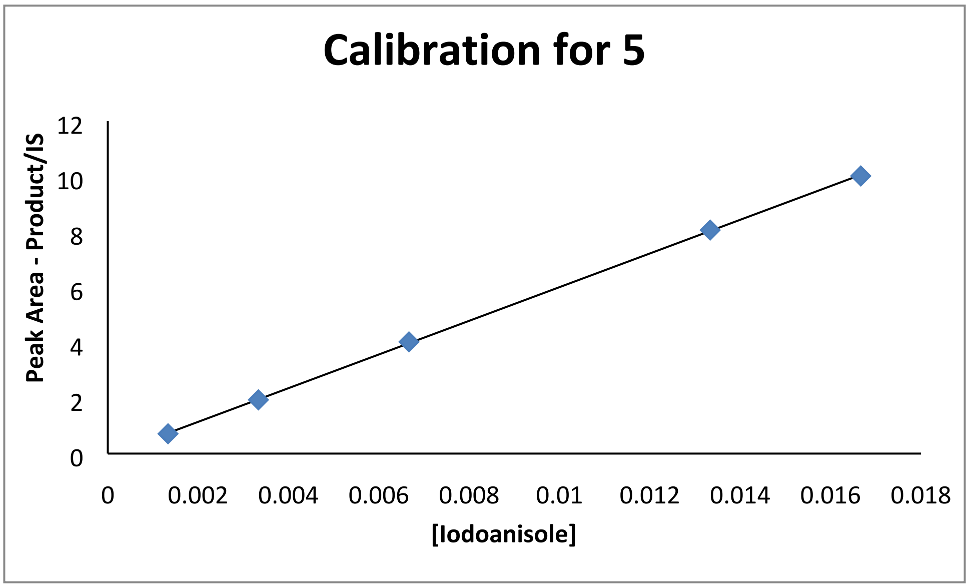

10.2 Mass spectrometry experiments for base-less reaction

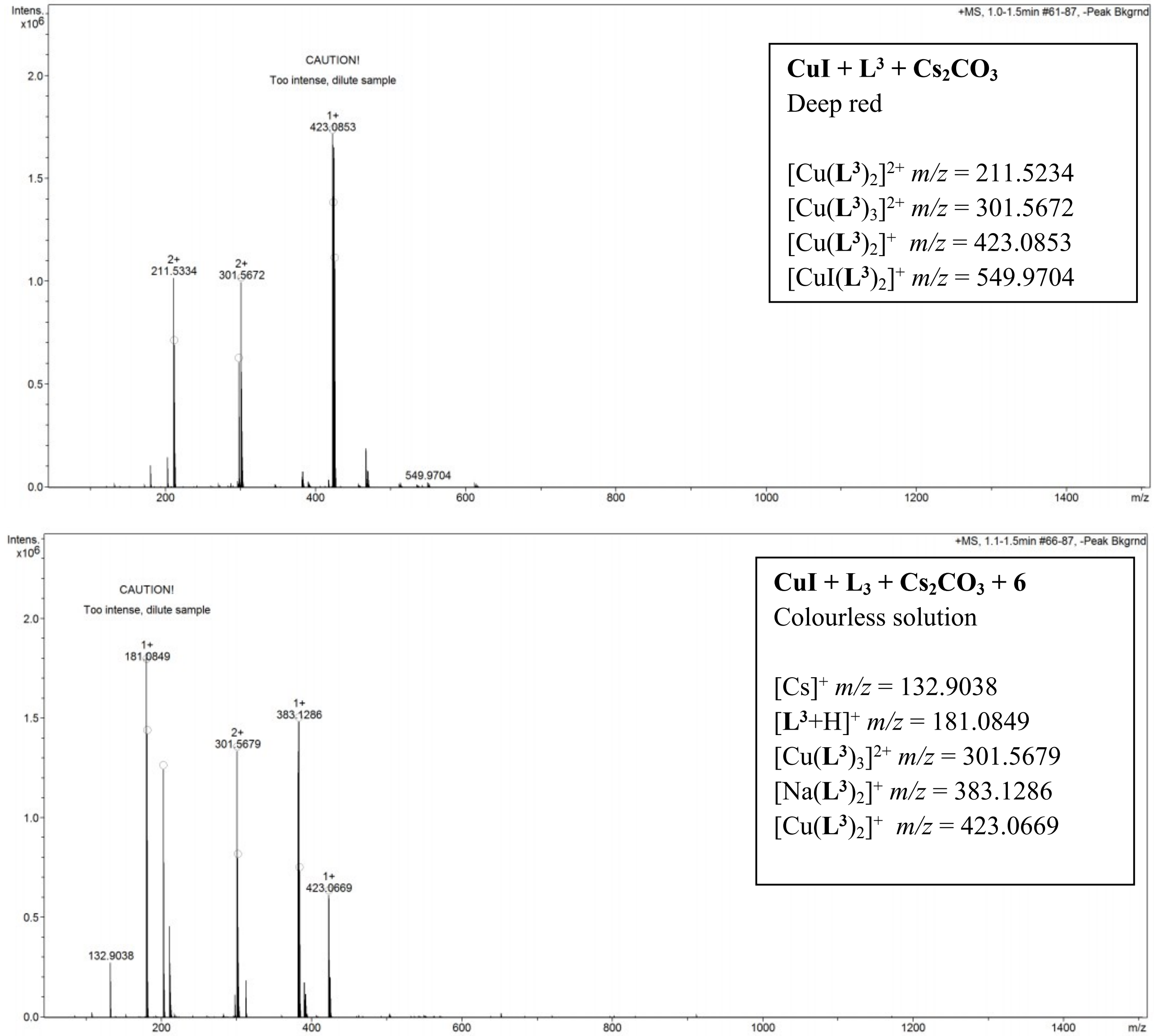

Figure S39. Positive ion HRMS of a mixture of CuI, $\mathbf{L_3}$ and Cs_2CO_3 in DMF before and after treatment with salt 6, showing consumption of $[Cu(\mathbf{L_3})_2]$+ and formation of the deactivated complex **II** (observed indirectly through the formation of $\mathbf{L^3}$ and $[Na(\mathbf{L^3})_2]^+$).

10.3 Reaction Procedures

10.3.1 Sampling air-sensitive reactions

To ensure no oxygen is introduced into the reaction system by standard sampling techniques, enough needles for the desired amount of samples are introduced prior to the vacuum/inert gas cycles, with each needle thoroughly flushed. The set-up as shown below has been found to be a simple method of obtaining kinetic data with these reactions.

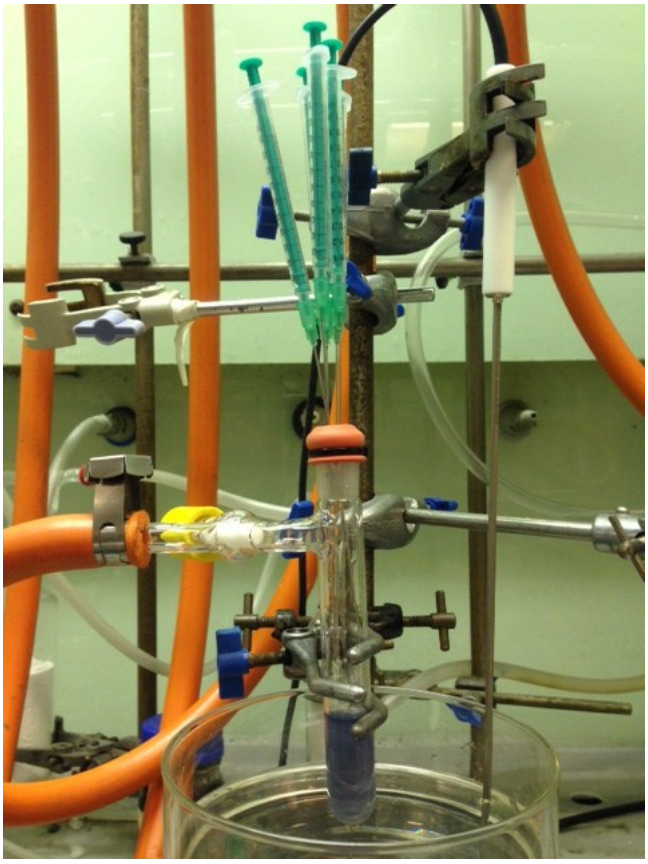

Figure S40. Experimental setup for sampling of highly air-sensitive coupling reaction

10.3.2 Experiment with a single addition of 6

CuI (19 mg, 0.1 mmol), 1,10-phenanthroline **L**[3] (36 mg, 0.2 mmol), 1,1'-biphenyl (15.4 mg, 0.1 mmol) and 4-iodoanisole **1** (234 mg, 1 mmol) were added to a flame-dried Schlenk flask under nitrogen. Three purge/flush cycles were performed prior to addition of degassed DMF (2.5 mL). In a separate flask, Na-pyrrolidinone **6** (160 mg, 1.5 mmol) was mixed with degassed DMF (2.5 mL) under nitrogen to give a suspension. Both flasks were heated to 90 °C and the Na-pyrrolidinone **6** suspension was added in three portions at 0, 1 and 2 hours. The reaction was stopped after 20 hours and GC analysis showed 60 % conversion to product.

10.3.3 Experiment with portion-wise addition of 6

The same procedure as above was performed with three additions of added dropwise over 5 minutes which is represented by the blue line in Figure S37. Samples were quenched by exposure to air and dilution with MeCN, GC analysis was used to give conversion data and a final sample at 20 hours gave 60 % conversion to product.

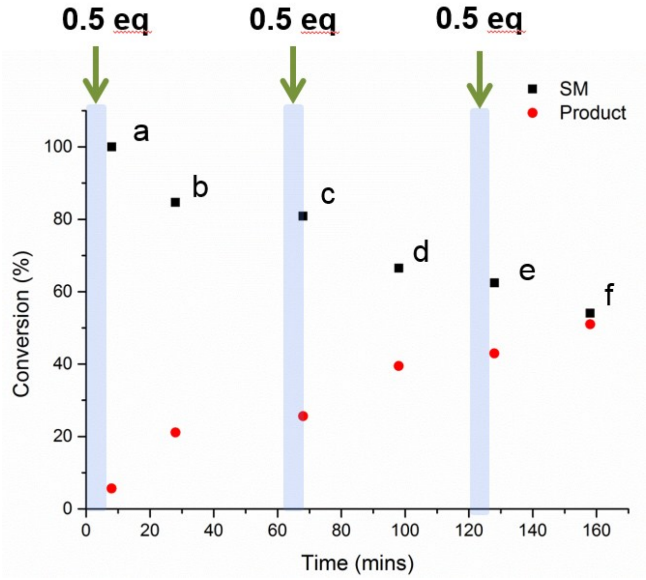

Figure S41. Conversion of **1** and **6** to C-N coupling product **5** following portion-wise additions of salt **6** as a suspension

11. Base Solubility by ^{133}Cs NMR

^{133}Cs NMR spectra were collected at 90 ˚C in DMF. An insert tube was used to include 0.1 M aqueous solution of $CsNO_3$ as external standard, while preventing Cs exchange between the standard and the sample.

Samples were prepared by stirring saturated solutions of CsI, $CsHCO_3$ and Cs_2CO_3 (Acros Organics, Lot A0359643) in anhydrous DMF at 90 °C for 2 hours. After the mixture was allowed to settle, the supernatants (0.5 mL) were transferred to an NMR tube via a syringe fitted with a metal needle (pre-flushed with hot DMF). An insert tube filled with 0.1 M $CsNO_3$ in D_2O was inserted into the NMR tube and the samples kept at 90 °C. The samples were inserted to the NMR spectrometer at 70 °C and quickly heated to 90 °C before data acquisition.

The ^{133}Cs NMR data (Figure S38), referenced to the 0.1 M $CsNO_3$ standard, showed that the saturated concentrations of the caesium salts are:

**Salt**	**Saturated concentration in DMF at 90 °C (M)**
Cs_2CO_3	0.05
$CsHCO_3$	0.12
CsI	1.92

The saturated concentration of CsI was much higher than those of Cs_2CO_3 and $CsHCO_3$. Thus, monitoring Cs content of the reaction mixtures does not reflect the solubility of Cs_2CO_3 in these systems.

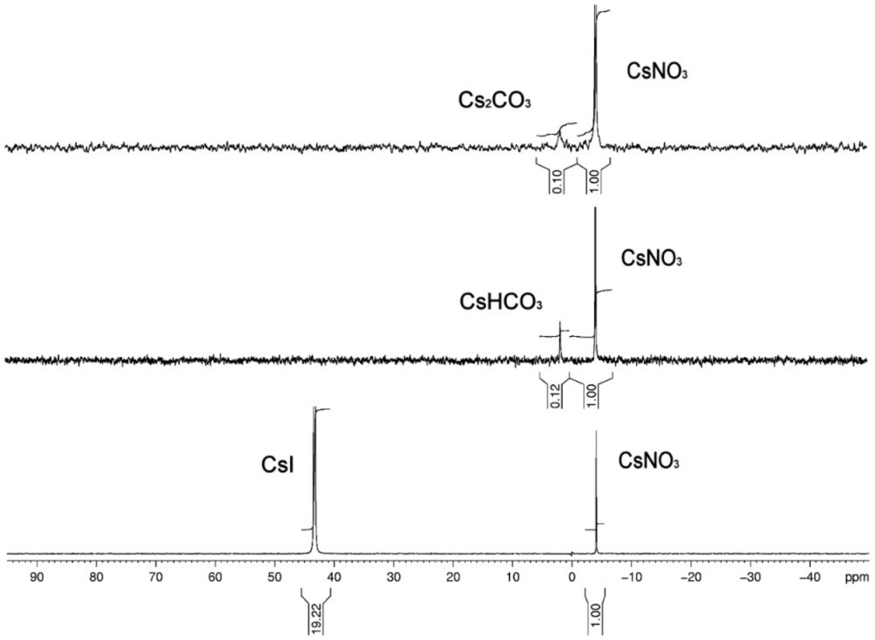

Figure S42. [133]Cs NMR spectra of saturated solutions of CsI, Cs_2CO_3 and $CsHCO_3$ in DMF at 90 °C

12. References

1. C. T. Yang, Y. Fu, Y. B. Huang, J. Yi, Q. X. Guo and L. Liu, *Angew. Chem. Int. Ed.*, 2009, **48**, 7398-7401.
2. S. Y. Lin, R. I. Duclos and A. Makriyannis, *Chem. Phys. Lipids*, 1997, **86**, 171-181.
3. J. Jeong, D. Lee and S. Chang, *Chem Commun*, 2015, **51**, 7035-7038.
4. F. F. Yong, Y. C. Teo, G. L. Chua, G. S. Lim and Y. Z. Lin, *Tetrahedron Lett*, 2011, **52**, 1169-1172.
5. E. J. Rayment, N. Summerhill and E. A. Anderson, *J Org Chem*, 2012, **77**, 7052-7060.
6. C. L. Forryan, R. G. Compton, O. V. Klymenko, C. M. Brennan, C. L. Taylor and M. Lennon, *Phys. Chem. Chem. Phys.*, 2006, **8**, 633-641.
